# Alterations of the Human Skin *N*- and *O*-Glycome in Basal Cell Carcinoma and Squamous Cell Carcinoma

**DOI:** 10.3389/fonc.2018.00070

**Published:** 2018-03-21

**Authors:** Uwe Möginger, Sonja Grunewald, René Hennig, Chu-Wei Kuo, Falko Schirmeister, Harald Voth, Erdmann Rapp, Kay-Hooi Khoo, Peter H. Seeberger, Jan C. Simon, Daniel Kolarich

**Affiliations:** ^1^Department of Biomolecular Systems, Max Planck Institute of Colloids and Interfaces, Potsdam, Germany; ^2^Institute of Chemistry and Biochemistry, Freie Universität Berlin, Berlin, Germany; ^3^Department of Dermatology, Venerology and Allergology, Leipzig University Medical Center, Leipzig, Germany; ^4^Department of Bioprocess Engineering, Max Planck Institute for Dynamics of Complex Technical Systems, Magdeburg, Germany; ^5^glyXera GmbH, Magdeburg, Germany; ^6^Institute of Biological Chemistry, Academia Sinica, Taipei, Taiwan; ^7^Institute for Glycomics, Griffith University, Southport, QLD, Australia

**Keywords:** non melanoma skin cancer, basal cell carcinoma, squamous cell carcinoma, skin glycans, glycosylation, glycomics, oligomannose

## Abstract

The glycome of one of the largest and most exposed human organs, the skin, as well as glycan changes associated with non-melanoma skin cancers have not been studied in detail to date. Skin cancers such as basal cell carcinoma (BCC) and squamous cell carcinoma (SCC) are among the most frequent types of cancers with rising incidence rates in the aging population. We investigated the healthy human skin *N*- and *O*-glycome and its changes associated with BCC and SCC. Matched patient samples were obtained from frozen biopsy and formalin-fixed paraffin-embedded tissue samples for glycomics analyses using two complementary glycomics approaches: porous graphitized carbon nano-liquid chromatography electro spray ionization tandem mass spectrometry and capillary gel electrophoresis with laser induced fluorescence detection. The human skin *N*-glycome is dominated by complex type *N*-glycans that exhibit almost similar levels of α2-3 and α2-6 sialylation. Fucose is attached exclusively to the *N*-glycan core. Core 1 and core 2 type *O*-glycans carried up to three sialic acid residues. An increase of oligomannose type *N*-glycans and core 2 type *O*-glycans was observed in BCC and SCC, while α2-3 sialylation levels were decreased in SCC but not in BCC. Furthermore, glycopeptide analyses provided insights into the glycoprotein candidates possibly associated with the observed *N*-glycan changes, with glycoproteins associated with binding events being the most frequently identified class.

## Introduction

Skin is the largest human organ covering an area of up to two square meters fulfilling vital functions such as protection against pathogens, prevention of water loss, and provision of a sensation platform. It is a widely accepted fact that frequent exposure of skin to ultraviolet radiation is a major risk factor in the development of skin cancers (1, 2) one of the most prevalent types of cancer in Caucasians (3). Basal cell carcinoma (BCC) and squamous cell carcinoma (SCC) are the more frequently occurring types of skin cancer next to the highly aggressive malignant melanoma type skin cancers. There are an estimated three million people diagnosed with some form of BCC or SCC each year in the US alone (4) posing a serious health burden as they are causing severe local damage if left untreated.

A cell’s transformation into a malignant state is accompanied by numerous changes on a molecular level including changes in protein glycosylation (5), one of the most common types of post translational modifications. Protein glycosylation is the result of a complex enzymatic network that is regulated by numerous intrinsic and extrinsic factors. Changes in glycosyltransferase levels or activities and monosaccharide substrate availability are just a few examples out of the numerous factors that ultimately result in altered glycosylation patterns that are frequently reported for malignant cells (6). As glycans play important roles in cell adhesion, cell signaling as well as in adaptive and innate immune responses (7, 8), aberrant protein glycosylation patterns represent a recurring feature reported for all types of cancer studied to date (5).

Changes in glycosylation-associated pathways are often reflected in up- or down regulation of glycan structure features such as sialic acid (9), bisecting *N*-acetylglucosamine, Lewis structures, core-type fucose, or β1-6-linked branching (10, 11). Changes in glycosylation can alter the functional properties of their individual protein carriers affecting tumor growth, formation of metastases, escaping immune recognition or conferring resistance to therapeutic interventions (12–14). The introduction of a β1-6 branch can alter subsequent glycan processing by mediating the addition of poly-*N*-acetyllactosamine residues. Due to the configuration of the β1-6 branch interactions with the polypeptide chain are possible that alter protein properties (15). An increase in the levels of sialic acid (16) or Lewis structures can affect lectin interactions, which can significantly alter vital functions such as cell signaling, cell adhesion or cell motility. *O*-glycans on the other hand are often observed as truncated structures only consisting of one or two monosaccharide units such as the Tn and T antigen (17). Both these antigens play a major role as possible anticancer vaccines, and increased Tn/T-antigen levels often correlate with poor disease prognosis. Other cancer-associated glycan changes have also been recognized for their potential diagnostic value in tumor detection and evaluation of tumor growth and progression (18–20). This makes glycans and their specific tumor signatures highly interesting to gain a better understanding of the molecular events associated with tumor onset and progression. Even though skin represents the most exposed organ surprisingly little information is available concerning the protein glycosylation repertoire of healthy human skin and its alterations in non-melanoma skin cancer. To fill this gap, we used porous graphitized carbon liquid chromatography (PGC-LC) coupled with electrospray ionization (ESI) ion-trap tandem MS (ESI-MS/MS) detection and multiplexed capillary gel electrophoresis with laser induced fluorescence detection (xCGE-LIF) (21) to investigate the healthy human skin *N*- and *O*-glycome and the alterations associated with BCC and SCC. Moreover, to obtain insights into the identity of the glycoproteins carrying these *N*-glycans hydrophilic interaction chromatography (HILIC) enriched (glyco)peptides fractions were analyzed by nanoLC ESI Orbitrap tandem mass spectrometry (MS/MS).

## Materials and Methods

### Consumables

All materials were purchased in the highest possible purity from Sigma-Aldrich (St. Louis, MO, USA) unless stated differently. Glycerol-free peptide-*N*-Glycosidase F (PNGase F) was obtained from Roche Diagnostics GmbH (Mannheim, Germany). Water was taken from a MilliQ-Direct 8 system (Merck KGaA, Darmstadt, Germany).

### Patient Samples

The study was approved by the institutional review board of the medical faculty at the University of Leipzig (No. 127-11-18042011). Patients referred to the Department of Dermatology at the Leipzig University Medical Center for surgical treatment of BCC or SCC were asked to participate on a voluntary base and were included after written informed consent. To allow correct dermato-pathological investigation, only patients were selected that exhibited a well-circumscribed BCC or SCC of at least 5 mm diameter. Following written informed consent, basal cell or SCCs were routinely excised (fusiform excision) in local anesthesia with micrographic control of the margins. Punch biopsies were taken from tumor and normal, healthy skin of the excised tissue, snap frozen in liquid nitrogen, and then stored at −80°C. The remaining material was subjected to the routine dermato-pathological work up. Biopsies were transferred on dry ice to the Max Planck Institute of Colloids and Interfaces for further analyses.

In addition to the fresh frozen samples, formalin-fixed paraffin-embedded (FFPE) tissue from 20 patients with BCC and 15 patients with SCC was obtained following routine dermato-pathological work up. Tissue slides containing tumor tissue and slides of the tumor free margins (=healthy skin) were provided respectively. More detailed patient information can be found in Tables S4–S6 in Supplementary Material.

### Biopsy Sample Homogenization and Protein Extraction

Biopsy tissue samples were washed three times with 1 mL 70% ethanol (EtOH) and three times with 50 mM NH_4_HCO_3_. The sample was homogenized in 1 mL of homogenization buffer [50 mM NH_4_HCO_3_, 1 M urea, 10% ACN, and 0.1% sodium dodecyl sulfate (SDS)] for (3× 30 s) using a homogenizer (Ultraturrax, IKA, Staufen, Germany) with subsequent sonication for 30 s. Samples were kept at 0°C during the entire homogenization process before any protease, and glycosidase activity was terminated by incubating them for 5 min at 96°C. To increase protein/peptide solubility, 4 μg of trypsin was added, and the samples were incubated over night at 37°C before insoluble particles were removed by centrifugation at 12,000 rpm for 30 min. The supernatant was collected and delipified by adding 400 μL chloroform, rigorous vortexing, and centrifugation at 12,000 rpm for 5 min. The lipid phase at the interphase was discarded. The supernatant and the chloroform phase were combined, lyophilized, and subsequently stored at −20°C until further use. For dot blotting onto PVDF membranes, freeze-dried samples were resuspended in 200 μL of 8 M urea.

### Protein Extraction From FFPE Histopathological Slides

Antigen retrieval from FFPE slides was performed as described earlier (22). In brief, tissue samples were washed directly on the glass slides using a washing chamber with three alternating washing steps using xylene, followed by EtOH (2 min each). The tissue section was then detached with a sharp blade, transferred into a sample tube and subsequently 200 μL buffer (4% SDS in 0.1 M Tris–HCl, pH 8, 0.1 M dithiothreitol) were added. The suspension was sonicated on ice for 15 s at level 1.5 (Branson Sonic Power Company, Danbury, CT, USA) and subsequently incubated in a thermoshaker at 99°C at 600 rpm for 60 min. The sample was allowed to set to room temperature before it was centrifuged at 2,000 *g* for 20 min. The supernatant was transferred into a new vial and dried in a SpeedVac concentrator (Thermo, Bremen, Germany). Prior dot blotting onto PVDF membranes, samples were dissolved in 20 μL 8 M urea.

### Glycan Release

The extracted proteins/peptides were blotted onto a PVDF membrane (0.2 μm pore size, Millipore, Tullagreen, Ireland) in five 1 μL steps allowing the droplet to dry between each blotting step. *N*- and *O*-glycans were sequentially released, reduced, and purified as described in earlier work (23).

In brief: protein/peptide sample solution was dot blotted onto a PVDF membrane. The membrane was washed in methanol and water for 15 min each. Spots were cut out and blocked for 5 min in 100 μL 1% polyvinyl pyrrolidone solution (PVP40, v/v). PVP was removed, and the membrane spots were washed three times with water. *N*-glycans were released by addition of 3 U of PNGase F (Roche, Mannheim, Germany) in 10 mM NH_4_HCO_3_. After removal of the *N*-glycans the *O*-glycans were released by β-elimination in 0.5 M NaBH_4_ in 50 mM KOH. *N*-glycans were reduced for 3 h at 50°C in 20 μL of 1 M NaBH_4_ in 50 mM KOH. The reaction was quenched by the addition of 2 μL glacial acetic acid.

*N*- and *O*-glycans were desalted using filter TopTip (Glygen, Columbia, MD, USA) filled with Dowex cation exchange resin (Dow, Midland, USA). The remaining borate was removed by several rounds of co-evaporation using 100 μL methanol in the SpeedVac concentrator.

### Neuraminidase Digest for PGC-LC ESI-MS/MS Analysis

The glycan sample was reconstituted in 10 μL 25 mM ammonium acetate buffer (pH 5.5) and 25 U of a α2-3-specific neuraminidase (NEB, Ipswitch, MA, USA) were added. The sample was incubated for 3 h at 37°C. The reaction was quenched by heating to 70°C for 5 min followed by a carbon clean up as described earlier (23).

### Glycopeptide Sample Preparation

Patient samples were pooled for glycoproteomics analysis, and the peptide/protein concentration was estimated by measuring the absorbance at a wavelength of 280 nm in a Nanodrop ND-1000 spectrophotometer (Thermo Fisher Scientific) according to the manufactures instructions.

Proteins from pooled snap frozen biopsies were digested with trypsin using two approaches: *in liquid* as well as after having been blotted onto PVDF membrane. Pooled FFPE samples were digested on PVDF membrane. Peptide recovery from the PVDF membrane was achieved by three extractions with 50% ACN, one with 100% ACN followed by a final extraction with 5% formic acid (FA). The steps were repeated, and the extracts were combined. Glycopeptides were enriched using self-packed zwitterionic hydrophilic interaction liquid chromatography stagetips (10 μm particle size, 100 Å pore size, SeQuant AB, Sweden). The column was washed three times with 0.1% trifluoroacetic acid (TFA) and equilibrated three times with 80% ACN/0.1% TFA. Samples were reconstituted in 10 μL 0.1% TFA and brought to a concentration of 80% ACN/0.1% TFA by slowly adding 40 μL of 100% ACN/0.1% TFA. Each sample was applied twice to the column. Samples were washed twice with 50 μL of 80% ACN/0.1% TFA and eluted twice with 50 μL of 0.1% TFA followed by 50 μL of 80% ACN/0.1% TFA.

Before liquid chromatography (LC)–MS analysis the sample were run through a C_18_-ZipTip (Merck Millipore, Tullagreen, Ireland) for sample clean up and to avoid sample overload. The amount of protein in the eluted fraction was assumed to be equal to the 5 μg binding capacity of ZipTip. The eluted fraction was reconstituted in 10 μL 0.1% FA, and 3 μL was injected for LC–MS analysis.

### Porous Graphitized Carbon (PGC)-nanoLC ESI-MS/MS Glycomics

Porous graphitized carbon-nanoLC ESI-MS/MS glycomics was performed using an amaZon ETD speed ion trap (Bruker, Bremen, Germany) coupled to an Ultimate 3000 UHPLC system (Dionex/Thermo). The deprotonated ions were detected in negative ion mode, and the instrument was tuned to perform CID fragmentation on the three most intense precursors of each MS scan. An *m*/*z* range from 350–1,800 was used for data-dependent precursor scanning. MS as well as MS/MS data were recorded in the instrument’s “ultra-scan mode.” Detailed information on the MS settings is found in Table S3 in Supplementary Material.

*N*-glycans were loaded onto a PGC precolumn (Hypercarb™ KAPPA 30 × 0.32 mm, 5 μm particle size) and separated on an analytical PGC column (Hypercarb™ PGC Column, Thermo, 100 mm × 75 μm, 3 μm particle size). The samples were loaded onto the precolumn at a flow rate of 6 μL/min in 98% buffer A (10 mM NH_4_HCO_3_). The analytical column was equilibrated in 3% buffer B (10 mM NH_4_HCO_3_ in 60% ACN) at a flow rate of 1 μL/min. Buffer B increased from 3 to 15.8% (5–6 min), then to 40.2% B (6–67.25 min) and then to 55% B (67.25–78 min). The concentration was further increased to 95% B over 1 min and held at 95% B for 5 min (79–84 min) while the precolumn was flushed with 90% buffer C (10 mM NH_4_HCO_3_ in 90% ACN) at a flow rate of 6 μL/min before both columns were re-equilibrated for 5 min in 98% buffer A before the next sample was loaded. Generally, the MIRAGE guidelines were followed (24, 25).

### xCGE-LIF Analysis and Sample Preparation

As glycans need to be unreduced so that the fluorescent label can be attached *via* reductive amination for subsequent xCGE-LIF analyses only *N*-glycans were analyzed by this method. Sample preparation and xCGE-LIF measurement was performed as described earlier (21, 26, 27). A detailed description of sample preparation and xCGE-LIF measurement conditions is listed in Supplementary Material.

### LC-Orbitrap Glycoproteomics

Glycopeptide analyses were performed in the positive mode on an Orbitrap Fusion™ Tribrid™ mass spectrometer (Thermo Fisher Scientific, San Jose, CA, USA) coupled to an Ultimate 3000 UHPLC system (Dionex/Thermo). The instrument was tuned to perform HCD-product dependent (pd)-CID MS^2^ fragmentation, namely additional ion-trap CID MS^2^ was only triggered when glycopeptide oxonium ions (204.0867 and 366.1395) were detected in the HCD analyses. An *m*/*z* range from 400 to 2,000 Da was used for data-dependent precursor scanning. Peptides were directly loaded onto the analytical column (Acclaim PepMap RSLC C18, 2 μm, 100 Å, 75 μm × 25 cm) in 100% buffer A (99.9% v/v water, 0.1% v/v FA). A 90-min gradient of increasing buffer B (99.9% v/v ACN, 0.1% FA) was developed at a flow rate of 500 nL/min: 5% at 2 min, 35% at 62 min followed by an increase to 85% buffer B at 65 min. The column was held at 85% for another 10 min before it was re-equilibrated in 100% buffer A for 15 min. More detailed information on all instrument settings can be found in Supplementary Material.

### Glycan Analysis and Quantification

Mass spectrometry *N*- and *O*-glycan spectra were evaluated manually by Compass DataAnalysis version 4.2 (Bruker). The data were then loaded into QuantAnalysis version 2.2 (Bruker) where quantitation was performed on extracted ion chromatograms (XIC) including all detected charge states of the respective glycan. A smoothing width of 3 s (Gauss algorithm) was applied on each XIC.

Data from xCGE-LIF aquisition data was analyzed using the Java-based glycan analysis software glyXtool™ (glyXera, Magdeburg, Germany). Data were normalized to an internal standard, transforming electropherograms to “*N*-glycan fingerprints” (see Figure S9 in Supplementary Material). Automated peak picking, integration, and relative quantification were performed, followed by *N*-glycan assignment to peaks *via* migration time matching to an in-house *N*-glycan database. *N*-glycan sequences and linkages were confirmed by exoglycosidase digests (see Supplementary Material).

### Protein Identification Parameters

For skin protein identification, the data were processed with Thermo Proteome Discoverer (version 1.4.1.14) using MASCOT (version 2.5.1) with the following settings: enzyme: trypsin; variable modification: carbamidomethyl (C), deamidated (N, Q), Oxidation (M); peptide tolerance: 10 ppm, fragment tolerance: 0.02 Da; missed cleavages: 1; database: SwissProt 2015_12 (550,116 Sequence); taxonomy: human (20,268 sequence). The subsequent glycopeptide analysis by Byonic (version 2.7.4) used the following settings: maximum number of missed cleavages: 2; fragmentation type: QTOF/HCD; precursor tolerance: 10 ppm; fragment tolerance: 20 ppm.

### Statistical Evaluation

Statistical analysis was partly performed using “R” version 3.0.2 (2013-09-25) using the “gplots” package^1^ version 2.16.0 as well as the “pROC” package^2^ version 1.9.1. The heatmap was generated using the logarithmic relative abundance values to calculate the Euclidean distance matrix and complete linkage clustering. Values in the heatmaps were scaled individually for each *N*-glycan structure. Statistical test were performed with performed with SigmaPlot software version 13.0. Samples were tested for normal distribution by Shapiro–Wilk test, significance was determined by *t*-test or Mann–Whitney test (if samples were not normal distributed). *p*-Values below or equal to 0.04 were considered significant. Bean and violin diagrams were created with the free online tool BoxPlotR (Version 3.1 and shiny-server 1.2).

## Results

A detailed workflow overview is described in Figure S1 in Supplementary Material.

### Healthy Human Skin Glycoproteins Carry Mainly Sialylated *N*-Glycans

**Figure 1:**
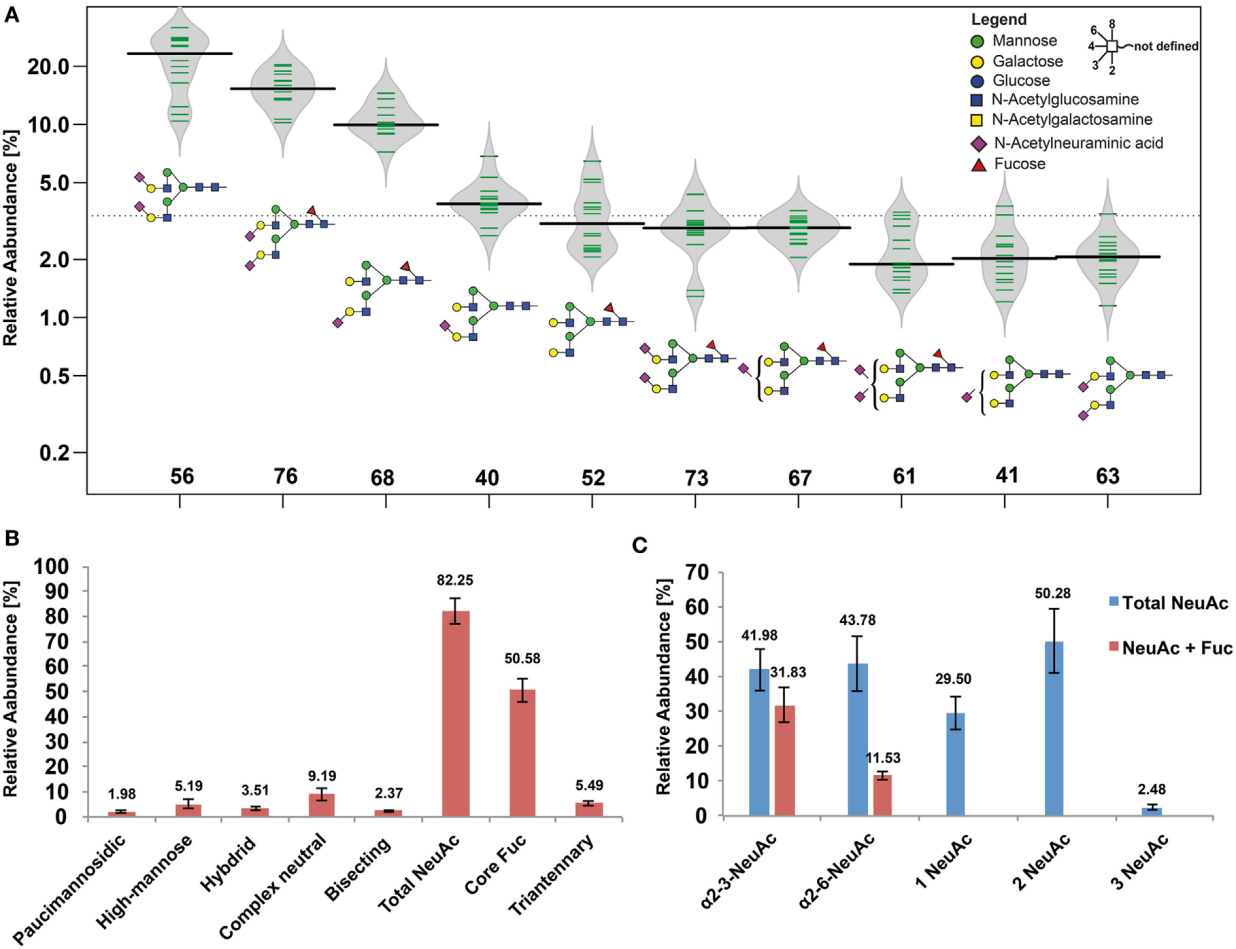
Healthy human skin *N*-glycome. **(A)** Bean diagram representing the 10 most abundant *N*-glycans determined from 14 patients. Green bars indicate individual data points, the black line represents the median, and the gray area depicts the data density. Columns indicate the glycan structures given by their glycan ID (Table S1 in Supplementary Material). **(B)** Relative *N*-glycan class abundances found in healthy skin biopsies. Sialylated and fucosylated structures were the major components representing the human skin *N*-glycome. **(C)** Relative abundances of different structure features found on sialylated *N*-glycans. Blue bars represent sialylated *N*-glycans with and without core fucose depending on their sialic acid linkage, showing that core fucosylation was a more abundant feature on *N*-glycans carrying one or two α2-3 linked *N*-acetylneuraminic acid (NeuAc) residues (if both, α2-3 and α2-6 linkages were present on one *N-*glycan, this *N*-glycan was considered in both linkage categories). Overall, α2-6-linked NeuAc was a slightly more abundant feature compared with α2-3-linked NeuAc. Most of the sialylated *N*-glycans carried two NeuAc residues. Tri-antennary species were below 3% and rather low abundant using porous graphitized carbon, while multiplexed capillary gel electrophoresis with laser induced fluorescence detection (xCGE-LIF) detected slightly higher levels of tri- and tetra-antennary *N*-glycans (see Supplementary file “xCGE-LIF quant.xlsx” in Supplementary Material).

Skin biopsies obtained from healthy skin areas of 14 BCC patients were used to establish the glycome reflecting healthy human skin. We used a PGC-nano-UHPLC ESI-MS/MS- and the orthogonal xCGE-LIF-based glycomics workflow. Both approaches allow for the separation of glycan structure isomers enabling differentiation of features such as α2-3, α2-6 linkages of sialic acid or core and arm fucosylation (Figures S6 and S7 in Supplementary Material) (21, 26, 28–30). The human skin *N*-glycome consisted of 107 identified structures that were very similar within the analyzed patient cohort (Figure 1A; Table S1 in Supplementary Material). Expected patient-specific variability in individual *N*-glycan abundances was in particular found for the high abundant *N*-glycans (Figure 1A). Biantennary, sialylated *N*-glycans were the dominating structures, comprising on average about 82% of the human skin *N*-glycome (Figure 1B). Human skin *N*-glycans exhibited an equal distribution of α2-3- and α2-6-linked *N*-acetylneuraminic acid (NeuAc) residues (Figure 1B), which is in contrast to human serum where *N*-glycans are mainly carrying α2-6-linked NeuAc on the non-reducing end (23, 31). Oligomannose type (5%), hybrid type (3%), bisected (2.6%), and paucimannosidic (1.4%) *N*-glycans overall were just minor components of the human skin *N*-glycome (Table S1 in Supplementary Material). Overall, the data between the PGC-LC ESI-MS/MS- and the xCGE-LIF-based glycomics were in good agreement with respect to identified *N*-glycan structures, sialic acid and fucose linkage, and relative quantitation.

### Core Fucosylation Is Markedly Increased on α2-3-NeuAc Carrying *N*-Glycans

Approximately half of all detected human skin *N*-glycans were fucosylated with the fucose residue being almost exclusively attached to the *N*-glycan core. While this modification occurred roughly on a quarter of all α2-6 sialylated *N*-glycans it was present on 75% of all α2-3 sialylated *N*-glycans (Figure 1B). Our data indicate that in human skin core-fucosylated *N*-glycans are the preferred substrate for α2-3 sialyltransferases, while α2-6 sialyltransferases appear to prefer non-core-fucosylated *N*-glycan substrates (Figure 1C). Roughly 90% of neutral *N*-glycans were core-fucosylated, indicating that core fucosylation occurs before sialylation and that a competitive interplay between α2-6 sialyltransferase and core-fucosyltransferase activity might take place in the Golgi, subsequently resulting in the observed skin *N*-glycome patterns.

### The Healthy Human Skin *O*-Glycome Consists of Simple Core 1 and 2 Type *O*-Glycans

**Figure 2:**
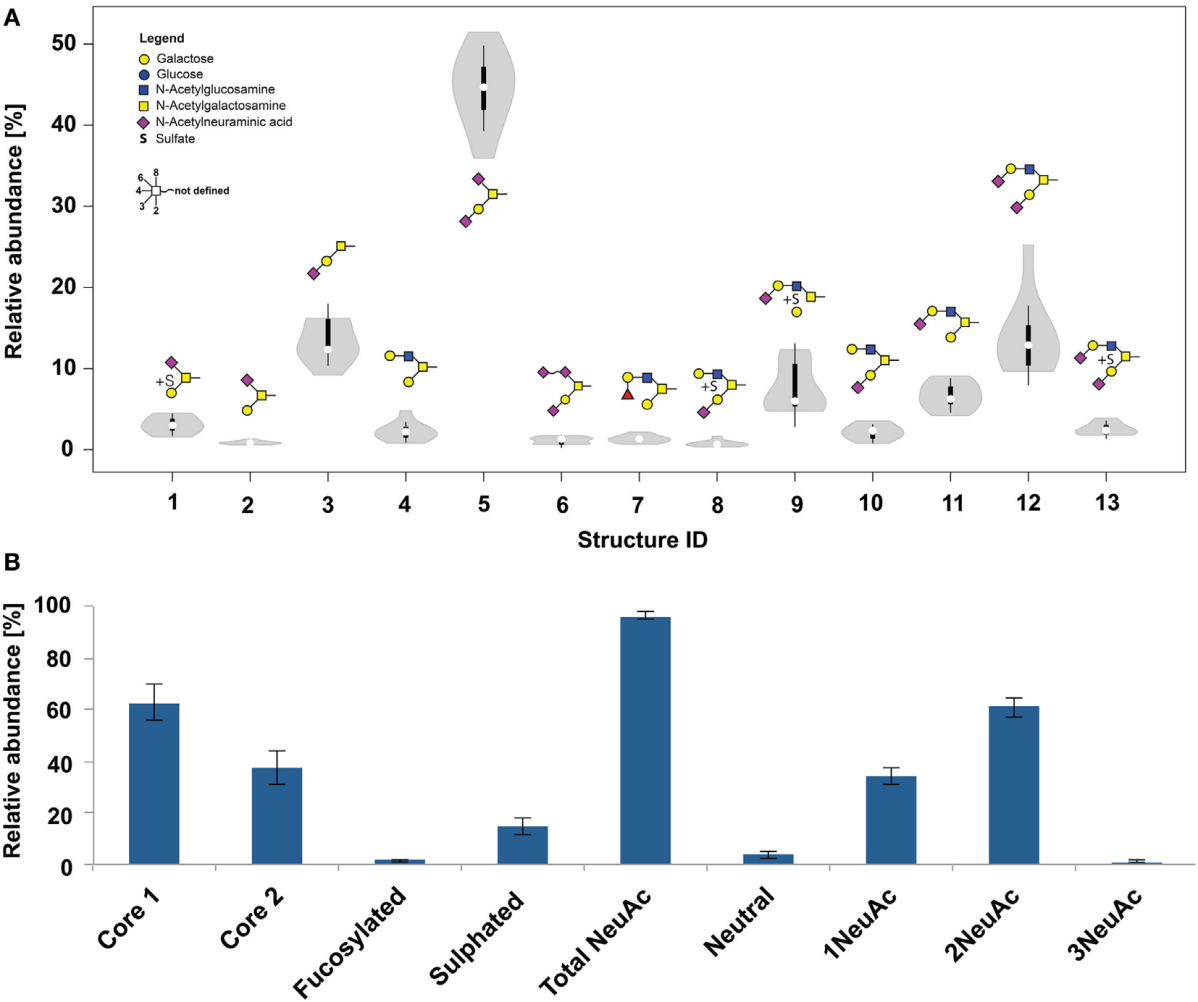
**(A)** Violin diagram showing the distribution of *O*-glycan structures within nine patients (thickness indicates the majority of the data points). Columns indicate the glycan structures given by their glycan ID (Table S2 in Supplementary Material). **(B)** *O*-glycan composition of healthy frozen skin biopsies from nine patients.

After the enzymatic removal of *N*-glycans *O*-glycans from nine healthy skin samples were released by reductive β-elimination. With just 13 detected structures of the core 1 and core 2 type, the skin tissue *O*-glycome was clearly less diverse (Figure 2). Sialylation was a major structural feature with roughly 96% of the glycans carrying at least one NeuAc residue (Figure 2B). Three structures (ID 5; ID 3 and ID 12, Table S2 in Supplementary Material) contributed to more than 60% of the total skin *O*-glycome. Interestingly, the presence of a di-sialic acid residue on a core 1 *O*-glycan was confirmed on a triply sialylated *O*-glycan (ID 6, Figure S2 in Supplementary Material). Treatment with α2-3-NeuAc-specific neuraminidase removed a single NeuAc residue, but did not result in the loss of the diagnostic *m*/*z* = 581.05 B-ion signal that is indicative for a NeuAc-NeuAc disaccharide fragment (Figure S2 in Supplementary Material). These exoglycosidase digestion data also indicated that the di-sialo epitope was attached in the C6 position to the reducing end core GalNAc. Its resistance to α2-3-specific neuraminidase digestion also indicated that these two NeuAc residues are linked *via* an α2-8 or α2-9 linkage.

### Oligomannose *N*-Glycan Levels Increase in BCC

**Figure 3:**
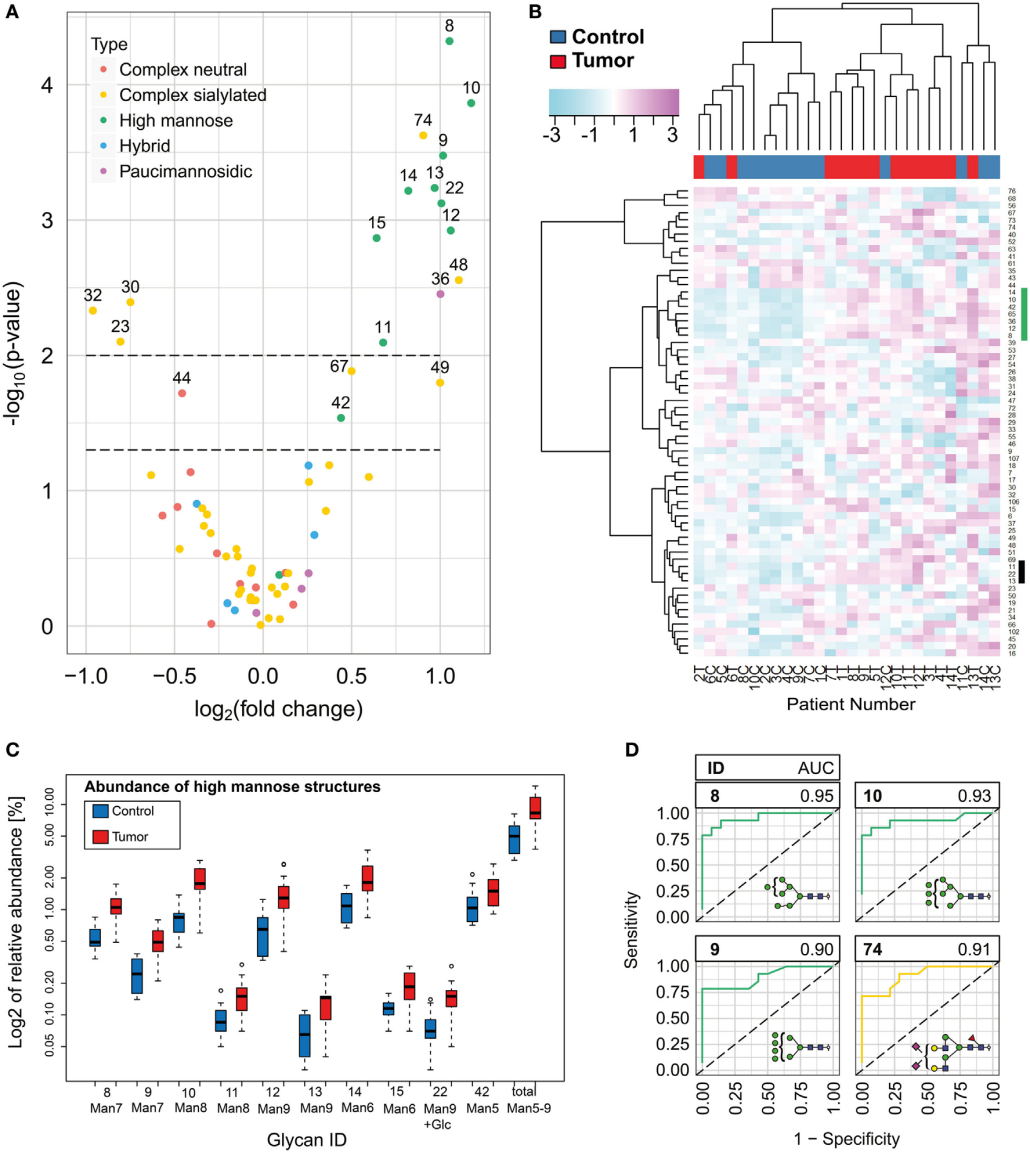
*N*-glycan level changes in basal cell carcinoma. **(A)** Volcano plot for the quantified *N*-glycan species indicating significant changes of relative abundance levels for individual *N*-glycan species. Two *p*-value thresholds are indicated (*p* = 0.05 and *p* = 0.01). *N*-glycan abundance levels above the second threshold were considered as significantly changed. **(B)** Unsupervised hierarchical cluster analysis of healthy and tumor skin. Rows display each of the 14 patient *N*-glycan data (T, tumor tissue; C, healthy control). Columns indicate the glycan structures given by their glycan ID (Table S1 in Supplementary Material). Based on the *N*-glycan data, the algorithm classified the majority of the healthy and tumor skin samples into two major groups. Two glycan clusters (marked by green and black bar) were observed in particular exhibiting increased levels for oligomannose type *N*-glycans. **(C)** Box plot graph representing log2 abundances of oligomannose *N*-glycans and the total of oligomannose structures in normal and tumor tissue. **(D)** ROC of four *N*-glycan structures with the highest area under the curve values.

**Figure 4:**
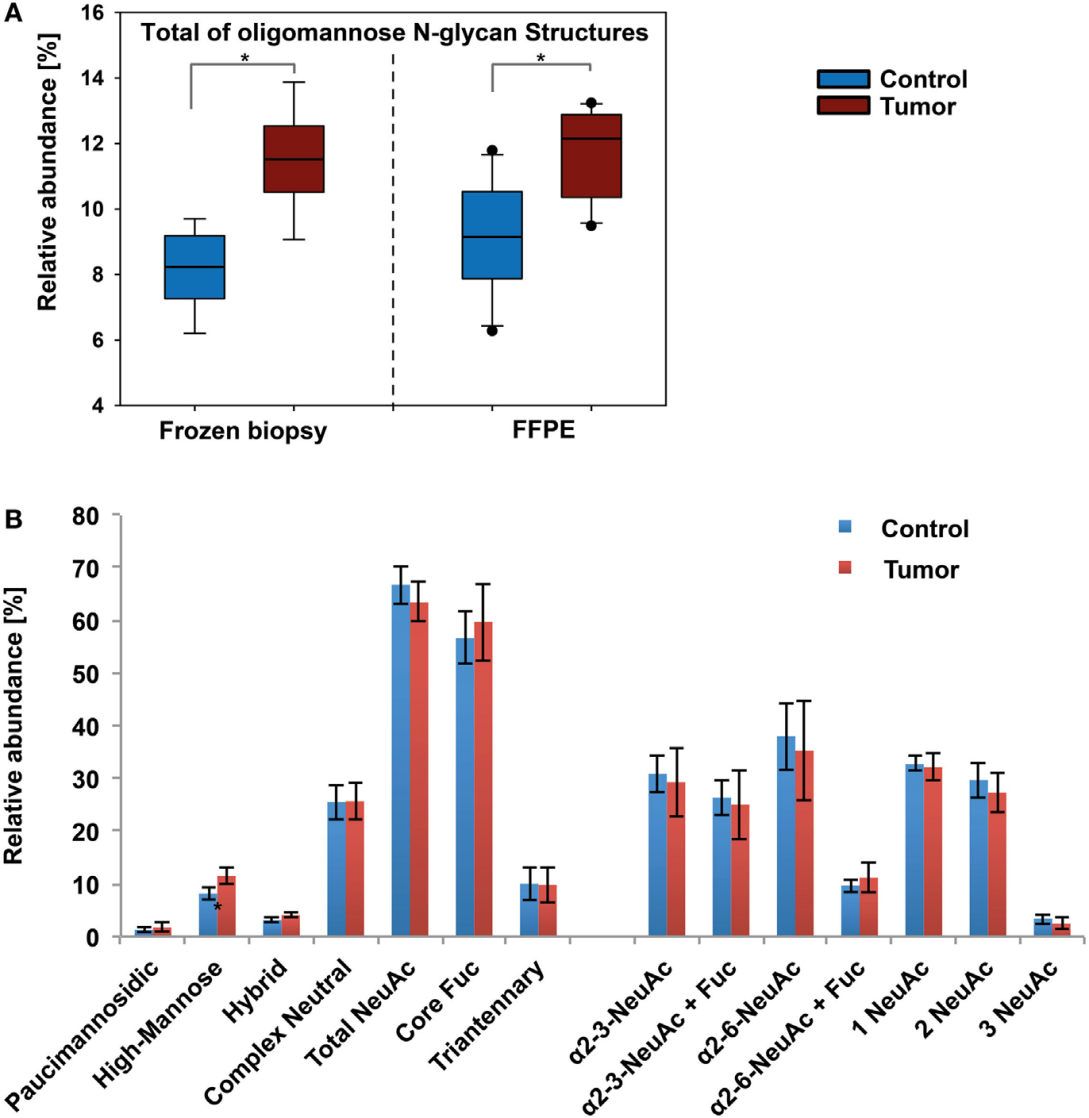
Results from xCGE-LIF *N*-glycomics analyses. **(A)** Oligomannose levels in tumor and healthy tissue show a similar pattern as determined by porous graphitized carbon-nanoLC electrospray ionization ion-trap tandem MS. Significance (*p* ≤ 0.04) is indicated by asterisk (*). **(B)** Distribution of *N*-glycan features in healthy skin and basal cell carcinoma of fresh biopsy samples.

The results obtained for healthy skin were compared with the glycome of BCC from the same patients to minimize effects derived from individual variations. Relative abundance was assessed for 65 most abundant *N*-glycans isolated from snap frozen biopsies. Representation of the data by volcano plot show several *N*-glycan structures that significantly changed in cancer (Figure 3A). Oligomannose type *N*-glycans exhibited the highest fold change (2×) followed by an α2-3/2-6-sialylated structure (ID 74), a bisected, α2-3/2-6-sialylated (ID 48), and a paucimannosidic *N*-glycan (ID 36). By contrast, the abundances of three complex singly sialylated *N*-glycans (ID 23, 30, and 32) decreased in BCC compared with healthy skin. Virtually all oligomannosidic structures showed increase in abundance resulting in the whole class being differentially expressed in BCC (Figure 3C). A consistent change of all members within other major glycan classes such as for instance paucimannosidic structures (ID 32) could not be observed (Figure 4; Figure S3 in Supplementary Material). Furthermore, an unsupervised cluster analysis was performed to evaluate if the glycan data allow distinguishing between healthy and BCC skin tissue (Figure 3B). Three major patient groups became apparent: 2 groups of 12 samples each reflected largely healthy (10 healthy/2 tumor) and tumor skin samples (11 tumor/1 healthy), while the third group consisting of four samples was derived from tissue biopsies classified as healthy (3 samples) and tumor (1 sample) tissue. The cluster analysis further supported the earlier findings that the entire class of oligomannose *N*-glycan showed increased levels resulting in two distinct glycan clusters.

*N*-glycans such as Man9 + Glc, Man9, and Man8, which are also associated with early stages of glycoprotein biosynthesis, exhibited a stronger increase compared with structures representing the next processing stages (Man7, Man6, and Man5).

To determine the distinguishing power of the individual glycan species, we calculated the area under the curve of the receiver operating characteristic (ROC, Figure S8 in Supplementary Material) of each of the glycans showing significant abundance differences. Oligomannosidic *N*-glycans ID 8, 9, 10, and the sialylated complex *N*-glycan 74 exhibited an AUC value above or equal to 0.9 and might provide either alone or in combination valuable diagnostic targets (Figure 3D).

### BCC *N*-Glycan Signatures Are Confirmed in FFPE Tissue Sections

These initial glycomics results obtained for the tissue biopsies were further validated using an independent sample cohort consisting of FFPE histopathological tissue representing corresponding healthy skin and BCC sections from 20 patients. The total number of identified *N*-glycans was lower in the FFPE samples. The results obtained for the FFPE sample cohort confirmed the previous findings of increased oligomannose *N*-glycan levels in BCC. Fourteen out of 20 patient specimens exhibited an average 1.5-fold upregulation of Man9, Man8, Man7, and Man6 in BCC tissue (Figure S4 in Supplementary Material).

Similar to earlier reports (32), minor differences in relative abundance were detected for individual *N*-glycans when comparing the results from fresh tissue biopsies and FFPE histopathological tissue sections. While the relative abundance for α2-3 doubly sialylated fucosylated *N*-glycans (ID 76) was increased, the levels for α2-6 doubly sialylated biantennary non-fucosylated *N*-glycans (ID 56) decreased. In summary, in 70% of the analyzed BCC FFPE tissue sections an increase in oligomannose *N*-glycan levels was confirmed.

### xCGE-LIF Analyses Confirm *N*-Glycan Alterations in BCC

The MS data were additionally cross-validated using an orthogonal approach based on xCGE-LIF detection using sample pairs of nine fresh tissue and 10 FFPE biopsies. In xCGE-LIF, the glycan identities were determined by correlation of the migration time with an in-house database and confirmed using exoglycosidase digestion. Notably, the number of identified *N*-glycans was only slightly lower in the xCGE-LIF analyses. The xCGE-LIF analyses confirmed that significantly higher oligomannose *N*-glycan levels were present in BCC tissue (1.4-fold increase in fresh and 1.3-fold in FFPE tissue). Overall, the abundance levels for the individual *N*-glycan structure classes were comparable between MS- and xCGE-LIF-based analytical approaches. However, the ratio found for neutral and sialic acid containing *N*-glycans showed a notable difference (Figure 4B). While roughly 16% less sialylated *N*-glycans (66%) were found in xCGE-LIF the amount of neutral structures was increased (25%). Partial desialylation may occur during the acidic labeling process (33), which could in fact account for the observed reduced amounts of sialylated *N*-glycans. On the other hand, xCGE-LIF detected some tri- and tetra-antennary *N*-glycans that were not detectable by the PGC-LC ESI-MS/MS setup used in this study, clearly showing the importance of having orthogonal glycomics approaches available for a comprehensive analysis (Table S1 and Supplementary file “xCGE-LIF quant.xlsx” in Supplementary Material).

### Core 2 Type *O*-Glycan Levels Are Increased in BCC

**Figure 5:**
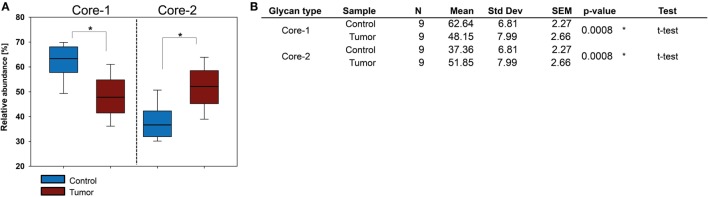
*O*-glycosylation in basal cell carcinoma (BCC) tissue. **(A)** Relative abundance of core 1 and core 2 *O*-glycans in healthy and BCC tumor tissue obtained for nine patients. **(B)** Statistical evaluation of *O*-glycan cores. Significant differences in BCC could only be detected for the relative abundance of *O*-glycan core types. Significance (*p* ≤ 0.04) is indicated by asterisk (*).

The BCC tissue *O*-glycan profile showed significant level changes for various structures (Table S7 in Supplementary Material). Core 1 type *O*-glycan levels were reduced while core 2 type *O*-glycans experienced upregulation from 38% in healthy tissue to 53% in BCC (Figure 5). As a consequence, the increase of core 2 type *O*-glycans affected the relative abundance of the NeuAc linkages. Levels of α2-3-NeuAc were increased while α2-6-NeuAc levels were reduced since the latter linkage was exclusively found on core 1 type *O*-glycans (including the most abundant structure ID: 5). Other structural features such as levels of total sialic acid, fucosylation or sulfate did not exhibit significant changes in BCC. Increase of core 2 type *O*-glycans in BCC was also confirmed in all of the 20 analyzed FFPE tissue sections (Figure S5 in Supplementary Material).

### Oligomannose and α2-3 Sialylated *N*-Glycans Are Affected in SCC

**Figure 6:**
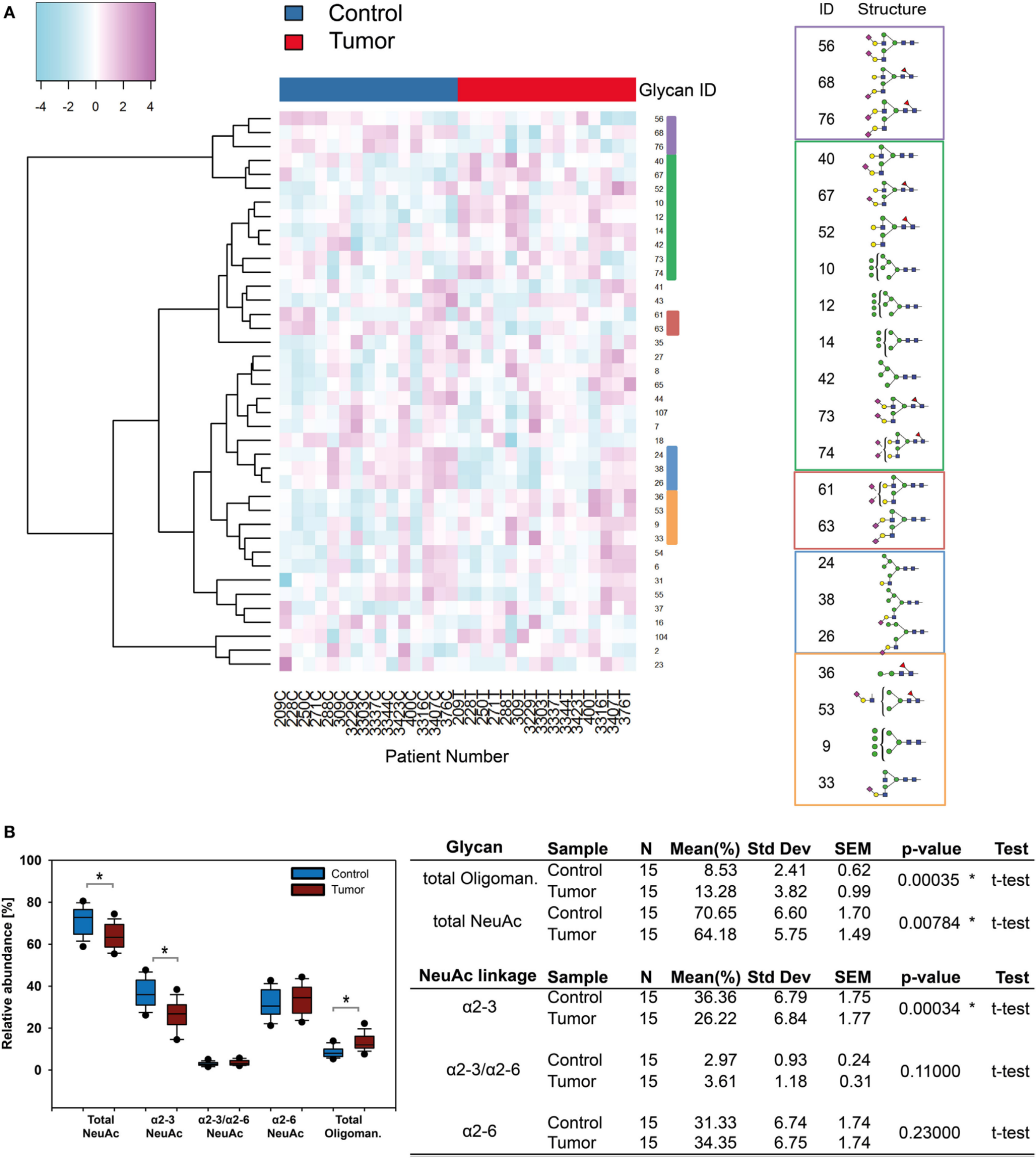
*N*-glycosylation changes in squamous cell carcinoma (SCC). **(A)** Supervised hierarchical cluster analysis of healthy and tumor skin. Rows display each of the 15 patient glycan data (T, tumor tissue; C, healthy control). Columns indicate the *N*-glycan ID. Five clusters can be observed: in healthy tissue *N*-glycans with α2-3 linked *N*-acetylneuraminic acid (NeuAc) appeared to be present in increased levels whereas α2-6-NeuAc and oligomannose *N*-glycan levels were higher in tumor tissue. **(B)** Statistical evaluation of sialylated and oligomannose *N*-glycans uncovered significant changes [*p* ≤ 0.04, using a *t*-test, indicated by an asterisk (*)]. Oligomannose *N*-glycans were upregulated whereas α2-3 linked NeuAc carrying *N*-glycans were down regulated in SCC.

The results obtained for the BCC-associated glycome changes encouraged us to apply our glycomics methods to FFPE histopathological tissue sections obtained from 15 SCC patients to compare possible protein glycosylation signature differences between the two cancer types. To identify potentially up or down regulated glycan structures we generated a heatmap based on the 40 most abundant *N*-glycans (Figure 6A). Clusters indicated structures experiencing level changes in SCC tumor tissue. In SCC oligomannose carrying *N*-glycans showed increased levels, while α2-3-NeuAc *N*-glycans were less abundant compared with healthy tissue. With an average 1.56-fold upregulation in tumor samples, oligomannose *N*-glycans Man9, Man8, Man7, and Man6 showed a similar trend in SCC as demonstrated for BCC. The total abundance of sialylated *N*-glycans was reduced from 70% in healthy tissue to 64% in SCC (Figure 6B). This effect was mainly due to a significant reduction of α2-3 sialylated *N*-glycans (from 36% down to 26%) while the levels of α2-6 sialylated *N*-glycans remained essentially unaffected.

### *O*-Glycan Alterations in FFPE Histopathological Tissues of SCC

**Figure 7:**
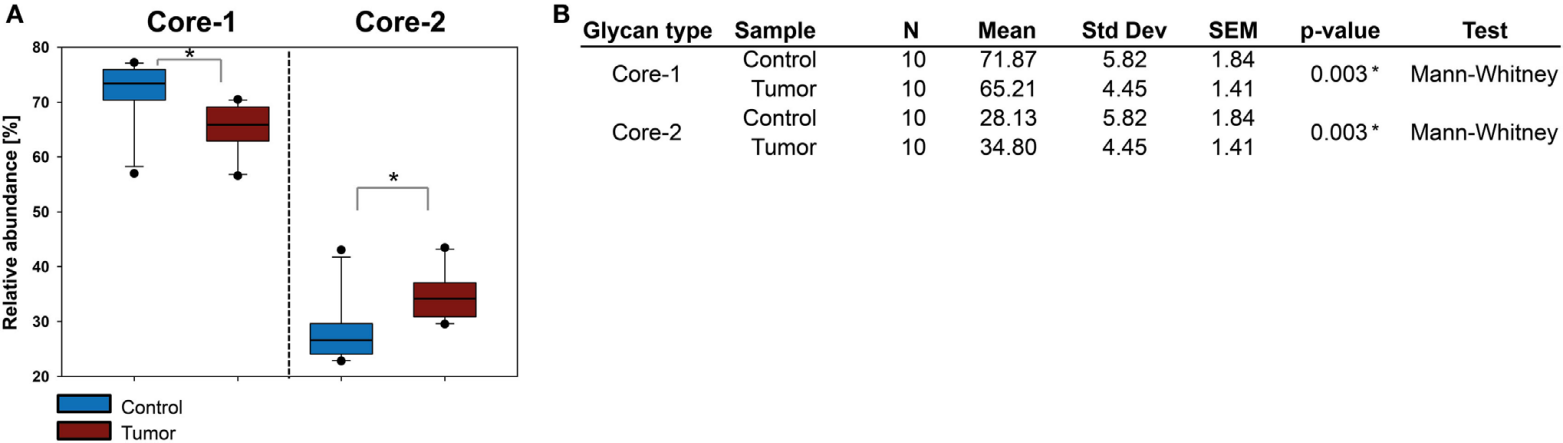
Evaluation of *O*-glycan core structures abundance in squamous cell carcinoma (SCC). **(A)** Comparison of relative abundances of core 1 and core 2 *O*-glycans in healthy and SCC tissue from 10 patients. Core 2 glycans were upregulated in SCC. In contrast to basal cell carcinoma, core 2 *O*-glycans did not exceed the amount of core 1 *O*-glycans. **(B)** Statistical evaluation of *O*-glycan cores. Significance (*p* ≤ 0.04) is indicated by an asterisk (*).

The SCC *O*-glycome could be determined for 10 patient samples revealing a trend similar to the one observed for BCC where core 2 type *O*-glycan levels were increased, albeit to a lesser extent. Core 1 type *O*-glycans remained the most abundant structures in both healthy and tumor tissue (Figure 7). Other structure features such as sialic acid linkage or fucosylation did not show any significant alterations.

### Glycoproteins Associated With the GO-Term “Binding” Are the Major Glycoproteins Identified in Skin Tissue

To gain insight into the glycoproteins carrying oligomannose structures as well as sialylated glycan structures, we performed a qualitative glycoproteomics analysis. Tryptic (glyco)peptides generated from the pooled protein extracts were split into two fractions: one non-enriched, but enzymatically deglycosylated fraction and one fraction containing glycopeptides enriched by HILIC. (Glyco)peptides were analyzed by reversed phase nano-LC–MS/MS, whereas glycopeptide data for the HILIC fraction were acquired under HCD-pd-CID mode.

**Figure 8:**
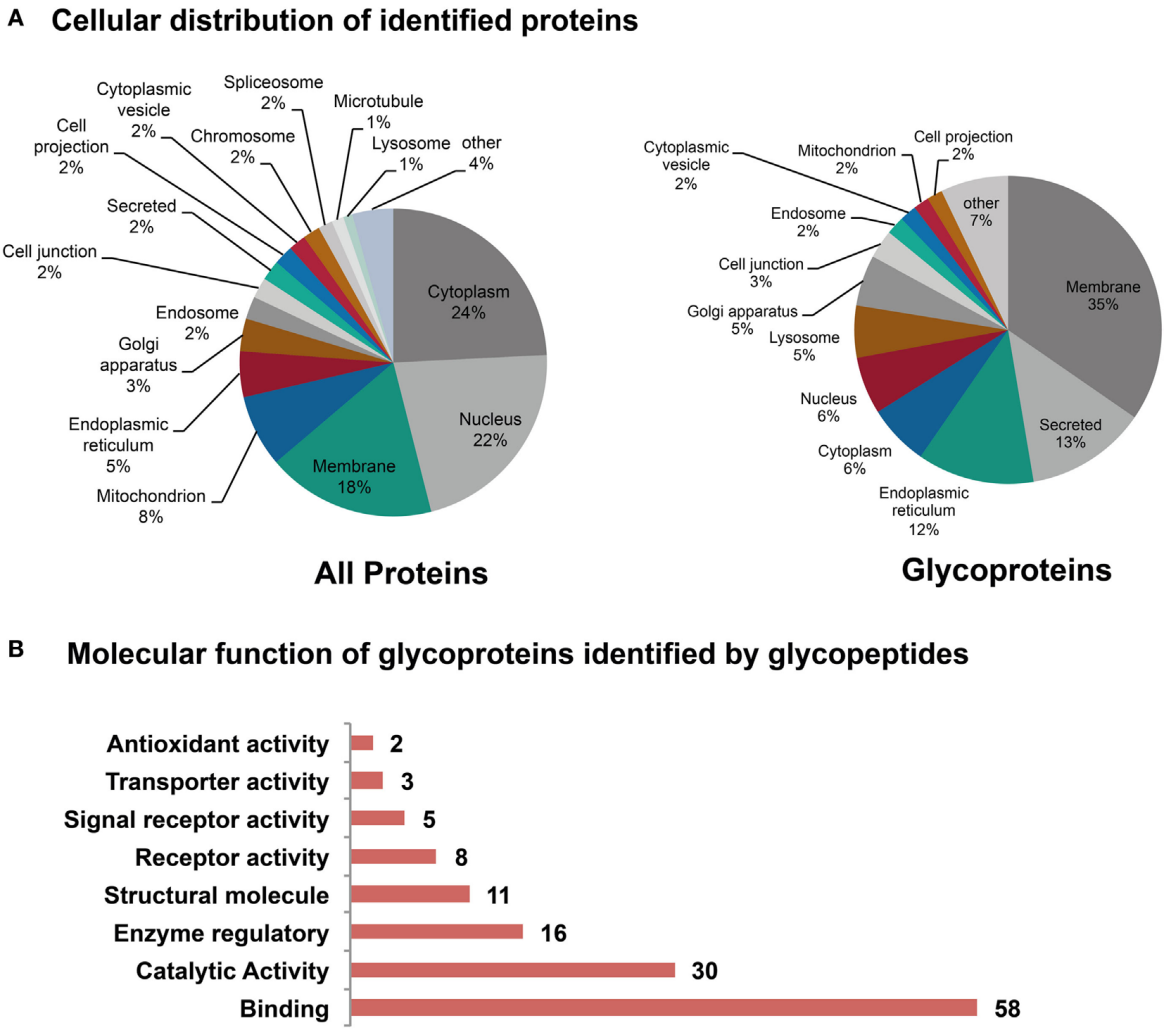
Subcellular location of the identified (glyco)proteins. **(A)** The majority of the identified proteins were predicted to be in the cytoplasm, the nucleus, and in membranes. From the 4,000 identified proteins, 407 were reported to be glycoproteins. **(B)** Molecular function of glycoproteins as determined form the identified glycopeptides. Summary has been established from 72 of the 78 identified glycoproteins. A large portion of the identified glycoproteins is involved in binding and catalytic activities.

In the non-enriched fraction, over 4,000 proteins were identified, of which 407 are listed as glycoproteins in the UniProt database. The majority of all identified proteins are located in the cytoplasm, nucleus, and in membranes, whereas the glycoproteins are particularly located in membranes, the ER or found secreted (Figure 8A). Glycopeptides were identified in the HILIC enriched fraction using Byonic software, and a library based on the 407 initially identified glycoproteins. Only glycopeptide hits exhibiting a score greater than 100 were considered, while glycopeptide spectra with a score below 100 were only considered after manual validation if other glycoforms of the same peptide were identified with a score above 100. Overall, glycopeptides from 78 glycoproteins were identified (Table S8 in Supplementary Material). The intention of this experiment was to identify glycoproteins including their site-specific glycosylation, but it was not possible to quantitatively distinguish between healthy and tumor tissue since the sample material was limited. The majority of the identified glycoproteins were involved in binding activities such as receptor binding, protein complex binding, or cell adhesion molecule binding (Figure 8B).

Most glycopeptides were identified from following proteins: decorin, IgG, complement C3, collagen, vitronectin, and microfibril-associated glycoprotein-4. Oligomannose *N*-glycans appeared to be the major structures on 23 of the identified glycoproteins. Among these were also dolichyl-diphosphooligosaccharide-protein-glycosyltransferase (DDOST) and *N*-acetylglucosamine-6-sulfatase (G6S), which are both involved in glycan processing. The most prominently detected oligomannosylated glycoproteins were: complement C3, laminin, serpin, peptidyl-prolyl-cis–trans isomerase, endoplasmin, and microfibril-associated glycoprotein-4 (Table 1). All of these proteins were detected by glycopeptides carrying oligomannose structures ranging from Man5 to Man9. Serpin and microfibril-associated glycoprotein-4, furthermore, exhibited complex structures. Serpin was observed with core fucose and one or two terminal GlcNAc residues, whereas microfibril-associated glycoprotein-4 was found to carry *N*-glycans with terminal galactose and singly sialylated glycans.

**Table 1:** Identified glycoproteins carrying oligomannose *N*-glycans.

Peptide <ProteinMetrics confidential>	Glycans NHFAGNa	Observed *m*/*z*	*z*	Observed (M + H)	Calc. mass (M + H)	Mass error (ppm)	Cleavage	Score
**Complement C3**	**P01024**							
K.TVLTPATN[+0.984]HM[+15.995]GN[+1,378.476]VTFTIPANR.E	HexNAc(2)Hex(6)	1,217.553	3	3,650.644	3,650.615	8.1	Specific	124.0
K.TVLTPATN[+0.984]HMGN[+1,378.476]VTFTIPANR.E	HexNAc(2)Hex(6)	1,212.217	3	3,634.635	3,634.620	4.2	Specific	379.1
K.TVLTPATN[+0.984]HMGN[+1,540.529]VTFTIPANR.E	HexNAc(2)Hex(7)	1,266.237	3	3,796.695	3,796.673	5.8	Specific	384.1
K.TVLTPATNHM[+15.995]GN[+1,216.423]VTFTIPANR.E	HexNAc(2)Hex(5)	1,163.202	3	3,487.590	3,487.578	3.6	Specific	141.1
K.TVLTPATNHM[+15.995]GN[+1,378.476]VTFTIPANR.E	HexNAc(2)Hex(6)	1,217.217	3	3,649.636	3,649.631	1.5	Specific	362.2
K.TVLTPATNHMGN[+1,378.476]VTFTIPANR.E	HexNAc(2)Hex(6)	909.165	4	3,633.637	3,633.636	0.3	Specific	555.7
K.TVLTPATNHMGN[+1,540.529]VTFTIPANR.E	HexNAc(2)Hex(7)	1,265.903	3	3,795.696	3,795.689	1.9	Specific	497.1
K.TVLTPATNHMGN[+1,864.634]VTFTIPANR.E	HexNAc(2)Hex(9)	1,374.273	3	4,120.805	4,119.794	1.8	Specific	107.6
K.VVPEGIRMN[+1,702.581]K.T	HexNAc(2)Hex(8)	949.077	3	2,845.216	2,845.216	0.0	Specific	142.1
R.MN[+1,702.581]KTVAVR.T	HexNAc(2)Hex(8)	874.373	3	2,621.105	2,621.100	1.7	Specific	328.3
R.MN[+1,864.634]KTVAVR.T	HexNAc(2)Hex(9)	928.391	3	2,783.159	2,783.153	2.2	Specific	299.3

**Laminin subunit gamma-1**	**P11047**							
R.RVN[+0.984]DN[+1,540.529]KTAAEEALR.K	HexNAc(2)Hex(7)	782.841	4	3,128.343	3,128.337	2.0	Specific	200.0
K.TAN[+1,540.529]DTSTEAYNLLLR.T	HexNAc(2)Hex(7)	1,074.797	3	3,222.377	3,222.368	3.0	Specific	249.2
K.TAN[+1,216.423]DTSTEAYNLLLR.T	HexNAc(2)Hex(5)	966.761	3	2,898.269	2,898.262	2.4	Specific	743.2
K.LLNN[+1,216.423]LTSIK.I	HexNAc(2)Hex(5)	1,116.524	2	2,232.040	2,232.038	1.3	Specific	98.8
K.LLNN[+1,378.476]LTSIK.I	HexNAc(2)Hex(6)	1,197.552	2	2,394.096	2,394.090	2.3	Specific	84.4
K.LLNN[+1,216.423]LTSIK.I	HexNAc(2)Hex(5)	1,116.524	2	2,232.041	2,232.038	1.4	Specific	110.9

**Serpin H1**	**P50454**							
R.N[+1,378.476]VTWK.L	HexNAc(2)Hex(6)	1,013.420	2	2,025.832	2,025.827	2.8	Specific	267.7
R.N[+1,444.534]VTWK.L	HexNAc(4)Hex(3)Fuc(1)	1,046.447	2	2,091.887	2,091.885	1.0	Specific	163.0
R.N[+1,540.529]VTWK.L	HexNAc(2)Hex(7)	1,094.447	2	2,187.886	2,187.880	2.8	Specific	244.1
R.N[+1,702.581]VTWK.L	HexNAc(2)Hex(8)	1,175.471	2	2,349.936	2,349.933	1.3	Specific	272.5
R.N[+1,864.634]VTWK.L	HexNAc(2)Hex(9)	1,256.498	2	2,511.989	2,511.985	1.6	Specific	216.2
R.SLSN[+1,241.454]STAR.N	HexNAc(3)Hex(3)Fuc(1)	1,038.946	2	2,076.886	2,076.881	2.0	Specific	125.8
R.SLSN[+1,378.476]STAR.N	HexNAc(2)Hex(6)	1,107.458	2	2,213.909	2,213.903	3.0	Specific	228.1

**Peptidyl-prolyl cis–trans isomerase**	**Q96AY3**							
R.YHYN[+1,378.476]C[+57.021]SLLDGTQLFTSHDYGAPQEATLGANK.V	HexNAc(2)Hex(6)	1,213.525	4	4,851.079	4,850.067	1.8	Specific	324.9
R.YHYN[+1,378.476]GTFEDGKK.F	HexNAc(2)Hex(6)	710.041	4	2,837.141	2,837.141	0.1	Specific	346.6
R.YHYN[+1,702.581]GSLMDGTLFDSSYSR.N	HexNAc(2)Hex(8)	1,305.853	3	3,915.543	3,915.542	0.3	Specific	365.9
R.YHYN[+1,702.581]GTLLDGTSFDTSYSK.G	HexNAc(2)Hex(8)	1,291.193	3	3,871.566	3,871.558	1.8	Specific	396.5
R.YHYN[+1,864.634]GSLMDGTLFDSSYSR.N	HexNAc(2)Hex(9)	1,359.874	3	4,077.607	4,077.595	3.1	Specific	355.7
R.YHYN[+1,864.634]GTLLDGTSFDTSYSK.G	HexNAc(2)Hex(9)	1,345.549	3	4,034.632	4,033.611	4.2	Specific	291.9

**Endoplasmin**	**P14625**							
K.HN[+0.984]N[+1,216.423]DTQHIWESDSNEFSVIADPR.G	HexNAc(2)Hex(5)	982.919	4	3,928.654	3,928.615	9.8	Specific	78.0
K.HN[+0.984]N[+1,378.476]DTQHIWESDSNEFSVIAD PR.G	HexNAc(2)Hex(6)	1,023.678	4	4,091.690	4,090.668	4.6	Specific	187.1
K.HNN[+1,378.476]DTQHIWESDSNEFSVIADPR.G	HexNAc(2)Hex(6)	1,023.178	4	4,089.691	4,089.684	1.9	Specific	340.4
K.HNN[+1,702.581]DTQHIWESDSNEFSVIADPR.G	HexNAc(2)Hex(8)	1,104.458	4	4,414.810	4,413.789	4.0	Specific	353.4

**Microfibril-associated glycoprotein-4**	**P55083**							
R.FN[+1,216.423]GSVSFFR.G	HexNAc(2)Hex(5)	1,138.977	2	2,276.946	2,276.944	0.9	Specific	243.7
R.FN[+1,378.476]GSVSFFR.G	HexNAc(2)Hex(6)	1,220.004	2	2,439.000	2,438.997	1.4	Specific	308.4
R.FN[+1,378.476]GSVSFFR.G	HexNAc(2)Hex(6)	1,220.005	2	2,439.003	2,438.997	2.5	Specific	178.0
R.FN[+1,540.529]GSVSFFR.G	HexNAc(2)Hex(7)	1,301.031	2	2,601.055	2,601.050	2.1	Specific	100.0
R.VDLEDFEN[+1,768.640]NTAYAK.Y	HexNAc(4)Hex(5)Fuc(1)	1,133.136	3	3,397.392	3,397.383	2.5	Specific	53.2
R.VDLEDFEN[+1,768.640]NTAYAK.Y	HexNAc(4)Hex(5)Fuc(1)	1,133.133	3	3,397.384	3,397.383	0.0	Specific	132.2
R.VDLEDFEN[+1,768.640]NTAYAK.Y	HexNAc(4)Hex(5)Fuc(1)	1,133.133	3	3,397.385	3,397.383	0.4	Specific	327.8
R.VDLEDFEN[+2,059.735]NTAYAK.Y	HexNAc(4)Hex(5)Fuc(1)NeuAc(1)	1,230.166	3	3,688.483	3,688.479	1.2	Specific	159.8

Among the 78 glycoproteins, 33 were found to be sialylated (Table 2). Glycoproteins most commonly detected with core fucose, and one and two terminal sialic acids were again decorin and two isoforms of collagen VI. While sialylated *N*-glycans from collagen were only detected together with core fucosylation, decorin was also found to be singly sialylated without core fucosylation. The most frequently detected, non-fucosylated sialylated glycoproteins were vitronectin, alpha-1-antitrypsin, torsin-1A-interacting-protein-1, and metalloreductase STEAP3 (Table 3).

**Table 2:** Identified glycoproteins carrying sialylated and core-fucosylated *N*-glycans.

Peptide <ProteinMetrics confidential>	Glycans NHFAGNa	Observed *m*/*z*	*z*	Observed (M + H)	Calc. mass (M + H)	Mass error (ppm)	Cleavage	Score
**Decorin**	**P07585**							
R.IADTN[+1,913.677]ITSIPQGLPPSLTELHLDGNK.I	HexNAc(4)Hex(5)NeuAc(1)	1,165.290	4	4,658.137	4,658.124	2.9	Specific	126.1
K.LGLSFNSISAVDN[+2,350.830]GSLANTPHLR.E	HexNAc(4)Hex(5)Fuc(1)NeuAc(2)	1,184.525	4	4,735.077	4,734.067	1.4	Specific	113.9
K.LGLSFNSISAVDN[+2,059.735]GSLANTPHLR.E	HexNAc(4)Hex(5)Fuc(1)NeuAc(1)	1,111.751	4	4,443.981	4,442.971	1.3	Specific	396.3
K.LGLSFNSISAVDN[+1,768.640]GSLANTPHLR.E	HexNAc(4)Hex(5)Fuc(1)	1,038.726	4	4,151.884	4,151.876	1.8	Specific	165.8
K.LGLSFN[+0.984]SISAVDN[+2,059.735]GSLANTPHL R.E	HexNAc(4)Hex(5)Fuc(1)NeuAc(1)	1,111.744	4	4,443.955	4,443.955	0.0	Specific	244.7
R.IADTN[+1,419.502]ITSIPQGLPPSLTELHLDGNK.I	HexNAc(3)Hex(5)	1,041.743	4	4,163.950	4,163.949	0.4	Specific	266.0
R.IADTN[+1,257.449]ITSIPQGLPPSLTELHLDGNK.I	HexNAc(3)Hex(4)	1,001.231	4	4,001.902	4,001.896	1.5	Specific	296.2
R.IADTN[+1,622.582]ITSIPQGLPPSLTELHLDGNK.I	HexNAc(4)Hex(5)	1,092.514	4	4,367.032	4,367.028	1.0	Specific	396.0
K.LGLSFNSISAVDN[+2,059.735]GSLANTPHLR.E	HexNAc(4)Hex(5)Fuc(1)NeuAc(1)	1,111.501	4	4,442.983	4,442.971	2.6	Specific	292.4
K.LGLSFNSISAVDN[+1,768.640]GSLANTPHLR.E	HexNAc(4)Hex(5)Fuc(1)	1,038.726	4	4,151.884	4,151.876	1.8	Specific	493.3
K.LGLSFN[+0.984]SISAVDN[+1,768.640]GSLANTPHL R.E	HexNAc(4)Hex(5)Fuc(1)	1,038.972	4	4,152.866	4,152.860	1.4	Specific	517.4

**Collagen alpha-2(VI)**	**P12110**							
R.GTFTDC[+57.021]ALAN[+1,622.582]MTEQIR.Q	HexNAc(4)Hex(5)	1,150.813	3	3,450.426	3,450.418	2.2	Specific	191.3
R.N[+1,768.640]FTAADWGQSR.D	HexNAc(4)Hex(5)Fuc(1)	1,007.742	3	3,021.212	3,021.210	0.8	Specific	316.8
R.N[+1,768.640]M[+15.995]TLFSDLVAEK.F	HexNAc(4)Hex(5)Fuc(1)	1,051.448	3	3,152.328	3,152.322	1.9	Specific	102.2
R.N[+1,768.640]MTLFSDLVAEK.F	HexNAc(4)Hex(5)Fuc(1)	1,046.116	3	3,136.334	3,136.327	2.4	Specific	225.4
R.N[+2,059.735]FTAADWGQSR.D	HexNAc(4)Hex(5)Fuc(1)NeuAc(1)	1,104.773	3	3,312.306	3,312.305	0.1	Specific	157.1
R.N[+2,059.735]M[+15.995]TLFSDLVAEK.F	HexNAc(4)Hex(5)Fuc(1)NeuAc(1)	114 8.478	3	3,443.419	3,443.417	0.4	Specific	122.5
R.N[+2,059.735]MTLFSDLVAEK.F	HexNAc(4)Hex(5)Fuc(1)NeuAc(1)	114 3.148	3	3,427.430	3,427.422	2.3	Specific	154.5
R.N[+2,350.830]FTAADWGQSR.D	HexNAc(4)Hex(5)Fuc(1)NeuAc(2)	1,201.806	3	3,603.404	3,603.401	0.9	Specific	109.0
R.N[+2,350.830]M[+15.995]TLFSDLVAEK.F	HexNAc(4)Hex(5)Fuc(1)NeuAc(2)	1,245.512	3	3,734.521	3,734.513	2.1	Specific	128.6
R.RN[+892.317]FTAADWGQSR.D	HexNAc(2)Hex(3)	767.669	3	2,300.993	2,300.989	1.7	Specific	30.0

**Isoform 2 of collagen alpha-3(VI)**	**P12111-2**							
R.QLINALQIN[+1,768.640]NTAVGHALVLPAGR.D	HexNAc(4)Hex(5)Fuc(1)	1,038.756	4	4,152.002	4,151.996	1.3	Specific	590.4
R.QLINALQIN[+1,768.640]NTAVGHALVLPAGR.D	HexNAc(4)Hex(5)Fuc(1)	1,384.674	3	4,152.007	4,151.996	2.5	Specific	204.3
R.QLINALQIN[+2,059.735]NTAVGHALVLPAGR.D	HexNAc(4)Hex(5)Fuc(1)NeuAc(1)	1,111.528	4	4,443.091	4,443.092	-0.2	Specific	312.9
R.QLINALQIN[+2,350.830]NTAVGHALVLPAGR.D	HexNAc(4)Hex(5)Fuc(1)NeuAc(2)	1,184.303	4	4,734.190	4,734.187	0.6	Specific	249.2
R.QLINALQIN[+2,372.812]N[+0.984]TAVGHALVLPAG R.D	HexNAc(4)Hex(5)Fuc(1)NeuAc(2)N	952.437	5	4,758.155	4,757.153	-0.2	Specific	110.6
R.QLINALQIN[+349.137]NTAVGHALVLPAGR.D	HexNAc(1)Fuc(1)	911.504	3	2,732.499	2,732.494	1.7	Specific	400.8

**Table 3:** Identified glycoproteins carrying sialylated *N*-glycans without any detectable core fucosylation.

Peptide <ProteinMetrics confidential>	Glycans NHFAGNa	Observed *m*/*z*	*z*	Observed (M + H)	Calc. mass (M + H)	Mass error (ppm)	Cleavage	Score
**Vitronectin**	**P04004**							
R.N[+2,204.772]ISDGFDGIPDNVDAALALPAHSYSGR.E	HexNAc(4)Hex(5)NeuAc(2)	1,245.033	4	4,977.110	4,977.095	3.0	Specific	253.8
K.NN[+2,204.772]ATVHEQVGGPSLTSDLQAQSK.G	HexNAc(4)Hex(5)NeuAc(2)	1,147.492	4	4,586.948	4,585.942	0.6	Specific	132.6
K.N[+2,204.772]GSLFAFR.G	HexNAc(4)Hex(5)NeuAc(2)	1,039.423	3	3,116.253	3,116.246	2.4	Specific	147.7
R.N[+2,204.772]ISDGFDGIPDNVDAALALPAHSYSGR.E	HexNAc(4)Hex(5)NeuAc(2)	830.524	6	4,978.108	4,977.095	1.9	Specific	353.2
K.N[+1,419.502]GSLFAFR.G	HexNAc(3)Hex(5)	1,165.992	2	2,330.978	2,330.976	0.9	Specific	105.9
R.N[+1,622.582]ISDGFDGIPDNVDAALALPAHSYSGR.E	HexNAc(4)Hex(5)	1,099.733	4	4,395.911	4,394.904	0.9	Specific	131.3
K.N[+1,913.677]GSLFAFR.G	HexNAc(4)Hex(5)NeuAc(1)	942.390	3	2,825.155	2,825.150	1.7	Specific	53.6
K.N[+1,622.582]GSLFAFR.G	HexNAc(4)Hex(5)	845.356	3	2,534.054	2,534.055	-0.4	Specific	147.9
R.N[+1,622.582]ISDGFDGIPDNVDAALALPAHSYSGR.E	HexNAc(4)Hex(5)	1,099.484	4	4,394.913	4,394.904	2.0	Specific	292.9

**Alpha-1-antitrypsin**	**P01009**							
K.YLGN[+2,204.772]ATAIFFLPDEGK.L	HexNAc(4)Hex(5)NeuAc(2)	990.924	4	3,960.674	3,960.668	1.6	Specific	485.1
R.QLAHQSN[+1,622.582]STNIFFSPVSIATAFAMLSLG TK.A	HexNAc(4)Hex(5)	1,202.063	4	4,805.229	4,804.217	1.9	Specific	275.4
R.QLAHQSN[+1,622.582]STNIFFSPVSIATAFAM[+15.995]LSLGTK.A	HexNAc(4)Hex(5)	1,206.063	4	4,821.228	4,820.212	2.8	Specific	179.1
K.YLGN[+1,913.677]ATAIFFLPDEGK.L	HexNAc(4)Hex(5)NeuAc(1)	1,223.866	3	3,669.583	3,669.572	2.9	Specific	509.1
K.YLGN[+1,622.582]ATAIFFLPDEGK.L	HexNAc(4)Hex(5)	1,126.831	3	3,378.479	3,378.477	0.7	Specific	626.8

**Torsin-1A-interacting protein**	**Q5JTV8**							
R.SQTFLEKHLN[+1,702.581]SSHPR.S	HexNAc(2)Hex(8)	697.504	5	3,483.492	3,483.490	0.4	Specific	356.2
R.SQTFLEKHLN[+1,913.677]SSHPR.S	HexNAc(4)Hex(5)NeuAc(1)	1,232.204	3	3,694.599	3,694.586	3.4	Specific	205.4
R.SQTFLEKHLN[+2,204.772]SSHPR.S	HexNAc(4)Hex(5)NeuAc(2)	997.179	4	3,985.696	3,985.681	3.5	Specific	185.1

**Metalloreductase STEAP3**	**Q658P3**							
K.QVLAN[+1,913.677]KSHLWVEEEVWR.M	HexNAc(4)Hex(5)NeuAc(1)	1,009.951	4	4,036.780	4,036.780	0.0	Specific	196.8
K.QVLAN[+2,204.772]KSHLWVEEEVWR.M	HexNAc(4)Hex(5)NeuAc(2)	866.384	5	4,327.891	4,327.876	3.6	Specific	139.2

## Discussion

Little is known about the human skin glycome as detailed glycomic and glycoproteomic investigations have not been undertaken. Skin cancer studies were based on cell culture material (34, 35), limiting the value of information that can be obtained from these studies as the skin glycoproteome is likely to be highly dependent on the developmental stage, cell environment and the organism. Skin consists of various layers with different organism-specific properties and a complex extracellular matrix network. Outcomes of glycomics studies in an artificial environment as present in cell culture might result in detection of culture condition artifacts that deviate from the actual *in vivo* glycome. We therefore focused on tissue biopsies to investigate disease-associated glycoproteome signatures.

More than 82% of the *N*-glycans found in human skin carried at least one or more NeuAc residue. In skin tissue, α2-3 and α2-6-NeuAc linkage to *N*-glycans occurs in almost equal levels. The high occurrence of sialic acid containing *N*-glycans was particularly interesting since an earlier study (36) using a hydrazine-capture MALDI-TOF-MS approach reported mainly neutral and singly sialylated (~12% epidermis/~35% dermis) species as well as lower amounts of doubly sialylated *N*-glycans (~5% epidermis/~20% dermis) to be present in human skin. Some of the sialic acids could have been lost during the various preparation steps. Uematsu and coworkers separated dermis and epidermis by heat treatment, which could also have contributed to the reduced global sialylation levels. Since our results clearly indicated that the global *N-*glycome was overall very comparable between the individual patient samples, interperson variations are an unlikely explanation for the sialylation level differences.

Cancer-associated glycosylation differences were generally observed for both, BCC and SCC. The extent of observed *N*-glycan level changes showed some patient-specific variations and could not be detected in six FFPE sample sets. Such variation is expected for complex biological samples as numerous individual factors can influence the outcome of such studies. Healthy skin biopsies were taken in direct proximity to the tumor tissue. MALDI imaging studies have revealed that tumor-associated molecular differences can in some cases extend in a decreasing manner up to 1.5 cm into the surrounding healthy tissue (37). Thus, the observed *N*-glycosylation changes could be even more prominent if compared with more distal tissue.

The observed *N*-glycan level differences were more prominent in the biopsies than in FFPE prepared samples. This could be due to the overall lower sample amount that was obtained from FFPE histopathological slides and the resulting general lower glycan yield. Moreover, the frozen biopsies were specifically prepared for this study by “punch excision” after removal from the patients’ skin whereas the FFPE tissue was prepared using a routine dermato-pathological workup possibly containing more surrounding tissue. The different sampling methods for the tissue biopsies and the FFPE tissue slides could have influenced the ratio and amount of tumor and surrounding tissue. Despite the fact that overall the same pattern of *N*-glycan level changes was observed in FFPE tissue, it could account for the fact that these differences were less profound in the FFPE sample set.

Altered glycosylation is a hallmark of cancer (38), which raises the questions whether such alterations are simply side effects of the malignant transformation, if cancer cells actively benefit from this development or even both. Answers to these questions are likely to be specific for each cancer type. In this study, oligomannose type *N*-glycan levels were found to be increased in BCC and SCC both being epithelial cancers. The same pattern was also reported to occur in breast cancer patient (39) as well as in mouse sera of implanted head and neck tumors which also arise from epithelium (40). Recently published mass spectrometry imaging studies reported an increase of the Man8 *N*-glycan in tumor tissue from ovarian cancer (41) and hepatocellular carcinoma (42). In the ER oligomannose *N*-glycans are crucial for *N*-glycan processing and protein folding. Increased cell division and hence protein expression in cancer tissue can lead to incomplete *N*-glycan processing manifesting itself in incomplete trimming of oligomannose structures. As glycosylation is protein- and site-specific (43) changes from complex to oligomannose structures can significantly impact the stability, binding affinities or functions of the respective glycoprotein (44, 45). Oligomannose *N*-glycans are also known ligands for C-type lectins such as DC-SIGN and langerin (46). Both are found on antigen presenting immune cells (47). Thus, an increase in oligomannose structures would suggest an upregulation of immune responses; however, studies have shown that dendritic cells in non melanoma skin cancer seem to be impaired in their ability to stimulate T-cell proliferation (48, 49). This condition could then allow an increased presence of these structures without risking augmented immune attention.

Our glycoproteomics study identified the presence of oligomannose *N*-glycans on several extracellular matrix-associated glycoproteins such as laminin, serpin, and microfibril-associated glycoprotein-4. Oligomannose *N*-glycans on extracellular matrix glycoprotein are well known to participate in cell adhesion processes and can represent ligands for monocyte adhesion in endothelial cells, which associates these with inflammation processes at the tumor site (50). While upregulation of immune responses would rather point toward a reaction in response to tumor progression, cancer-associated inflammation could be beneficial for the tumor by driving its progression (51).

In agreement with literature, we found complement C3 glycosylated with oligomannose structures (52). Complement C3 usually plays a crucial role in the immune system’s defense mechanisms against malignant cells and pathogens by initiating the membrane attack complex (53). By contrast, several studies have also demonstrated a tumor promoting role of complements. Complement activation may drive chronic inflammation, promote an immunosuppressive microenvironment, induce angiogenesis, and activate cancer-related signaling pathways (54). Recently, significant elevated mRNA levels of complement C3 have been reported to be associated with the progression of SCC (55). Among others complement activation can be triggered by oligomannose *N*-glycans *via* the mannan binding pathway (56). Therefore, it might be conceivable that tumor-associated increased oligomannose *N*-glycan levels could derive from an increased expression of oligomannose carrying glycoproteins and the increased presence of complement C3.

Both BCC and SCC show increased core 2 *O*-glycan levels. Upregulation of core 2 *O*-glycans can originate from increased core 2 β-1,6-*N*-acetylglucosaminyltransferase (C2GnT) expression, leading to an augmented conversion of core 1 to core 2 *O*-glycans. Reducing the amount of core 1 and increasing core 2 *O*-glycans changes options for subsequent modifications potentially reducing molecular and cellular interactions while giving rise to others. Increased C2GnT expression has also been described for colorectal and lung carcinomas also correlating with increased occurrence of Lewis X structures (14, 57, 58). Another study reported upregulation of C2GnT and increased core 2 glycosylation in close relation to bladder cancer progression and the cancer cells ability to evade NK immunity (14, 59).

In our study differences in the sialome were only detected in SCC. A previous study compared the abundance of sialic acid linkages between SCC and BCC by lectin histochemistry and reported increased α2-6-NeuAc levels in SCC (60). As both α2-6 and α2-3-sialyltransferases share common substrates, α2-6 sialyltransferase overexpression can result in decreased α2-3-sialylation. We detected reduction of α2-3-NeuAc in SCC only accompanied by a non-significant increase of α2-6-NeuAc. Our data point toward an α2-3 sialyltransferase down regulation as the causative effect behind the change in NeuAc linkage levels. Differences in the two studies might originate from the fact that lectin assays consider not only *N*- and *O*-glycans but the sum of all glycan bearing structures such as glycolipids and glycosaminoglycans. Lectin assays are a powerful tool and commonly utilized but have to be carefully evaluated as lectines often have binding preferences to not only one but several glycan structures (61–63).

As changes in *N*-glycan-associated α2-3-NeuAc levels did not have any detectable effect on *O*-glycosylation it seems likely that the observed changes only affect specific proteins. Our *N*-glycome data revealed a link between α2-3-NeuAc linkage and core fucosylation. Interpolating these findings to the glycoproteomics data it appears that in the skin extracellular matrix-associated proteins like collagen, decorin, vitronectin are more likely to carry α2-3-NeuAc modified *N-*glycans. The observed reduction of α2-3-linked NeuAc could thus have a considerable impact on the functionality of these proteins and thus on the entire intercellular communication of tumor cells.

There is a long history in studying sialylation (16) and associated transferases (64) in the context of cancer. Being the terminating monosaccharides they are prominently exposed on the most outer part of the glycan and vital for numerous specific interaction events with lectins such as siglecs (sialic acid binding immunoglobulin-like lectins) or selectins (65). Many of these interactions seem to be related to immunoevasion pathways by inhibiting activation of immune cells (66, 67) or promote metastasis progression (68). Furthermore, increase in sialic acid levels also results in increase of negative charges on the cell surface, which is believed to decrease cell adhesiveness thus driving metastasis (69). Interestingly, cell culture studies of the more aggressive malignant melanoma reported increase in sialylated β1-6-branched *N*-glycans (70) as well as increased α2-3 linked NeuAc levels correlating with aggressiveness (71). With BCC and SCC being less aggressive and metastatic than melanoma the absent overexpression of α2-3-linked NeuAc and β1-6 branches in our study hence, could reflect some of the potential factors accounting for their lower aggressiveness. Many changes in glycosylation have been reported focusing on specific glycoproteins. Global glycomics might be unable to pick up such protein-specific changes, in particular if just subtle changes are present that affect specific glycoproteins. Future studies investigating protein and protein site-specific glycosylation patterns will be vital in uncovering the role such altered glycosylation signatures have on cancer onset and progression, which will inevitably lead to the identification of novel diagnostic, prognostic and treatment strategies.

## Conclusion

Our detailed study of the skin *N*- and *O*-glycome provided the basis for investigations of cancer-associated *N*-glycan changes. Oligomannose *N*-glycan levels were elevated in BCC and SCC. Expression of α2-3 sialylated *N*-glycan decreased in SCC while core 2 *O*-glycans increased in both BCC and SCC. Several glycoproteins were identified that might potentially be affected by the *N*-glycosylation changes. The exact biological causes for the observed upregulation together with their involvement in disease onset and progression cannot be explained yet. Our findings represent, however, a basis for studies toward a better understanding of the various molecular aspects of non-melanoma skin cancers.

## Ethics Statement

The study was approved by the institutional review board of the medical faculty at the University of Leipzig (No. 127-11-18042011).

## Author Contributions

UM, DK, PS, JS, and SG planned the project. UM and DK wrote the manuscript. JS, SG, and HV provided all samples. UM performed sample preparation and glycan analysis. UM, C-WK, and K-HK performed the glycopeptide analysis and sample preparation. RH and ER performed xCGE-LIF analysis. UM and FS performed statistical analyses. All the authors contributed during the review process with their individual input and ideas.

## Conflict of Interest Statement

Authors RH and ER are co-affiliated to company glyXera. All other authors declare no competing interests.

## Footnotes

^1^https://CRAN.R-project.org/package=gplots.

^2^https://CRAN.R-project.org/package=pROC.

## Abbreviations

ACN, acetonitrile; BCC, basal cell carcinoma; ESI, electrospray ionization; EtOH, ethanol; FA, formic acid; FFPE, formalin-fixed paraffin-embedded; Fuc, fucose; HILIC, hydrophilic interaction chromatography; LC, liquid chromatography; Man, mannose; MS, mass spectrometry; MS/MS, tandem mass spectrometry; NeuAc, *N*-acetylneuraminic acid; PGC, porous graphitized carbon; SCC, squamous cell carcinoma; SDS, sodium dodecyl sulfate; TFA, trifluoroacetic acid; xCGE-LIF, multiplexed capillary gel electrophoresis with laser induced fluorescence detection; ZIC-HILIC, zwitterionic hydrophilic interaction liquid chromatography.

## References

[1] Young EA Kornstein SG Marcus SM Harvey AT Warden D Wisniewski SR Sex differences in response to citalopram: a STAR*D report. J Psychiatr Res (2009) 43:503–11. 10.1016/j.jpsychires.2008.07.002 18752809 PMC2681489

[2] Narayanan DL Saladi RN Fox JL Ultraviolet radiation and skin cancer. Int J Dermatol (2010) 49:978–86. 10.1111/j.1365-4632.2010.04474.x 20883261

[3] Leiter U Eigentler T Garbe C. Epidemiology of skin cancer. Adv Exp Med Biol (2014) 810:120–40. 25207363 10.1007/978-1-4939-0437-2_7

[4] Rogers HW Weinstock MA Feldman SR Coldiron BM. Incidence estimate of nonmelanoma skin cancer (keratinocyte carcinomas) in the U.S. population, 2012. JAMA Dermatol (2015) 151:1081–6. 10.1001/jamadermatol.2015.1187 25928283

[5] Christiansen MN Chik J Lee L Anugraham M Abrahams JL Packer NH. Cell surface protein glycosylation in cancer. Proteomics (2014) 14:525–46. 10.1002/pmic.201300387 24339177

[6] Pinho SS Reis CA. Glycosylation in cancer: mechanisms and clinical implications. Nat Rev Cancer (2015) 15:540–55. 10.1038/nrc3982 26289314

[7] Smith AE Helenius A. How viruses enter animal cells. Science (2004) 304:237–42. 10.1126/science.1094823 15073366

[8] Kolarich D Lepenies B Seeberger PH. Glycomics, glycoproteomics and the immune system. Curr Opin Chem Biol (2012) 16:214–20. 10.1016/j.cbpa.2011.12.006 22221852

[9] Song EY Kang SK Lee YC Park YG Chung TH Kwon DH Expression of bisecting N-acetylglucosaminyltransferase-III in human hepatocarcinoma tissues, fetal liver tissues, and hepatoma cell lines of Hep3B and HepG2. Cancer Invest (2001) 19:799–807. 10.1081/CNV-100107741 11768033

[10] Nakagoe T Fukushima K Nanashima A Sawai T Tsuji T Jibiki M Expression of Lewis(a), sialyl Lewis(a), Lewis(x) and sialyl Lewis(x) antigens as prognostic factors in patients with colorectal cancer. Can J Gastroenterol (2000) 14:753–60. 10.1155/2000/149851 11064310

[11] Osumi D Takahashi M Miyoshi E Yokoe S Lee SH Noda K Core fucosylation of E-cadherin enhances cell-cell adhesion in human colon carcinoma WiDr cells. Cancer Sci (2009) 100:888–95. 10.1111/j.1349-7006.2009.01125.x 19302290 PMC11159289

[12] Fuster MM Esko JD. The sweet and sour of cancer: glycans as novel therapeutic targets. Nat Rev Cancer (2005) 5:526–42. 10.1038/nrc1649 16069816

[13] Pillai S Netravali IA Cariappa A Mattoo H Siglecs and immune regulation. Annu Rev Immunol (2012) 30:357–92. 10.1146/annurev-immunol-020711-075018 22224769 PMC3781015

[14] Hauselmann I Borsig L. Altered tumor-cell glycosylation promotes metastasis. Front Oncol (2014) 4:28. 10.3389/fonc.2014.00028 24592356 PMC3923139

[15] Varki A Cummings RD Esko JD Freeze HH Stanley P Bertozzi CR Essentials of Glycobiology. 2nd ed New York: Cold Spring Harbor Laboratory Press (2009). 20301239

[16] Bull C Stoel MA Den Brok MH Adema GJ. Sialic acids sweeten a tumor’s life. Cancer Res (2014) 74:3199–204. 10.1158/0008-5472.CAN-14-0728 24830719

[17] Ogawa H Ghazizadeh M Araki T. Tn and sialyl-Tn antigens as potential prognostic markers in human ovarian carcinoma. Gynecol Obstet Invest (1996) 41:278–83. 10.1159/000292284 8793500

[18] Imada T Rino Y Hatori S Takahashi M Amano T Kondo J Sialyl Tn antigen expression is associated with the prognosis of patients with advanced colorectal cancer. Hepatogastroenterology (1999) 46:208–14. 10228794

[19] Dotan N Altstock RT Schwarz M Dukler A. Anti-glycan antibodies as biomarkers for diagnosis and prognosis. Lupus (2006) 15:442–50. 10.1191/0961203306lu2331oa 16898180

[20] Almeida A Kolarich D. The promise of protein glycosylation for personalised medicine. Biochim Biophys Acta (2016) 1860:1583–95. 10.1016/j.bbagen.2016.03.012 26987810

[21] Hennig R Rapp E Kottler R Cajic S Borowiak M Reichl U. N-glycosylation fingerprinting of viral glycoproteins by xCGE-LIF. Methods Mol Biol (2015) 1331:123–43. 10.1007/978-1-4939-2874-3_8 26169738

[22] Hinneburg H Hofmann J Struwe WB Thader A Altmann F Varon Silva D Distinguishing N-acetylneuraminic acid linkage isomers on glycopeptides by ion mobility-mass spectrometry. Chem Commun (Camb) (2016) 52:4381–4. 10.1039/c6cc01114d 26926577

[23] Jensen PH Karlsson NG Kolarich D Packer NH. Structural analysis of N- and O-glycans released from glycoproteins. Nat Protoc (2012) 7:1299–310. 10.1038/nprot.2012.063 22678433

[24] Kolarich D Rapp E Struwe WB Haslam SM Zaia J Mcbride R The minimum information required for a glycomics experiment (MIRAGE) project: improving the standards for reporting mass-spectrometry-based glycoanalytic data. Mol Cell Proteomics (2013) 12:991–5. 10.1074/mcp.O112.026492 23378518 PMC3617344

[25] York WS Agravat S Aoki-Kinoshita KF Mcbride R Campbell MP Costello CE MIRAGE: the minimum information required for a glycomics experiment. Glycobiology (2014) 24:402–6. 10.1093/glycob/cwu018 24653214 PMC3976285

[26] Hennig R Cajic S Borowiak M Hoffmann M Kottler R Reichl U Towards personalized diagnostics via longitudinal study of the human plasma N-glycome. Biochim Biophys Acta (2016) 1860:1728–38. 10.1016/j.bbagen.2016.03.035 27038647

[27] Thiesler CT Cajic S Hoffmann D Thiel C Van Diepen L Hennig R Glycomic characterization of induced pluripotent stem cells derived from a patient suffering from phosphomannomutase 2 congenital disorder of glycosylation (PMM2-CDG). Mol Cell Proteomics (2016) 15:1435–52. 10.1074/mcp.M115.054122 26785728 PMC4824866

[28] Everest-Dass AV Jin D Thaysen-Andersen M Nevalainen H Kolarich D Packer NH. Comparative structural analysis of the glycosylation of salivary and buccal cell proteins: innate protection against infection by *Candida albicans*. Glycobiology (2012) 22:1465–79. 10.1093/glycob/cws112 22833316

[29] Anugraham M Jacob F Nixdorf S Everest-Dass AV Heinzelmann-Schwarz V Packer NH. Specific glycosylation of membrane proteins in epithelial ovarian cancer cell lines: glycan structures reflect gene expression and DNA methylation status. Mol Cell Proteomics (2014) 13:2213–32. 10.1074/mcp.M113.037085 24855066 PMC4159645

[30] Konze SA Cajic S Oberbeck A Hennig R Pich A Rapp E Quantitative assessment of sialo-glycoproteins and N-glycans during cardiomyogenic differentiation of human induced pluripotent stem cells. Chembiochem (2017) 18:1317–31. 10.1002/cbic.201700100 28509371

[31] Kolarich D Windwarder M Alagesan K Altmann F. Isomer-specific analysis of released N-glycans by LC-ESI MS/MS with porous graphitized carbon. Methods Mol Biol (2015) 1321:427–35. 10.1007/978-1-4939-2760-9_29 26082239

[32] Hinneburg H Korać P Schirmeister F Gasparov S Seeberger PH Zoldoš V Unlocking cancer glycomes from histopathological formalin-fixed and paraffin-embedded (FFPE) tissue microdissections. Mol Cell Proteomics (2017) 16:524–36. 10.1074/mcp.M116.062414 28122943 PMC5383776

[33] Ruhaak LR Hennig R Huhn C Borowiak M Dolhain RJ Deelder AM Optimized workflow for preparation of APTS-labeled N-glycans allowing high-throughput analysis of human plasma glycomes using 48-channel multiplexed CGE-LIF. J Proteome Res (2010) 9:6655–64. 10.1021/pr100802f 20886907

[34] Ciolczyk-Wierzbicka D Amoresano A Casbarra A Hoja-Lukowicz D Litynska A Laidler P. The structure of the oligosaccharides of N-cadherin from human melanoma cell lines. Glycoconj J (2004) 20:483–92. 10.1023/B:GLYC.0000038294.72088.b0 15316281

[35] Przybylo M Pochec E Link-Lenczowski P Litynska A. Beta1-6 branching of cell surface glycoproteins may contribute to uveal melanoma progression by up-regulating cell motility. Mol Vis (2008) 14:625–36. 18385798 PMC2276181

[36] Uematsu R Shinohara Y Nakagawa H Kurogochi M Furukawa J Miura Y Glycosylation specific for adhesion molecules in epidermis and its receptor revealed by glycoform-focused reverse genomics. Mol Cell Proteomics (2009) 8:232–44. 10.1074/mcp.M800145-MCP200 18824476

[37] Schwamborn K Caprioli RM Molecular imaging by mass spectrometry – looking beyond classical histology. Nat Rev Cancer (2010) 10:639–46. 10.1038/nrc2917 20720571

[38] Varki A Kannagi R Toole B Stanley P Glycosylation changes in cancer. 3rd ed In: Varki A Cummings RD Esko JD Stanley P Hart GW Aebi M, editors. Essentials of Glycobiology. Harbor, NY: Cold Spring (2017).

[39] de Leoz ML Young LJ An HJ Kronewitter SR Kim J Miyamoto S High-mannose glycans are elevated during breast cancer progression. Mol Cell Proteomics (2011) 10:M110002717. 10.1074/mcp.M110.002717 21097542 PMC3013453

[40] Lattova E Varma S Bezabeh T Petrus L Perreault H. Mass spectrometric profiling of N-linked oligosaccharides and uncommon glycoform in mouse serum with head and neck tumor. J Am Soc Mass Spectrom (2008) 19:671–85. 10.1016/j.jasms.2008.01.016 18353675

[41] Everest-Dass AV Briggs MT Kaur G Oehler MK Hoffmann P Packer NH. N-glycan MALDI imaging mass spectrometry on formalin-fixed paraffin-embedded tissue enables the delineation of ovarian cancer tissues. Mol Cell Proteomics (2016) 15:3003–16. 10.1074/mcp.M116.059816 27412689 PMC5013313

[42] Powers TW Holst S Wuhrer M Mehta AS Drake RR. Two-dimensional N-glycan distribution mapping of hepatocellular carcinoma tissues by MALDI-imaging mass spectrometry. Biomolecules (2015) 5:2554–72. 10.3390/biom5042554 26501333 PMC4693247

[43] Hanisch FG Breloy I. Protein-specific glycosylation: signal patches and cis-controlling peptidic elements. Biol Chem (2009) 390:619–26. 10.1515/BC.2009.043 19284292

[44] Liu YC Yen HY Chen CY Chen CH Cheng PF Juan YH Sialylation and fucosylation of epidermal growth factor receptor suppress its dimerization and activation in lung cancer cells. Proc Natl Acad Sci U S A (2011) 108:11332–7. 10.1073/pnas.1107385108 21709263 PMC3136320

[45] Hayes JM Cosgrave EF Struwe WB Wormald M Davey GP Jefferis R Glycosylation and Fc receptors. Curr Top Microbiol Immunol (2014) 382:165–99. 10.1007/978-3-319-07911-0_8 25116100

[46] Stambach NS Taylor ME. Characterization of carbohydrate recognition by langerin, a C-type lectin of Langerhans cells. Glycobiology (2003) 13:401–10. 10.1093/glycob/cwg045 12626394

[47] Deckers J Hammad H Hoste E. Langerhans cells: sensing the environment in health and disease. Front Immunol (2018) 9:93. 10.3389/fimmu.2018.00093 29449841 PMC5799717

[48] Bluth MJ Zaba LC Moussai D Suarez-Farinas M Kaporis H Fan L Myeloid dendritic cells from human cutaneous squamous cell carcinoma are poor stimulators of T-cell proliferation. J Invest Dermatol (2009) 129:2451–62. 10.1038/jid.2009.96 19387481 PMC2846605

[49] van de Ven R Van Den Hout MF Lindenberg JJ Sluijter BJ Van Leeuwen PA Lougheed SM Characterization of four conventional dendritic cell subsets in human skin-draining lymph nodes in relation to T-cell activation. Blood (2011) 118:2502–10. 10.1182/blood-2011-03-344838 21750314

[50] Scott DW Chen J Chacko BK Traylor JG Jr Orr AW Patel RP. Role of endothelial N-glycan mannose residues in monocyte recruitment during atherogenesis. Arterioscler Thromb Vasc Biol (2012) 32:e51–9. 10.1161/ATVBAHA.112.253203 22723438 PMC3831355

[51] Di Piazza M Nowell CS Koch U Durham AD Radtke F. Loss of cutaneous TSLP-dependent immune responses skews the balance of inflammation from tumor protective to tumor promoting. Cancer Cell (2012) 22:479–93. 10.1016/j.ccr.2012.08.016 23079658

[52] Hase S Kikuchi N Ikenaka T Inoue K. Structures of sugar chains of the third component of human complement. J Biochem (1985) 98:863–74. 10.1093/oxfordjournals.jbchem.a135366 4077844

[53] Khan MA Assiri AM Broering DC. Complement and macrophage crosstalk during process of angiogenesis in tumor progression. J Biomed Sci (2015) 22:58. 10.1186/s12929-015-0151-1 26198107 PMC4511526

[54] Pio R Ajona D Lambris JD. Complement inhibition in cancer therapy. Semin Immunol (2013) 25:54–64. 10.1016/j.smim.2013.04.001 23706991 PMC3733085

[55] Riihila P Nissinen L Farshchian M Kivisaari A Ala-Aho R Kallajoki M Complement factor I promotes progression of cutaneous squamous cell carcinoma. J Invest Dermatol (2015) 135:579–88. 10.1038/jid.2014.376 25184960

[56] Ricklin D Hajishengallis G Yang K Lambris JD. Complement: a key system for immune surveillance and homeostasis. Nat Immunol (2010) 11:785–97. 10.1038/ni.1923 20720586 PMC2924908

[57] Shimodaira K Nakayama J Nakamura N Hasebe O Katsuyama T Fukuda M. Carcinoma-associated expression of core 2 beta-1,6-N-acetylglucosaminyltransferase gene in human colorectal cancer: role of O-glycans in tumor progression. Cancer Res (1997) 57:5201–6. 9393734

[58] Machida E Nakayama J Amano J Fukuda M. Clinicopathological significance of core 2 beta1,6-N-acetylglucosaminyltransferase messenger RNA expressed in the pulmonary adenocarcinoma determined by in situ hybridization. Cancer Res (2001) 61:2226–31. 11280791

[59] Tsuboi S Sutoh M Hatakeyama S Hiraoka N Habuchi T Horikawa Y A novel strategy for evasion of NK cell immunity by tumours expressing core2 O-glycans. EMBO J (2011) 30:3173–85. 10.1038/emboj.2011.215 21712812 PMC3160189

[60] Ferreira SA Vasconcelos JL Cavalcanti CL Rego MJ Beltrao EI. Sialic acid differential expression in non-melanoma skin cancer biopsies. Med Mol Morphol (2013) 46:198–202. 10.1007/s00795-013-0025-0 23508708

[61] Cummings RD Etzler ME Antibodies and lectins in glycan analysis. 2nd ed In: Varki A Cummings RD Esko JD Freeze HH Stanley P Bertozzi CR, editors. Essentials of Glycobiology. Harbor, NY: Cold Spring (2009). 20301245

[62] Iskratsch T Braun A Paschinger K Wilson IBH. Specificity analysis of lectins and antibodies using remodeled glycoproteins. Anal Biochem (2009) 386:133–46. 10.1016/j.ab.2008.12.005 19123999

[63] Geisler C Jarvis DL Effective glycoanalysis with *Maackia amurensis* lectins requires a clear understanding of their binding specificities. Glycobiology (2011) 21:988–93. 10.1093/glycob/cwr080 21863598 PMC3130539

[64] Schultz MJ Swindall AF Wright JW Sztul ES Landen CN Bellis SL. ST6Gal-I sialyltransferase confers cisplatin resistance in ovarian tumor cells. J Ovarian Res (2013) 6:25. 10.1186/1757-2215-6-25 23578204 PMC3637436

[65] Cagnoni AJ Perez Saez JM Rabinovich GA Marino KV. Turning-off signaling by siglecs, selectins, and galectins: chemical inhibition of glycan-dependent interactions in cancer. Front Oncol (2016) 6:109. 10.3389/fonc.2016.00109 27242953 PMC4865499

[66] Paulson JC Macauley MS Kawasaki N. Siglecs as sensors of self in innate and adaptive immune responses. Ann N Y Acad Sci (2012) 1253:37–48. 10.1111/j.1749-6632.2011.06362.x 22288608 PMC3335958

[67] Hudak JE Canham SM Bertozzi CR. Glycocalyx engineering reveals a siglec-based mechanism for NK cell immunoevasion. Nat Chem Biol (2014) 10:69–75. 10.1038/nchembio.1388 24292068 PMC3893890

[68] Laubli H Borsig L. Selectins promote tumor metastasis. Semin Cancer Biol (2010) 20:169–77. 10.1016/j.semcancer.2010.04.005 20452433

[69] Pearce OM Laubli H. Sialic acids in cancer biology and immunity. Glycobiology (2016) 26:111–28. 10.1093/glycob/cwv097 26518624

[70] Reddy BV Kalraiya RD. Sialilated beta1,6 branched N-oligosaccharides modulate adhesion, chemotaxis and motility of melanoma cells: effect on invasion and spontaneous metastasis properties. Biochim Biophys Acta (2006) 1760:1393–402. 10.1016/j.bbagen.2006.05.003 16806716

[71] Kolasinska E Przybylo M Janik M Litynska A. Towards understanding the role of sialylation in melanoma progression. Acta Biochim Pol (2016) 63:533–41. 10.18388/abp.2015_1221 27474400

