# Supplementary material for: Alterations of the Human Skin N- and O-Glycome in Basal Cell Carcinoma and Squamous Cell Carcinoma
Source: Front Oncol. 2018 Mar 21;8:70. doi: 10.3389/fonc.2018.00070 (PMC5871710; doi:10.3389/fonc.2018.00070)
Supplement: Supplementary file 6 [file Presentation_1.PDF]

Supplementary material

Alterations of the human skin *N*- and *O*-glycome in basal- and squamous cell carcinoma

Uwe Möginger[1,2§], Sonja Grunewald[3], René Hennig[4,5], Chu-Wei Kuo[6], Falko Schirmeister[1,2], Harald Voth[3], Erdmann Rapp[4,5], Kay-Hooi Khoo[6], Peter H. Seeberger[1, 2], Jan C. Simon[3], Daniel Kolarich[1,7,#]

Affiliations
[1] Department of Biomolecular Systems, Max Planck Institute of Colloids and Interfaces, 14424 Potsdam, Germany
[2] Institute of Chemistry and Biochemistry, Freie Universität Berlin, Germany
[3] Department of Dermatology, Venerology and Allergology, Leipzig University Medical Center, 04103 Leipzig, Germany;
[4] Department of Bioprocess Engineering, Max Planck Institute for Dynamics of complex technical systems, 39106 Magdeburg, Germany
[5] glyXera GmbH, 39122 Magdeburg, Germany
[6] Institute of Biological Chemistry, Academia Sinica, 115 Taipei, Taiwan
[7] Institute for Glycomics, Griffith University, Gold Coast Campus, 4222 Southport, Queensland, Australia

[§] Present address:

University of southern Denmark, Campusvej 55, 5230 Odense

[#] To whom correspondence should be addressed:
Institute for Glycomics, Griffith University, Gold Coast Campus, 4222 Southport, Queensland, Australia
Tel.: +61-7-5552 7026
Email: d.kolarich@griffith.edu.au

Workflow

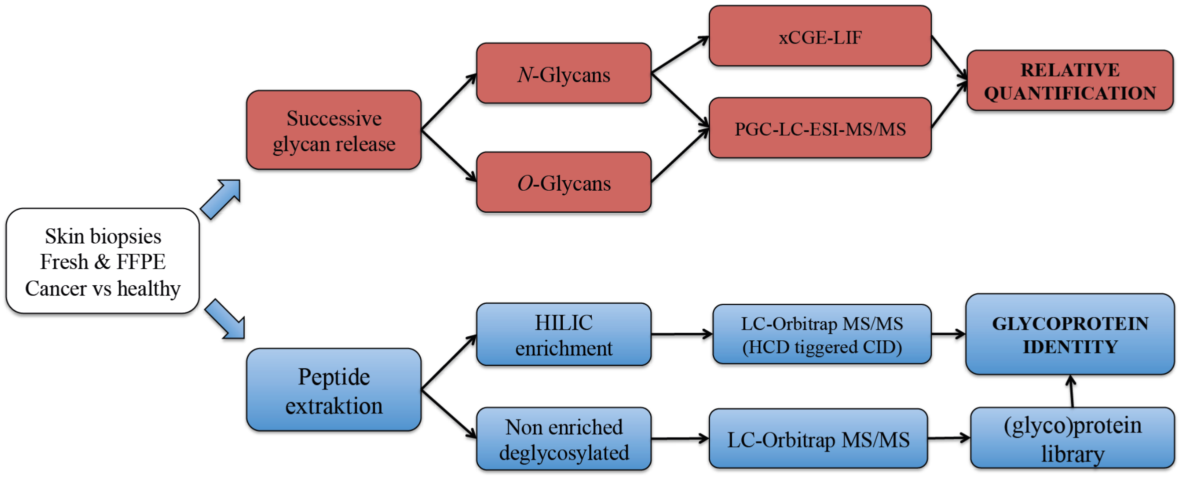

Supplementary Figure 1: Detailed overview on the different glycomics and glycoproteomics methodologies used to assess the skin glycome and glycoproteome.

Supplementary table 1: *N*-Glycans identified in human skin

See separate pdf file

Supplementary table 2: *O*-Glycans identified in human skin

See separate pdf file

Material and Methods

xCGE-LIF sample preparation and measurement

Released unreduced N-glycans were labeled by mixing 2 µL of *N*-glycan solution with 2 µL of 20 mM APTS in 3.6 M citric acid and 2 µL 0.2 M 2-picoline borane complex in dimethyl sulfoxide. The reaction was incubated for 16 h at 37 °C. The labeling reactions was stopped by adding 100 µL of 80% ACN.

Sample clean-up was performed by HILIC-SPE as published earlier (Hennig et al., 2015). Briefly, 200 µL of a 100 mg/mL Bio-Gel P10 (Bio-Rad, Germany) suspension was applied to AcroPrep™ 96-well GHP Filter Plate (Pall Corporation, Germany). Solvents were removed by application of vacuum using a vacuum manifold (Merck-Millipore, Germany). All wells were prewashed with 3 × 200 µL water. Equilibration was performed with 3 × 200 µL 80% ACN. APTS labeled samples were loaded onto BioGel suspension and subsequently washed using 5 × 200 µL 80% ACN containing 100 mM triethylamine, followed by washing 3 × 200 µL 80% ACN. Samples were eluted with 1 × 100 µL and 2 × 200 µL water. The

combined eluates were either analyzed immediately by xCGE-LIF or stored until analysis at − 20°C.

Exoglycosidase digests of APTS labeled *N*-glycans were performed as described earlier (Hennig et al., 2016) using the following enzymes: α(2-3) sialidase (recombinant from *Streptococcus pneumoniae*), α(2-3,6,8) sialidase (recombinant from *Arthrobacter ureafaciens*), α(1-2,3,4,6) fucosidase (from bovine kidney), α(1-2,3,6) mannosidase (from Jack bean) (all from Prozyme, Hayward, CA), α(1-3,4) fucosidase (recombinant from *Xanthomonas*; QABio, San Mateo, CA), β(1-4) galactosidase (recombinant from *Bacteroides fragilis*), β(1-2,3,4,6) *N*-acetylglucosaminidase (recombinant from *S. pneumoniae*) (both New England Biolabs, Ipswich MA). Exoglycosidase digests were performed under reaction conditions recommended by the suppliers. All enzymes were carefully tested for reactivity and specificity by incubation with APTS labeled *N*-glycans derived from bovine fetuin (for α(2-3) and α(2-3,6,8) sialidase), bovine IgG (for α(1-2,3,4,6) fucosidase, β(1-4) galactosidase and β(1-2,3,4,6) *N*-acetylglucosaminidase), bovine ribonuclease B (for α(1-2,3,6) mannosidase) and human lactotransferrin (for α(1-3,4) fucosidase).

xCGE-LIF analyses were performed by mixing 3 µL of HILIC-SPE eluates or APTS labeled *N*-glycans after exoglycosidase digest with 1 µL GeneScan™ 500 LIZ™ dye size standard (1:50 diluted in Hi-Di™ Formamide, both Thermo Fisher Scientific), 1 µL 2nd NormMix (glyXera GmbH) and 7 µL Hi-Di™ Formamide. xCGE-LIF measurements were performed using a 3130xl Genetic Analyzer, equipped with a 50 cm capillary array, filled with POP-7™ polymer. The samples were electrokinetically injected and separated for 40 min with a running voltage of 15 kV. The recorded electropherograms were normalized to the internal standards by glyXtool (glyXera GmbH), generating so called "*N*-glycan fingerprints" (example shown in Supplementary Figure 9).

Supplementary Table 3: MS settings

Ion trap settings (Bruker Amazon ETD Speed)	
**Capillary voltage**	1-1.3 kV
**SPS**	*m/z* 900
**Compound stability**	100%
**Trap Drive Level**	100%
**Spectra averaging**	5
**Dry gas temperature**	150°C
**Dry Gas flow**	3 L/min
**Maximum accumulation time**	200 ms
**Ion mode**	negative
**<u>MS-Scan</u>**	
**MS Scan mode**	ultrascan
**ICC target**	40000

**Mass detection range**	*m/z* 350-1800
**<u>MSMS Scan</u>**	
**MS scan mode**	ultrascan
**SPS MS(n)**	automatic
**MS(n) spectra averages**	5
**MS(n) ICC target**	150000
**Preferred charge state**	None
**Active exclusion**	Off
**Mass detection range**	*m/z* 100-2500
**Isolation width**	3 Da
**Exclude singly charged ions**	off
**No. of precursor ions**	3
**SmartFrag**	Enhanced
**SmartFrag Start amplitude**	30%
**SmartFrag End amplitude**	120%
**Fragmentation width**	5 *m/z*

**Orbitrap settings (Thermo Orbitrap Trybrid Fusion)**	
**<u>MS Scan</u>**	
**Resolution**	120000
**Scan range**	400-2000
**Microscans**	1
**Max injection time**	60 ms
**polarity**	positve
**datatype**	profile
**AGC target**	2.0e5
**trigger ions**	204.0867, 366.1396
**Dynamic exclusion after n times**	1
**Included charge states**	2-6
**Filter precursor selection range**	600-1800
**<u>MSMS Scan</u>**	

Scan event 1	
**Charge range**	2-4
**Activation type**	HCD
**Isolation mode**	Quadrupole
**Isolation window**	1.6
**Stepped collision Energy (%)**	5
**Collision energy**	28
**Detection type**	Orbitrap
**Resolution**	30000
**Scan Range**	350-1000
**Max injection time (ms)**	75
**AGC target**	100000
**Activation type**	CID
**Isolation mode**	Quadrupole
**Isolation window**	1.6
**Collision energy**	30%
**Detector type**	Iontrap
**Ion trap scan rate**	Rapid
**trigger ions**	204.0867, 366.1396
**Max ion injection time**	60
**AGC target**	10000
**Data type**	Centroid
**Scan event 2**	
**Charge state**	5-6
**Activation type**	HCD
**Isolation mode**	Quadrupole
**Isolation window**	1.6
**Collision energy (%)**	30
**Detector type**	Orbitrap
**Resolution**	30000
**Scan Range**	350-10000
**Max injection time (ms)**	60

**AGC target**	50000
**Activation type**	ETD
**Scan range mode**	Auto normal
**Trigger ions**	204.0867, 366.1396
**First Mass**	120
**multi notch isolation**	False
**Use calibrated charge dependent ETD parameters**	True
**Isolation mode**	Quadrupole
**Isolation window**	1.6
**Collision energy**	30%
**Detector type**	Orbitrap
**Resolution**	30000
**Max ion injection time**	100
**AGC target**	100000
**Data type**	Profile

Supplementary Table 4: Patient Data of snap frozen skin biopsy samples

Patient	Sex	Age (years)	Localization	Clinical: tumor length (mm)	Clinical: tumor width (mm)	Tumor type (histology)	Ulceration	Tumor invasion level	Tumor thickness (mm)
1	m	74	Lower Leg	14	11	nod BCC	yes	IV	2.9
2	m	79	Shoulder	20	10	nod BCC	no	IV	1.5
3	m	79	Shoulder	5	5	nod BCC	no	IV	1.6
4	m	74	Lower Leg	20	18	nod BCC	yes	V	5.8
5	m	73	Back	25	25	nod BCC	no	IV	4.9
6	m	72	Chest	25	25	nod BCC	yes	IV	0.8
7	m	82	Shoulder	5	5	nod BCC	yes	IV	1.2
8	m	90	Back	9	5	nod BCC	no	III	0.4
9	m	88	Shoulder	7	8	nod BCC	yes	IV	3.5
10	f	80	Cheek	6	5	nod BCC	no	IV	2.5
11	m	70	Chest	12	10	nod BCC	yes	IV	1.9
12	m	82	Shoulder	10	10	nod BCC	yes	IV	1.8
13	m	75	Shoulder	17	14	nod BCC	yes	IV	3.9
14	m	80	Shoulder	10	10	nod BCC	yes	IV	2.0

Clinical and dermato-pathological data: tumor and control sample were taken from the same patient. Clinical tumor length and width was determined prior to surgery; tumor type, ulceration, invasion level and tumor thickness was assessed by dermatopathology *nod:* nodulocystic BCC; *inf-scl* infiltrative sclerosing BCC.

Supplementary Table 5: BCC Patient Data of formalin fixed paraffin embedded skin samples

Patient	Sex	Age (years)	Localization	Clinical: tumor length (mm)	Clinical: tumor width (mm)	Tumor type (histology)	Ulceration	Tumor invasion level	Tumor thickness (mm)
1	m	71	Nose	10	7	nod BCC	yes	IV	2.5
2	f	25	Ear	15	9	nod BCC	no	III	0,3
3	m	88	Nose	11	10	nod BCC	no	IV	2.0
4	f	25	Head	15	8	nod BCC	no	III	0.3
5	m	79	Back	15	11	sup BCC	no	III	0.3
6	m	79	Neck	18	9	nod BCC	yes	IV	1.9
7	f	72	Cheek	21	10	inf-scl BCC	yes	IV	2.9
8	f	75	Nose	13	10	nod BCC	no	IV	1.8
9	m	90	Chest	30	13	nod BCC	yes	IV	3.8
10	m	62	Nose	10	10	nod BCC	yes	IV	3.9
11	f	73	Nose	15	15	nod BCC	yes	IV	3.0
12	f	82	Nose	10	10	nod BCC	no	IV	4.0
13	m	72	Cheek	10	10	inf-scl BCC	yes	IV	1.5
14	m	62	Shoulder	60	30	nod BCC	yes	IV	3.0
15	f	77	Forehead	15	10	nod BCC	no	IV	3.2
16	f	80	Chin	10	10	nod BCC	yes	IV	2.0
17	m	84	Lower Leg	20	15	nod BCC	yes	IV	2.6
18	m	81	Cheek	15	10	nod BCC	yes	IV	7.1
19	m	77	Cheek	9	3	inf-scl BCC	no	IV	2.0
20	m	58	Nose	8	8	nod BCC	yes	V	4.0

Clinical and dermato-pathological data: : tumor and control sample were taken from the same patient. Clinical tumor length and width was determined prior to surgery; tumor type, ulceration, invasion level a tumor thickness was assessed by dermatopathology *nod:* nodulocystic BCC; *sup* superficial BCC; *inf-scl* infiltrative sclerosing BCC.

Supplementary Table 6: SCC Patient Data of formalin fixed paraffin embedded skin samples:

Patient	Sex	Age (years)	Localization	Clinical: tumor length (mm)	Clinical: tumor width (mm)	Tumor type (histology)	Ulceration	Tumor invasion level	Tumor thickness (mm)
1	f	65	Cheek	8	5	SCC, G1	yes	IV	2.0
2	m	85	Head	50	50	SCC, G2	yes	V	14
3	f	72	Head	10	10	SCC, G2	yes	IV	10.8
4	m	90	Head	18	10	SCC, G2	yes	IV	2.8
5	m	72	Nose	15	15	SCC, G2	no	V	5
6	f	74	Cheek	10	5	SCC, G1	no	IV	3.5
7	f	73	Upper trunk	5	5	SCC, G1	yes	II	0.6
8	m	76	Cheek	28	22	SCC, G2	no	IV	4.2
9	m	74	Head	17	14	SCC, G1	no	IV	1.9
10	f	79	Neck	20	10	SCC, G2	no	IV	1.9
11	m	90	Head	30	30	SCC, G2	no	V	4.1
12	m	88	Head	10	8	SCC, G1	yes	IV	1.0
13	m	64	Head	22	21	SCC, G1	yes	IV	2.6
14	m	87	Ear	45	15	SCC, G2	yes	V	7
15	m	78	Head	23	20	SCC, G2	yes	V	5

Clinical and dermato-pathological data: : tumor and control sample were taken from the same patient. Clinical tumor length and width was determined prior to surgery; tumor type, ulceration, invasion level a tumor thickness was assessed by dermatopathology *nod:* nodulocystic BCC; *sup* superficial BCC; *inf-scl* infiltrative sclerosing BCC.

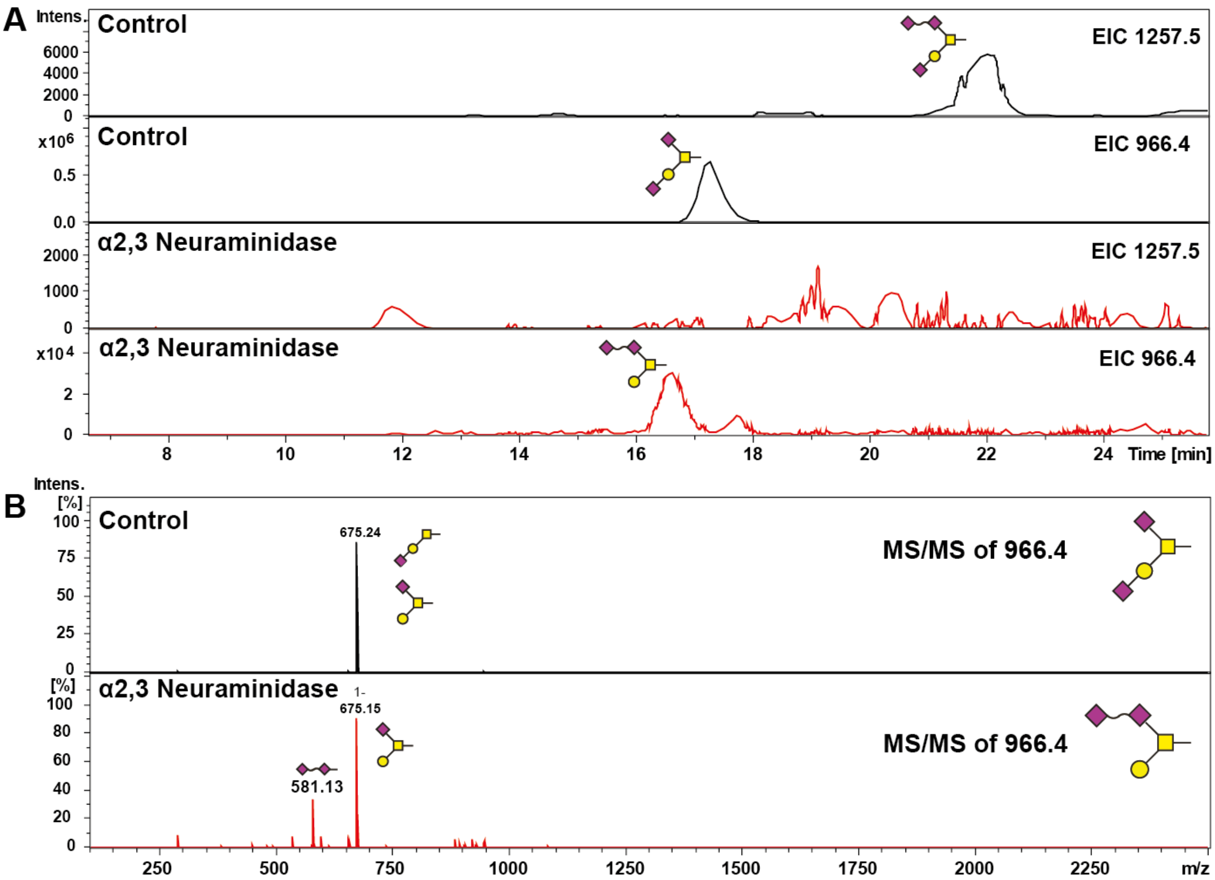

Supplementary Figure 2: α2-3 neuraminidase digest of *O*-glycans. The triply sialylated *O*-glycan *m/z*= 1257.5 is not detectable after the α2-3 neuraminidase digest. Instead a peak with *m/z*= 966.4 appears. B) Product ion spectra of *m/z* = 966.4. The fragment ion *m/z* = 581.1 of two linked sialic acids prove that the 966.4 peak in the α2-3-neuraminidase digest is derived form the triply sialylated *O*-glycan *m/z* = 1257.5. Furthermore, the spectra indicate that the two sialic acids are linked to the 6-arm of the reducing end GalNAc.

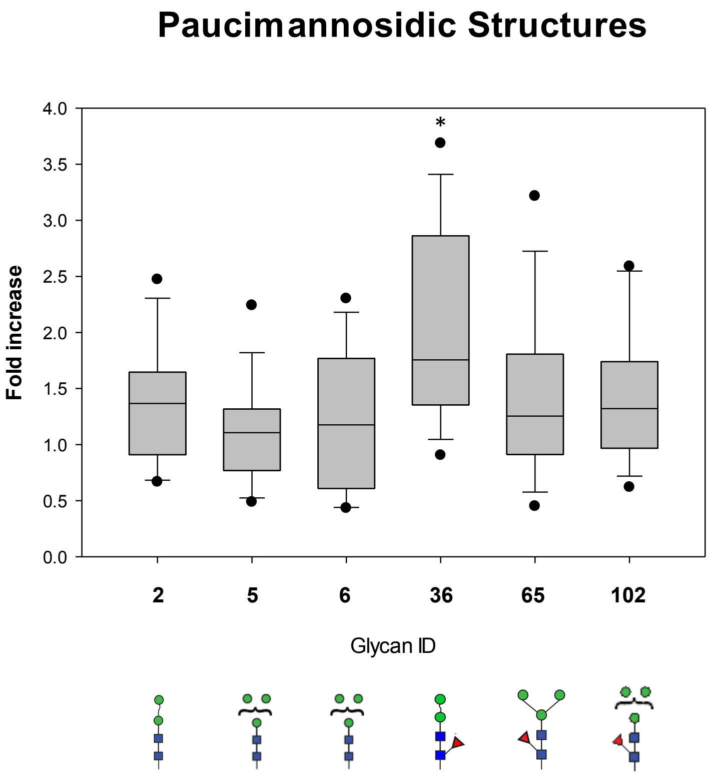

Glycan ID	2	5	6	36	65	102
P-value	0.085	0.982	0.909	0.006*	0.346	0.118

Supplementary Figure 3: Evaluation of paucimannosidic structures. Box plot of ratios tumor/control for 14 patients. Samples were assessed for normal distribution by a Shapiro-Wilk test. As most of the glycans did not show normal distribution a Mann-Whitney Rank Sum Test was used for statistical evaluation. Testing resulted in significance for structure 36 only. Upregulation in other structures were found not significant.

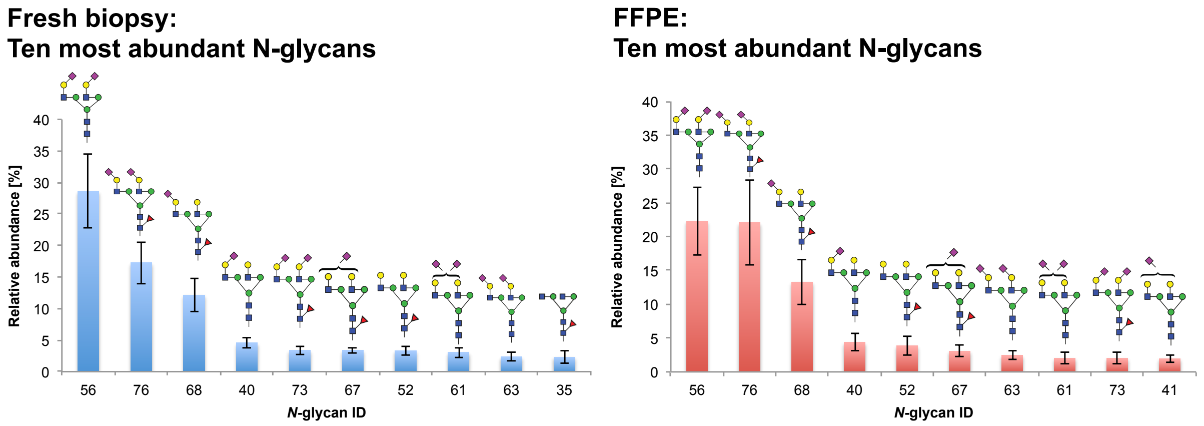

Supplementary Figure 4: Comparison between the ten most abundant *N*-glycans (representing 80% of total *N*-glycan abundance) in biopsy and FFPE. The abundance of α2-6 sialylated is lower in FFPE whereas α2-3 sialylated structures are more abundant.

Supplementary Table 7: Statistical analysis of *O*-glycans from fresh biopsies

ID	Sample	N	Mean	Std Dev	SEM	p-value		Test
1	Control	9	3.043	1.052	0.351	0.00274	*	t-test
	Tumor	9	1.186	1.173	0.391			
2	Control	9	0.861	0.22	0.0734	0.112		Mann-Whitney
	Tumor	9	0.754	0.604	0.201			
3	Control	9	13.19	2.49	0.83	0.025	*	t-test
	Tumor	9	10.498	2.132	0.711			
4	Control	9	2.257	1.168	0.389	0.614		t-test
	Tumor	9	2.012	0.824	0.275			
5	Control	9	44.436	4.708	1.569	0.0013	*	t-test
	Tumor	9	35.101	5.443	1.814			
6	Control	9	1.092	0.420	0.140	0.013	*	t-test
	Tumor	9	0.606	0.311	0.104			
7	Control	9	1.327	0.477	0.159	0.251		Mann-Whitney
	Tumor	9	2.308	1.823	0.608			
8	Control	9	0.754	0.377	0.126	0.013	*	Mann-Whitney
	Tumor	9	2.228	1.601	0.534			
9	Control	9	7.615	2.988	0.996	0.93		Mann-Whitney
	Tumor	9	7.262	2.54	0.847			
10	Control	9	2.196	0.927	0.309	0.369		t-test
	Tumor	9	1.773	1.012	0.337			
11	Control	9	6.642	1.662	0.554	0.0081	*	t-test
	Tumor	9	11.003	3.997	1.332			
12	Control	9	14.008	4.786	1.595	0.0427		t-test
	Tumor	9	18.98	4.798	1.599			
13	Control	9	2.561	0.734	0.245	0.003	*	Mann-Whitney
	Tumor	9	6.281	3.026	1.009			

Glycan ID according to supplementary table 1. Relative abundance is given percent (%). Significance ($p \leq 0.04$) is indicated by asterisk (*).

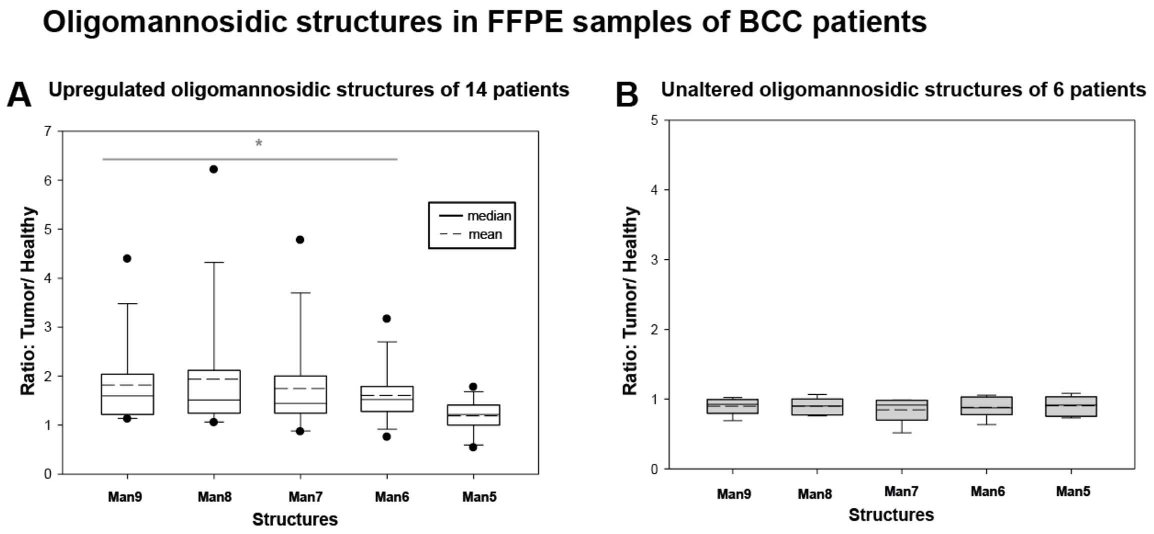

C

Glycan		Glycan ID	Sample	N	Mean (%)	Std Dev	SEM	p-value
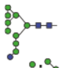	Man9+Glc	22	Control<br>Tumor	detected but not quantifiable				
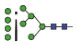	Man9	12	Control	14	1.68	0.96	0.26	0.0191*
			Tumor	14	2.72	1.23	0.33	
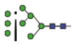	Man8	10/11	Control	14	2.38	1.27	0.34	0.0065*
			Tumor	14	4.04	1.67	0.45	
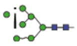	Man7	8	Control	14	2.04	0.94	0.25	0.0113*
			Tumor	14	3.19	1.25	0.34	
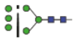	Man6	14	Control	14	2.08	1.02	0.27	0.0241*
			Tumor	14	3.07	1.17	0.31	
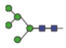	Man5	42	Control	14	2.01	0.91	0.24	0.3900
			Tumor	14	2.32	0.94	0.25	
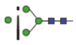	Man4	21	Control<br>Tumor	detected but not quantifiable				

D

Core type	Sample	N	Mean (%)	Std Dev	SEM	p-value	Test
Core-1	Control	20	55.264	13.197	2.951	0.000025 *	t-test
	Tumor	20	37.436	10.132	2.266		
Core-2	Control	20	44.736	13.197	2.951	0.000025 *	t-test
	Tumor	20	62.564	10.132	2.266		

Supplementary Figure 5: Comparison of oligomannose *N*-glycans obtained from formalin fixed paraffin embedded (FFPE) slides. Significance ($p \leq 0.04$) is indicated by asterisk (*). **A)** Box plot of ratio: Tumor/Healthy shows Upregulation of 1.5 fold found for all structures except Man5 in 14 out of 20 patients. Signal to noise ratios for Man9+Glc and Man4 were too low for reliable quantitation **B)** Box plot of ratio: Tumor/Healthy for six patients not showing any upregulation of high-mannose structures. **C)** Statistical evaluation by of the 14 patients using a t-test. **D)** Comparison of core-1 and core-2 abundance between FFPE tissue of healthy and BCC samples

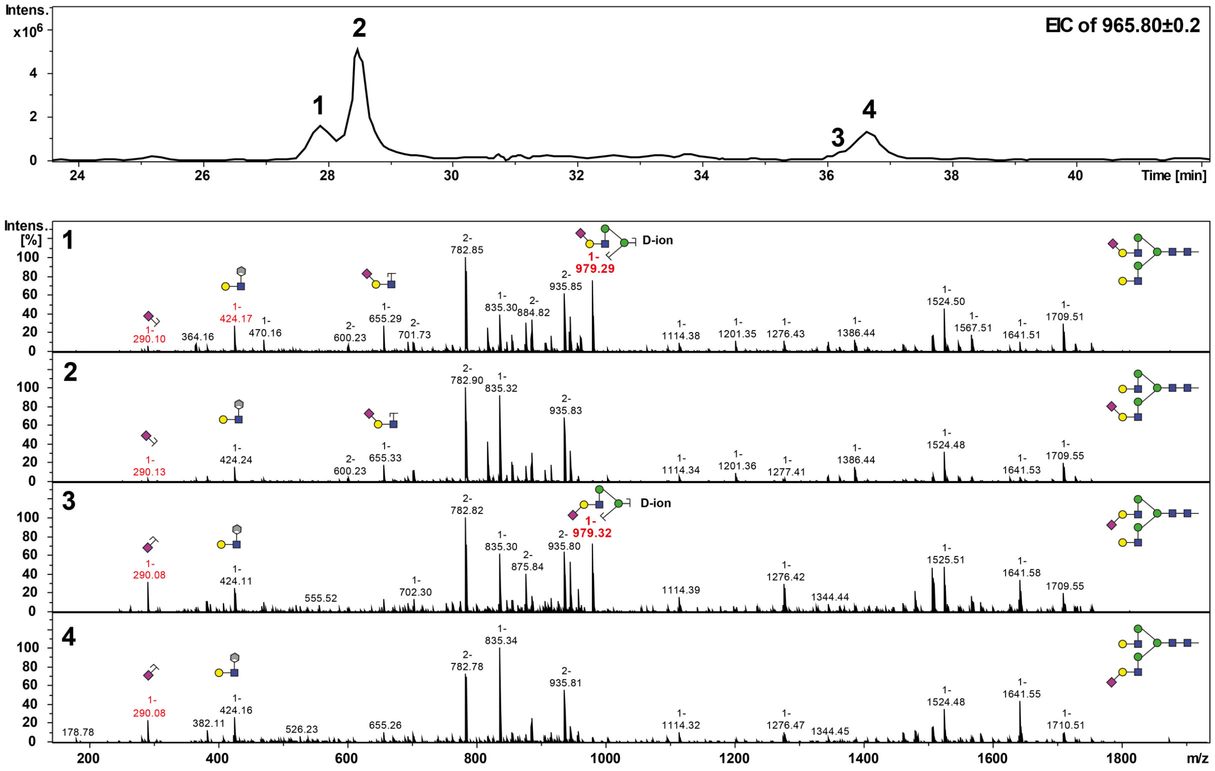

Supplementary Figure 6: EIC and product ion spectra of *m/z*= 965.80. The graph illustrates the differentiation of arm isomers as well as α2-3- and α2-6 linkage isomers of sialic acid. Sialic acid on the six arm results in a very prominent D-ion *m/z*= 979. NeuAc attached by an α2-3 linkage results in considerable higher retention time (EIC peak no. 3 and 4) as well as in a more prominent NeuAc B–ion *m/z*= 290. The strong presence of *m/z* = 424 indicates the presence of a terminal LacNAc moiety.

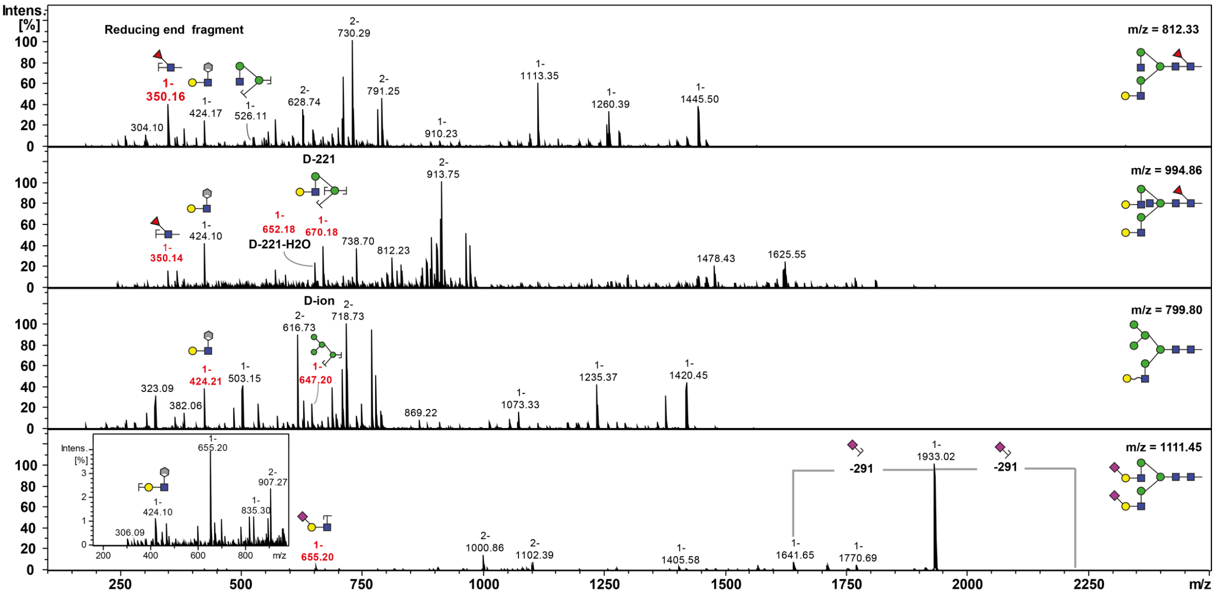

Supplementary Figure 7: product ion spectra of *m/z*= 812.33, 994.86, 799.80, 1111.45. The graph illustrates the differentiation of assignment of fucose to the core, bisecting GlcNAc and presence of two NeuAc. Core fucose is indicated by the presence of *m/z* =350 which derives from the reducing end GlcNAc and the attached fucose. The chemical reduction of the reducing end acts as a mass tag of 2 Da. Presence of a bisecting GlcNAc is indicated by the presence of the D- 221 ion (*m/z* = 670) as well a signal derived from loss of water of the very same fragment (*m/z* = 652). Hybrid structures can be identified by the presence of the D-ion and an additional signal that indicates a LacNAc structure (*m/z* = 424). The biantennary doubly sialylated structures readily lose one NeuAc moieties indicated by neutral losses of 291 Da. As stated before the *m/z* 424 peak is only prominent if one or two terminal LacNAc moieties are present and is therefore very low abundant in the case of parent ion *m/z* 1111.4.

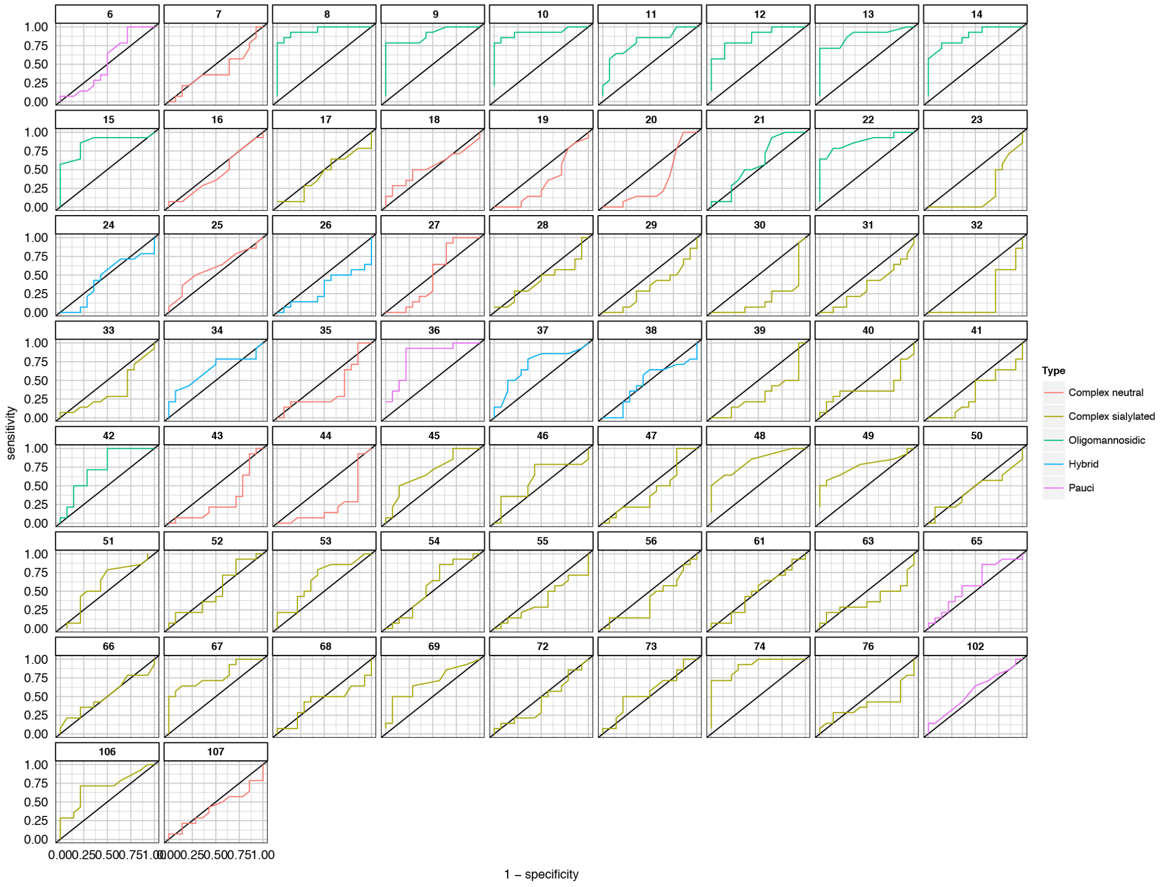

Supplementary Figure 8: Receiver operator characteristics (ROC) of 65 quantified glycan species. Numbering is according to *N*-gycans in the glycan list.

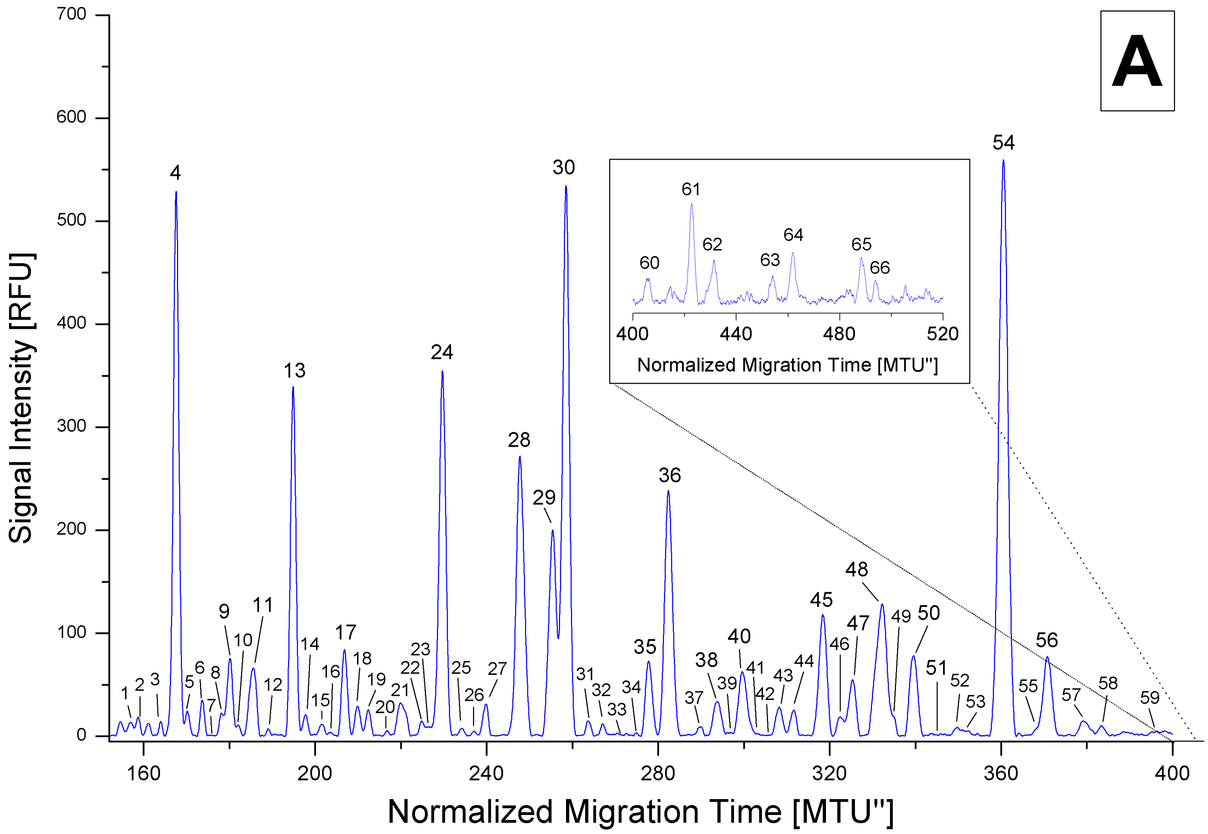

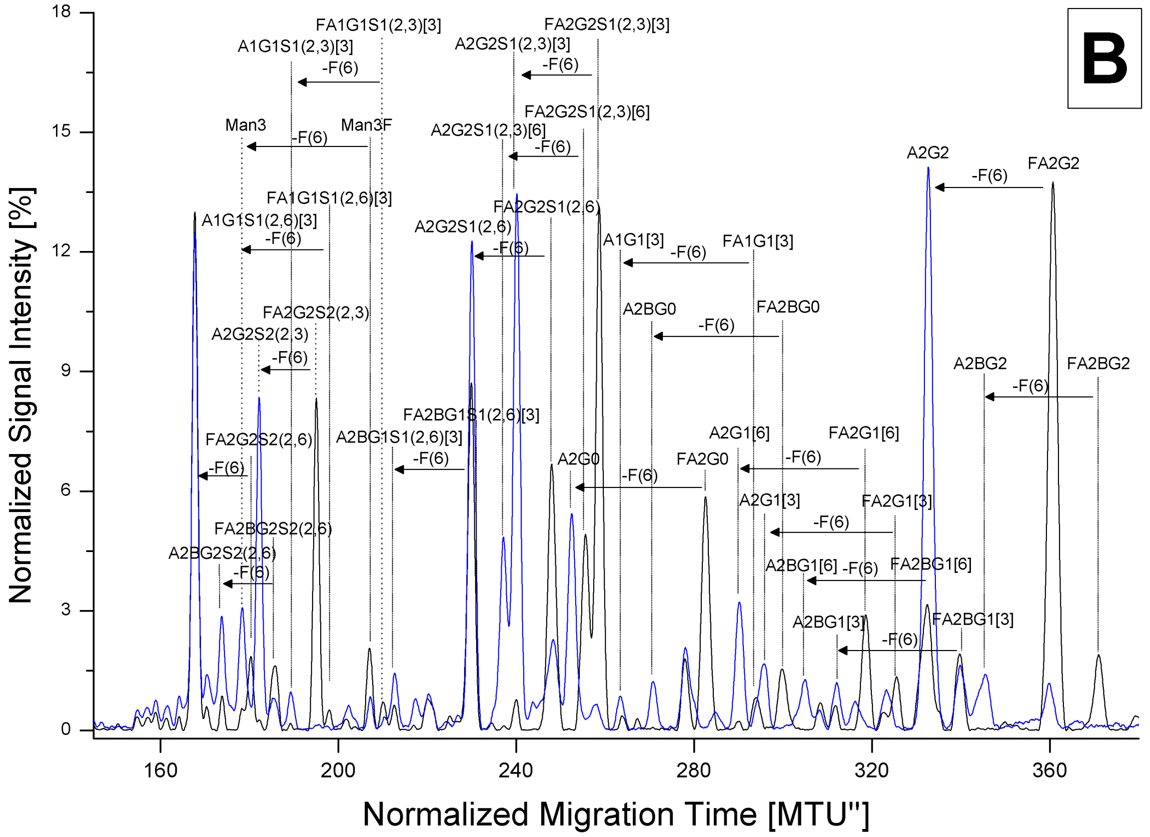

Supplementary Figure 9: xCGE-LIF generated *N*-glycan fingerprints.

A) Fingerprint of a representative skin *N*-glycome, labeled with APTS and analyzed by xCGE-LIF. Signal intensity in relative fluorescence units [RFU] is plotted over the

normalized migration time units [MTU'']. Numbers indicate the 66 most abundant *N*-glycan peaks, picked in the *N*-glycan fingerprints. The annotation and the relative quantification of the picked peaks can be found in Supplementary Table "xCGE-LIF quant.xlsx". The inset shows the later migration time range of the xCGE-LIF fingerprint measurement. **B)** Fingerprint of a representative skin *N*-glycome before (black) and after (blue) α(1-2,3,4,6) fucosidase treatment. Normalized signal intensity (% of the total peak height) is plotted over the normalized migration time units [MTU'']. Arrows indicate the peak shift due to a lost core fucose (α1-6 linked) after fucosidase treatment. For a graphical *N*-glycan representation of annotated names see Supplementary Table "xCGE-LIF quant.xlsx".

Supplementary table 8: Summary of all identified glycopeptides from FFPE and Biopsy samples in tumor and healthy tissue.

Peptide sequence	Glycans NHFAGNa	Modification Type(s)	Observed m/z	z	Observed (M+H)	Calc. mass (M+H)	Mass error (ppm)	Cleavage	Score	Delta	Delta Mod	|Log Prob|	Protein Name
R.AYWPDVIHSFPN[+2350.830]R.S	HexNAc(4)Hex(5)Fuc(1)NeuAc(2)	N[+2351]	1318.222	3	3952.652	3952.616	8.9	Specific	184.4	53.7	53.7	0.50	>sp|O00584|RNT2_HUMAN Ribonuclease T2 OS=Homo sapiens GN=RNASET2 PE=1 SV=2
R.AYWPDVIHSFPN[+2059.735]R.S	HexNAc(4)Hex(5)Fuc(1)NeuAc(1)	N[+2060]	916.140	4	3661.539	3661.521	5.0	Specific	158.3	54.3	54.3	0.48	>sp|O00584|RNT2_HUMAN Ribonuclease T2 OS=Homo sapiens GN=RNASET2 PE=1 SV=2
R.AYWPDVIHSFPN[+1768.640]R.S	HexNAc(4)Hex(5)Fuc(1)	N[+1769]	843.366	4	3370.441	3370.425	4.6	Specific	145.1	53.3	53.3	0.08	>sp|O00584|RNT2_HUMAN Ribonuclease T2 OS=Homo sapiens GN=RNASET2 PE=1 SV=2
K.DLDTDFTNN[+2204.772]ASQPETK.A	HexNAc(4)Hex(5)Fuc(0)NeuAc(2)	N[+2205]	1000.903	4	4000.592	4000.571	5.4	Specific	127.6	10.0	10.0	0.27	>sp|O75976|CBPD_HUMAN Carboxypeptidase D OS=Homo sapiens GN=CPD PE=1 SV=2
K.HLASLYANNHPSM[+15.995]HMGQPSC[+57.021]PN[+2350.830]K.S	HexNAc(4)Hex(5)Fuc(1)NeuAc(2)	C[+57], M[+16], N[+2351]	1240.510	4	4959.020	4957.995	4.2	Specific	99.8	99.8	26.3	0.16	>sp|O75976|CBPD_HUMAN Carboxypeptidase D OS=Homo sapiens GN=CPD PE=1 SV=2
K.DASINIENMQFIHN[+1768.640]GTYIC[+57.021]DVK.N	HexNAc(4)Hex(5)Fuc(1)	C[+57], N[+1769]	1088.721	4	4351.863	4350.841	4.2	Specific	248.1	16.0	16.0	2.98	>sp|O95297-2|MPZL1_HUMAN Isoform 2 of Myelin protein zero-like protein 1 OS=Homo sapiens GN=MPZL1
R.YHYN[+1540.529]GTFLDGTLFDSSHNR.M	HexNAc(2)Hex(7)	N[+1541]	946.891	4	3784.541	3784.539	0.5	Specific	452.9	214.9	214.9	3.16	>sp|O95302-3|FKBP9_HUMAN Isoform 3 of Peptidyl-prolyl cis-trans isomerase FKBP9 OS=Homo sapiens GN=FKBP9
R.YHYN[+1702.581]GTFLDGTLFDSSHNR.M	HexNAc(2)Hex(8)	N[+1703]	987.407	4	3946.608	3946.592	4.1	Specific	622.8	383.6	383.6	9.59	>sp|O95302-3|FKBP9_HUMAN Isoform 3 of Peptidyl-prolyl cis-trans isomerase FKBP9 OS=Homo sapiens GN=FKBP9
R.YHYN[+1864.634]GTLLDGTLFDSSYSR.N	HexNAc(2)Hex(9)	N[+1865]	1358.561	3	4073.670	4073.654	3.9	Specific	445.6	342.0	342.0	6.08	>sp|O95302-3|FKBP9_HUMAN Isoform 3 of Peptidyl-prolyl cis-trans isomerase FKBP9 OS=Homo sapiens GN=FKBP9
K.EHEGAIYPDN[+1622.582]TTDFQR.A	HexNAc(4)Hex(5)	N[+1623]	1172.482	3	3515.432	3515.423	2.8	Specific	159.6	145.1	145.1	2.84	>sp|P00450|CERU_HUMAN Ceruloplasmin OS=Homo sapiens GN=CP PE=1 SV=1
R.YTITAYLSAN[+2204.772]ITFYTGVPK.A	HexNAc(4)Hex(5)Fuc(0)NeuAc(2)	N[+2205]	1082.976	4	4328.883	4327.878	0.3	Specific	52.2	0.2	0.2	2.12	>sp|P00488|F13A_HUMAN Coagulation factor XIII A chain OS=Homo sapiens GN=F13A1 PE=1

<table>
<tr><th></th><th></th><th></th><th></th><th></th><th></th><th></th><th></th><th></th><th></th><th></th><th></th><th></th><th></th></tr>
<tr><td></td><td></td><td></td><td></td><td></td><td></td><td></td><td></td><td></td><td></td><td></td><td></td><td></td><td>SV=4</td></tr>
<tr><td>R.YTITAYLSAN[+1622.582]ITFYTGVPK.A</td><td>HexNAc(4)Hex(5)</td><td>N[+1623]</td><td>1249.233</td><td>3</td><td>3745.684</td><td>3745.688</td><td>-0.9</td><td>Specific</td><td>115.9</td><td>32.5</td><td>32.5</td><td>2.33</td><td>>sp|P00488|F13A_HUMAN Coagulation factor XIII A chain OS=Homo sapiens GN=F13A1 PE=1 SV=4</td></tr>
<tr><td>R.YTITAYLSAN[+1913.677]ITFYTGVPK.A</td><td>HexNAc(4)Hex(5)Fuc(0)NeuAc(1)</td><td>N[+1914]</td><td>1010.200</td><td>4</td><td>4037.780</td><td>4036.783</td><td>-1.6</td><td>Specific</td><td>133.6</td><td>27.4</td><td>27.4</td><td>0.77</td><td>>sp|P00488|F13A_HUMAN Coagulation factor XIII A chain OS=Homo sapiens GN=F13A1 PE=1 SV=4</td></tr>
<tr><td>K.MVSHHN[+1622.582]LTTGATLINEQWLLTTAK.N</td><td>HexNAc(4)Hex(5)</td><td>N[+1623]</td><td>1076.502</td><td>4</td><td>4302.984</td><td>4301.974</td><td>1.6</td><td>Specific</td><td>302.5</td><td>279.1</td><td>163.6</td><td>6.75</td><td>>sp|P00738|HPT_HUMAN Haptoglobin OS=Homo sapiens GN=HP PE=1 SV=1</td></tr>
<tr><td>K.VVLHPN[+1622.582]YSQVDIGLIK.L</td><td>HexNAc(4)Hex(5)</td><td>N[+1623]</td><td>1139.872</td><td>3</td><td>3417.600</td><td>3417.593</td><td>2.1</td><td>Specific</td><td>371.4</td><td>321.6</td><td>321.6</td><td>5.91</td><td>>sp|P00738|HPT_HUMAN Haptoglobin OS=Homo sapiens GN=HP PE=1 SV=1</td></tr>
<tr><td>K.VVLHPN[+1622.582]YSQVDIGLIK.L</td><td>HexNAc(4)Hex(5)</td><td>N[+1623]</td><td>1139.872</td><td>3</td><td>3417.602</td><td>3417.593</td><td>2.7</td><td>Specific</td><td>548.7</td><td>499.0</td><td>499.0</td><td>8.77</td><td>>sp|P00738|HPT_HUMAN Haptoglobin OS=Homo sapiens GN=HP PE=1 SV=1</td></tr>
<tr><td>K.VVLHPN[+1913.677]YSQVDIGLIK.L</td><td>HexNAc(4)Hex(5)Fuc(0)NeuAc(1)</td><td>N[+1914]</td><td>927.928</td><td>4</td><td>3708.692</td><td>3708.688</td><td>1.0</td><td>Specific</td><td>227.7</td><td>227.7</td><td>227.7</td><td>4.42</td><td>>sp|P00738|HPT_HUMAN Haptoglobin OS=Homo sapiens GN=HP PE=1 SV=1</td></tr>
<tr><td>K.VVLHPN[+2204.772]YSQVDIGLIK.L</td><td>HexNAc(4)Hex(5)Fuc(0)NeuAc(2)</td><td>N[+2205]</td><td>1333.933</td><td>3</td><td>3999.784</td><td>3999.784</td><td>0.2</td><td>Specific</td><td>230.8</td><td>230.8</td><td>230.8</td><td>3.67</td><td>>sp|P00738|HPT_HUMAN Haptoglobin OS=Homo sapiens GN=HP PE=1 SV=1</td></tr>
<tr><td>K.WVSN[+1622.582]K.T</td><td>HexNAc(4)Hex(5)</td><td>N[+1623]</td><td>1128.467</td><td>2</td><td>2255.927</td><td>2255.917</td><td>4.2</td><td>Specific</td><td>122.4</td><td>115.5</td><td>115.5</td><td>0.15</td><td>>sp|P01008|ANT3_HUMAN Antithrombin-III OS=Homo sapiens GN=SERPINC1 PE=1 SV=1</td></tr>
<tr><td>K.YLGN[+1913.677]ATAIFFLPDEGK.L</td><td>HexNAc(4)Hex(5)Fuc(0)NeuAc(1)</td><td>N[+1914]</td><td>1223.864</td><td>3</td><td>3669.578</td><td>3669.572</td><td>1.6</td><td>Specific</td><td>207.8</td><td>174.8</td><td>174.8</td><td>2.92</td><td>>sp|P01009-2|A1AT_HUMAN Isoform 2 of Alpha-1-antitrypsin OS=Homo sapiens GN=SERPINA1</td></tr>
<tr><td>K.YLGN[+1622.582]ATAIFFLPDEGK.L</td><td>HexNAc(4)Hex(5)</td><td>N[+1623]</td><td>1126.833</td><td>3</td><td>3378.483</td><td>3378.477</td><td>1.8</td><td>Specific</td><td>270.1</td><td>235.9</td><td>235.9</td><td>4.35</td><td>>sp|P01009-2|A1AT_HUMAN Isoform 2 of Alpha-1-antitrypsin OS=Homo sapiens GN=SERPINA1</td></tr>
<tr><td>K.YLGN[+2204.772]ATAIFFLPDEGK.L</td><td>HexNAc(4)Hex(5)Fuc(0)NeuAc(2)</td><td>N[+2205]</td><td>990.924</td><td>4</td><td>3960.674</td><td>3960.668</td><td>1.6</td><td>Specific</td><td>485.1</td><td>417.0</td><td>417.0</td><td>9.03</td><td>>sp|P01009|A1AT_HUMAN Alpha-1-antitrypsin OS=Homo sapiens GN=SERPINA1 PE=1 SV=3</td></tr>
<tr><td>R.QLAHQSN[+1622.582]STNIFFSPVSIATAFAMLSLGTK.A</td><td>HexNAc(4)Hex(5)</td><td>N[+1623]</td><td>1202.063</td><td>4</td><td>4805.229</td><td>4804.217</td><td>1.9</td><td>Specific</td><td>275.4</td><td>275.4</td><td>66.7</td><td>6.35</td><td>>sp|P01009|A1AT_HUMAN Alpha-1-antitrypsin OS=Homo sapiens GN=SERPINA1 PE=1 SV=3</td></tr>
<tr><td>R.QLAHQSN[+1622.582]STNIFFSPVSIATAFAM[+15.995]LSLGTK.A</td><td>HexNAc(4)Hex(5)</td><td>M[+16], N[+1623]</td><td>1206.063</td><td>4</td><td>4821.228</td><td>4820.212</td><td>2.8</td><td>Specific</td><td>179.1</td><td>179.1</td><td>35.7</td><td>3.89</td><td>>sp|P01009|A1AT_HUMAN Alpha-1-antitrypsin OS=Homo sapiens GN=SERPINA1 PE=1 SV=3</td></tr>
<tr><td>K.YLGN[+1913.677]ATAIFFLPDEGK.L</td><td>HexNAc(4)Hex(5)Fuc(0)NeuAc(1)</td><td>N[+1914]</td><td>1223.866</td><td>3</td><td>3669.583</td><td>3669.572</td><td>2.9</td><td>Specific</td><td>509.1</td><td>427.9</td><td>427.9</td><td>8.79</td><td>>sp|P01009|A1AT_HUMAN Alpha-1-antitrypsin OS=Homo sapiens GN=SERPINA1 PE=1 SV=3</td></tr>
<tr><td>K.YLGN[+1622.582]ATAIFFLPDEGK.L</td><td>HexNAc(4)Hex(5)</td><td>N[+1623]</td><td>1126.831</td><td>3</td><td>3378.479</td><td>3378.477</td><td>0.7</td><td>Specific</td><td>626.8</td><td>553.1</td><td>553.1</td><td>11.28</td><td>>sp|P01009|A1AT_HUMAN Alpha-1-antitrypsin OS=Homo sapiens GN=SERPINA1 PE=1 SV=3</td></tr>
<tr><td>K.VSN[+1622.582]QTLSLFFTVLQDVPVR.D</td><td>HexNAc(4)Hex(5)</td><td>N[+1623]</td><td>1262.596</td><td>3</td><td>3785.774</td><td>3785.762</td><td>3.1</td><td>Specific</td><td>166.7</td><td>166.7</td><td>166.7</td><td>4.25</td><td>>sp|P01023|A2MG_HUMAN Alpha-2-macroglobulin OS=Homo sapiens GN=A2M</td></tr>
</table>

													PE=1 SV=3
K.TVLTPATN[+0.984]HM[+15.995]GN[+1378.476]VTFTIPANR.E	HexNAc(2)Hex(6)	M[+16], N[+1][+1378]	1217.553	3	3650.644	3650.615	8.1	Specific	124.0	124.0	98.3	2.81	>sp|P01024|CO3_HUMAN Complement C3 OS=Homo sapiens GN=C3 PE=1 SV=2
K.TVLTPATN[+0.984]HMGN[+1378.476]VTFTIPANR.E	HexNAc(2)Hex(6)	N[+1][+1378]	1212.217	3	3634.635	3634.620	4.2	Specific	379.1	379.1	173.9	7.17	>sp|P01024|CO3_HUMAN Complement C3 OS=Homo sapiens GN=C3 PE=1 SV=2
K.TVLTPATN[+0.984]HMGN[+1540.529]VTFTIPANR.E	HexNAc(2)Hex(7)	N[+1][+1541]	1266.237	3	3796.695	3796.673	5.8	Specific	384.1	384.1	201.3	4.89	>sp|P01024|CO3_HUMAN Complement C3 OS=Homo sapiens GN=C3 PE=1 SV=2
K.TVLTPATNHM[+15.995]GN[+1216.423]VTFTIPANR.E	HexNAc(2)Hex(5)	M[+16], N[+1216]	1163.202	3	3487.590	3487.578	3.6	Specific	141.1	117.2	117.2	3.45	>sp|P01024|CO3_HUMAN Complement C3 OS=Homo sapiens GN=C3 PE=1 SV=2
K.TVLTPATNHM[+15.995]GN[+1378.476]VTFTIPANR.E	HexNAc(2)Hex(6)	M[+16], N[+1378]	1217.217	3	3649.636	3649.631	1.5	Specific	362.2	253.6	253.6	7.39	>sp|P01024|CO3_HUMAN Complement C3 OS=Homo sapiens GN=C3 PE=1 SV=2
K.TVLTPATNHMGN[+1378.476]VTFTIPANR.E	HexNAc(2)Hex(6)	N[+1378]	909.165	4	3633.637	3633.636	0.3	Specific	555.7	555.7	293.5	5.09	>sp|P01024|CO3_HUMAN Complement C3 OS=Homo sapiens GN=C3 PE=1 SV=2
K.TVLTPATNHMGN[+1540.529]VTFTIPANR.E	HexNAc(2)Hex(7)	N[+1541]	1265.903	3	3795.696	3795.689	1.9	Specific	497.1	497.1	497.1	5.35	>sp|P01024|CO3_HUMAN Complement C3 OS=Homo sapiens GN=C3 PE=1 SV=2
K.TVLTPATNHMGN[+1864.634]VTFTIPANR.E	HexNAc(2)Hex(9)	N[+1865]	1374.273	3	4120.805	4119.794	1.8	Specific	107.6	107.6	107.6	2.32	>sp|P01024|CO3_HUMAN Complement C3 OS=Homo sapiens GN=C3 PE=1 SV=2
K.VVPEGIRMN[+1702.581]K.T	HexNAc(2)Hex(8)	N[+1703]	949.077	3	2845.216	2845.216	0.0	Specific	142.1	142.1	142.1	1.79	>sp|P01024|CO3_HUMAN Complement C3 OS=Homo sapiens GN=C3 PE=1 SV=2
R.MN[+1702.581]KTVAVR.T	HexNAc(2)Hex(8)	N[+1703]	874.373	3	2621.105	2621.100	1.7	Specific	328.3	316.0	316.0	3.48	>sp|P01024|CO3_HUMAN Complement C3 OS=Homo sapiens GN=C3 PE=1 SV=2
R.MN[+1864.634]KTVAVR.T	HexNAc(2)Hex(9)	N[+1865]	928.391	3	2783.159	2783.153	2.2	Specific	299.3	299.3	299.3	3.13	>sp|P01024|CO3_HUMAN Complement C3 OS=Homo sapiens GN=C3 PE=1 SV=2
R.EEQYN[+1768.640]STYR.V	HexNAc(4)Hex(5)Fuc(1)	N[+1769]	986.725	3	2958.160	2958.152	2.7	Specific	136.7	136.7	136.7	2.80	>sp|P01857|IGHG1_HUMAN Ig gamma-1 chain C region OS=Homo sapiens GN=IGHG1 PE=1 SV=1
R.EEQYN[+1444.534]STYR.V	HexNAc(4)Hex(3)Fuc(1)	N[+1445]	878.689	3	2634.054	2634.046	3.0	Specific	310.5	187.7	187.7	4.47	>sp|P01857|IGHG1_HUMAN Ig gamma-1 chain C region OS=Homo sapiens GN=IGHG1 PE=1 SV=1
R.EEQYN[+1606.587]STYR.V	HexNAc(4)Hex(4)Fuc(1)	N[+1607]	932.707	3	2796.107	2796.099	3.0	Specific	265.0	265.0	265.0	3.90	>sp|P01857|IGHG1_HUMAN Ig gamma-1 chain C region OS=Homo sapiens GN=IGHG1 PE=1 SV=1
R.EEQYN[+1444.534]STYR.V	HexNAc(4)Hex(3)Fuc(1)	N[+1445]	878.689	3	2634.052	2634.046	2.2	Specific	268.2	137.3	137.3	2.53	>sp|P01857|IGHG1_HUMAN Ig gamma-1 chain C region OS=Homo sapiens GN=IGHG1 PE=1 SV=1
R.EEQYN[+1622.582]STYR.V	HexNAc(4)Hex(5)	N[+1623]	938.037	3	2812.097	2812.094	1.1	Specific	168.1	134.0	134.0	2.75	>sp|P01857|IGHG1_HUMAN Ig gamma-1 chain C region OS=Homo sapiens GN=IGHG1 PE=1 SV=1
R.EEQYN[+349.137]STYR.V	HexNAc(1)Fuc(1)	N[+349]	769.832	2	1538.657	1538.649	5.2	Specific	265.2	265.2	265.2	4.18	>sp|P01857|IGHG1_HUMAN Ig gamma-1 chain C region OS=Homo sapiens GN=IGHG1 PE=1

													SV=1
R.EEQYN[+1606.587]STYR.V	HexNAc(4)Hex(4)Fuc(1)	N[+1607]	932.706	3	2796.105	2796.099	2.1	Specific	294.6	294.6	294.6	5.52	>sp|P01857|IGHG1_HUMAN Ig gamma-1 chain C region OS=Homo sapiens GN=IGHG1 PE=1 SV=1
R.EEQFN[+349.137]STFR.V	HexNAc(1)Fuc(1)	N[+349]	753.835	2	1506.663	1506.660	2.6	Specific	196.8	196.8	196.8	2.13	>sp|P01859|IGHG2_HUMAN Ig gamma-2 chain C region OS=Homo sapiens GN=IGHG2 PE=1 SV=2
R.EEQFN[+1241.454]STFR.V	HexNAc(3)Hex(3)Fuc(1)	N[+1241]	800.333	3	2398.985	2398.977	3.5	Specific	265.1	265.1	265.1	5.04	>sp|P01859|IGHG2_HUMAN Ig gamma-2 chain C region OS=Homo sapiens GN=IGHG2 PE=1 SV=2
R.EEQFN[+349.137]STFR.V	HexNAc(1)Fuc(1)	N[+349]	753.837	2	1506.668	1506.660	5.4	Specific	494.9	494.9	494.9	7.23	>sp|P01859|IGHG2_HUMAN Ig gamma-2 chain C region OS=Homo sapiens GN=IGHG2 PE=1 SV=2
K.YKN[+1768.640]NSDISSTR.G	HexNAc(4)Hex(5)Fuc(1)	N[+1769]	1018.426	3	3053.264	3053.257	2.1	Specific	95.2	77.0	77.0	0.14	>sp|P01871|IGHM_HUMAN Ig mu chain C region OS=Homo sapiens GN=IGHM PE=1 SV=3
R.PALEDLLLGSEAN[+1913.677]LTC[+57.021]TLTGLR.D	HexNAc(4)Hex(5)Fuc(0)NeuAc(1)	C[+57], N[+1914]	1068.487	4	4270.924	4270.915	2.1	Specific	265.8	210.0	210.0	6.45	>sp|P01876|IGHA1_HUMAN Ig alpha-1 chain C region OS=Homo sapiens GN=IGHA1 PE=1 SV=2
K.TPLTAN[+1768.640]ITK.S	HexNAc(4)Hex(5)Fuc(1)	N[+1769]	909.739	3	2727.203	2727.196	2.3	Specific	144.4	11.4	11.4	1.89	>sp|P01877|IGHA2_HUMAN Ig alpha-2 chain C region OS=Homo sapiens GN=IGHA2 PE=1 SV=3
K.VDKDLQSLEDILHQVEN[+1913.677]K.T	HexNAc(4)Hex(5)Fuc(0)NeuAc(1)	N[+1914]	1009.950	4	4036.779	4036.775	0.9	Specific	252.8	79.9	79.9	2.76	>sp|P02679-2|FIBG_HUMAN Isoform Gamma-A of Fibrinogen gamma chain OS=Homo sapiens GN=FGG
K.VDKDLQSLEDILHQVEN[+1622.582]K.T	HexNAc(4)Hex(5)	N[+1623]	937.177	4	3745.684	3745.680	1.2	Specific	243.1	32.9	32.9	2.76	>sp|P02679-2|FIBG_HUMAN Isoform Gamma-A of Fibrinogen gamma chain OS=Homo sapiens GN=FGG
K.VDKDLQSLEDILHQVEN[+1622.582]K.T	HexNAc(4)Hex(5)	N[+1623]	937.176	4	3745.682	3745.680	0.6	Specific	461.7	190.2	190.2	5.12	>sp|P02679-2|FIBG_HUMAN Isoform Gamma-A of Fibrinogen gamma chain OS=Homo sapiens GN=FGG
K.DLQSLEDILHQVEN[+1622.582]K.T	HexNAc(4)Hex(5)	N[+1623]	1135.168	3	3403.488	3403.489	-0.4	Specific	179.7	11.8	11.8	2.49	>sp|P02679-2|FIBG_HUMAN Isoform Gamma-A of Fibrinogen gamma chain OS=Homo sapiens GN=FGG
R.ESVTDHVNLITPLEKPLQN[+1913.677]FTLC[+57.021]FR.A	HexNAc(4)Hex(5)Fuc(0)NeuAc(1)	C[+57], N[+1914]	1222.312	4	4886.226	4885.212	2.2	Specific	125.5	65.4	65.4	3.05	>sp|P02743|SAMP_HUMAN Serum amyloid P-component OS=Homo sapiens GN=APCS PE=1 SV=2
K.PLQN[+2204.772]FTLC[+57.021]FR.A	HexNAc(4)Hex(5)Fuc(0)NeuAc(2	C[+57],	1167.481	3	3500.428	3500.429	-0.2	Specific	199.1	199.1	199.1	2.61	>sp|P02743|SAMP_HUMAN Serum amyloid P-

	)	N[+2205]											component OS=Homo sapiens GN=APCS PE=1 SV=2
R.VYKPSAGN[+2204.772]NSLYR.D	HexNAc(4)Hex(5)Fuc(0)NeuAc(2)	N[+2205]	1225.184	3	3673.537	3673.527	2.8	Specific	141.3	45.3	45.3	2.25	>sp|P02749|APOH_HUMAN Beta-2-glycoprotein 1 OS=Homo sapiens GN=APOH PE=1 SV=3
R.VYKPSAGN[+1768.640]NSLYR.D	HexNAc(4)Hex(5)Fuc(1)	N[+1769]	1079.807	3	3237.405	3237.394	3.6	Specific	174.8	3.1	3.1	0.84	>sp|P02749|APOH_HUMAN Beta-2-glycoprotein 1 OS=Homo sapiens GN=APOH PE=1 SV=3
R.VYKPSAGN[+1622.582]NSLYR.D	HexNAc(4)Hex(5)	N[+1623]	1031.120	3	3091.345	3091.336	2.8	Specific	201.9	32.4	32.4	2.55	>sp|P02749|APOH_HUMAN Beta-2-glycoprotein 1 OS=Homo sapiens GN=APOH PE=1 SV=3
K.AALAAFNAQN[+0.984]N[+1622.582]GSNFQLEEISR.A	HexNAc(4)Hex(5)	N[+1][+1623]	1330.250	3	3988.735	3988.719	4.1	Specific	257.2	257.2	3.9	6.29	>sp|P02765|FETUA_HUMAN Alpha-2-HS-glycoprotein OS=Homo sapiens GN=AHSG PE=1 SV=1
R.TAGWNIPMGLLFN[+2204.772]QTGSC[+57.021]K.F	HexNAc(4)Hex(5)Fuc(0)NeuAc(2)	C[+57], N[+2205]	1075.959	4	4300.813	4299.782	6.3	Specific	317.4	248.0	248.0	3.22	>sp|P02788-2|TRFL_HUMAN Isoform DeltaLf of Lactotransferrin OS=Homo sapiens GN=LTF
K.ALPQPQN[+2204.772]VTSLLGC[+57.021]TH.-	HexNAc(4)Hex(5)Fuc(0)NeuAc(2)	C[+57], N[+2205]	1314.224	3	3940.656	3940.652	1.1	CRagged	156.8	131.0	131.0	3.38	>sp|P02790|HEMO_HUMAN Hemopexin OS=Homo sapiens GN=HPX PE=1 SV=2
K.AFQLWSN[+203.079]VTPLTFTK.V	HexNAc(1)	N[+203]	978.511	2	1956.015	1956.011	1.9	Specific	122.2	27.5	27.5	0.05	>sp|P03956|MMP1_HUMAN Interstitial collagenase OS=Homo sapiens GN=MMP1 PE=1 SV=3
R.N[+2204.772]ISDGFDGIPDNVDAALALPAHSYSGR.E	HexNAc(4)Hex(5)Fuc(0)NeuAc(2)	N[+2205]	1245.033	4	4977.110	4977.095	3.0	Specific	253.8	219.9	113.5	0.74	>sp|P04004|VTNC_HUMAN Vitronectin OS=Homo sapiens GN=VTN PE=1 SV=1
K.NN[+2204.772]ATVHEQVGGPSLTSDLQAQSK.G	HexNAc(4)Hex(5)Fuc(0)NeuAc(2)	N[+2205]	1147.492	4	4586.948	4585.942	0.6	Specific	132.6	15.0	15.0	0.90	>sp|P04004|VTNC_HUMAN Vitronectin OS=Homo sapiens GN=VTN PE=1 SV=1
K.N[+2204.772]GSLFAFR.G	HexNAc(4)Hex(5)Fuc(0)NeuAc(2)	N[+2205]	1039.423	3	3116.253	3116.246	2.4	Specific	147.7	101.7	101.7	1.22	>sp|P04004|VTNC_HUMAN Vitronectin OS=Homo sapiens GN=VTN PE=1 SV=1
R.N[+2204.772]ISDGFDGIPDNVDAALALPAHSYSGR.E	HexNAc(4)Hex(5)Fuc(0)NeuAc(2)	N[+2205]	830.524	6	4978.108	4977.095	1.9	Specific	353.2	353.2	161.6	5.29	>sp|P04004|VTNC_HUMAN Vitronectin OS=Homo sapiens GN=VTN PE=1 SV=1
K.N[+1419.502]GSLFAFR.G	HexNAc(3)Hex(5)	N[+1420]	1165.992	2	2330.978	2330.976	0.9	Specific	105.9	20.2	20.2	2.13	>sp|P04004|VTNC_HUMAN Vitronectin OS=Homo sapiens GN=VTN PE=1 SV=1
R.N[+1622.582]ISDGFDGIPDNVDAALALPAHSYSGR.E	HexNAc(4)Hex(5)	N[+1623]	1099.733	4	4395.911	4394.904	0.9	Specific	131.3	131.3	131.3	2.17	>sp|P04004|VTNC_HUMAN Vitronectin OS=Homo sapiens GN=VTN PE=1 SV=1
K.N[+1913.677]GSLFAFR.G	HexNAc(4)Hex(5)Fuc(0)NeuAc(1)	N[+1914]	942.390	3	2825.155	2825.150	1.7	Specific	53.6	53.6	53.6	0.72	>sp|P04004|VTNC_HUMAN Vitronectin OS=Homo sapiens GN=VTN PE=1 SV=1
K.N[+1622.582]GSLFAFR.G	HexNAc(4)Hex(5)	N[+1623]	845.356	3	2534.054	2534.055	-0.4	Specific	147.9	134.6	134.6	0.67	>sp|P04004|VTNC_HUMAN Vitronectin OS=Homo sapiens GN=VTN PE=1 SV=1
R.N[+1622.582]ISDGFDGIPDNVDAALALPAHSYSGR.E	HexNAc(4)Hex(5)	N[+1623]	1099.484	4	4394.913	4394.904	2.0	Specific	292.9	274.3	105.6	6.88	>sp|P04004|VTNC_HUMAN Vitronectin OS=Homo sapiens GN=VTN PE=1 SV=1

K.YLQPLLAVQFTN[+1768.640]LTM[+15.995]DTEIR.I	HexNAc(4)Hex(5)Fuc(1)	M[+16], N[+1769]	1384.634	3	4151.886	4150.877	1.4	Specific	88.1	30.2	30.2	0.30	>sp|P05026|AT1B1_HUMAN Sodium/potassium-transporting ATPase subunit beta-1 OS=Homo sapiens GN=ATP1B1 PE=1 SV=1
K.YLQPLLAVQFTN[+2350.830]LTM[+15.995]DTEIR.I	HexNAc(4)Hex(5)Fuc(1)NeuAc(2)	M[+16], N[+2351]	1184.275	4	4734.079	4733.068	1.7	Specific	121.2	7.4	7.4	0.30	>sp|P05026|AT1B1_HUMAN Sodium/potassium-transporting ATPase subunit beta-1 OS=Homo sapiens GN=ATP1B1 PE=1 SV=1
R.SYN[+1038.375]DSVDPR.I	HexNAc(2)Hex(3)Fuc(1)	N[+1038]	1045.926	2	2090.845	2090.839	2.8	Specific	169.3	86.4	86.4	0.42	>sp|P05164|PERM_HUMAN Myeloperoxidase OS=Homo sapiens GN=MPO PE=1 SV=1
K.GSLSYLN[+892.317]VTR.K	HexNAc(2)Hex(3)	N[+892]	1001.462	2	2001.917	2001.912	2.3	Specific	102.1	32.6	32.6	2.03	>sp|P07339|CATD_HUMAN Cathepsin D OS=Homo sapiens GN=CTSD PE=1 SV=1
K.GSLSYLN[+1378.476]VTR.K	HexNAc(2)Hex(6)	N[+1378]	1244.543	2	2488.078	2488.071	2.9	Specific	127.4	78.8	78.8	0.59	>sp|P07339|CATD_HUMAN Cathepsin D OS=Homo sapiens GN=CTSD PE=1 SV=1
K.GSLSYLN[+1054.370]VTR.K	HexNAc(2)Hex(4)	N[+1054]	1082.487	2	2163.967	2163.965	0.9	Specific	130.2	89.2	89.2	0.61	>sp|P07339|CATD_HUMAN Cathepsin D OS=Homo sapiens GN=CTSD PE=1 SV=1
K.GSLSYLN[+1216.423]VTR.K	HexNAc(2)Hex(5)	N[+1216]	1163.515	2	2326.023	2326.018	2.1	Specific	134.0	78.4	78.4	0.59	>sp|P07339|CATD_HUMAN Cathepsin D OS=Homo sapiens GN=CTSD PE=1 SV=1
R.IADTN[+1913.677]ITSIPQGLPPSLTELHLDGNK.I	HexNAc(4)Hex(5)Fuc(0)NeuAc(1)	N[+1914]	1165.290	4	4658.137	4658.124	2.9	Specific	126.1	112.5	112.5	3.31	>sp|P07585|PGS2_HUMAN Decorin OS=Homo sapiens GN=DCN PE=1 SV=1
K.LGLSFNSISAVDN[+2350.830]GSLANTPHLR.E	HexNAc(4)Hex(5)Fuc(1)NeuAc(2)	N[+2351]	1184.525	4	4735.077	4734.067	1.4	Specific	113.9	19.5	19.5	2.93	>sp|P07585|PGS2_HUMAN Decorin OS=Homo sapiens GN=DCN PE=1 SV=1
K.LGLSFNSISAVDN[+2059.735]GSLANTPHLR.E	HexNAc(4)Hex(5)Fuc(1)NeuAc(1)	N[+2060]	1111.751	4	4443.981	4442.971	1.3	Specific	396.3	247.8	94.7	7.46	>sp|P07585|PGS2_HUMAN Decorin OS=Homo sapiens GN=DCN PE=1 SV=1
K.LGLSFNSISAVDN[+1768.640]GSLANTPHLR.E	HexNAc(4)Hex(5)Fuc(1)	N[+1769]	1038.726	4	4151.884	4151.876	1.8	Specific	165.8	148.7	41.6	2.90	>sp|P07585|PGS2_HUMAN Decorin OS=Homo sapiens GN=DCN PE=1 SV=1
K.LGLSFN[+0.984]SISAVDN[+2059.735]GSLANTPHLR.E	HexNAc(4)Hex(5)Fuc(1)NeuAc(1)	N[+1][+2060]	1111.744	4	4443.955	4443.955	0.0	Specific	244.7	143.8	101.7	3.19	>sp|P07585|PGS2_HUMAN Decorin OS=Homo sapiens GN=DCN PE=1 SV=1
R.IADTN[+1419.502]ITSIPQGLPPSLTELHLDGNK.I	HexNAc(3)Hex(5)	N[+1420]	1041.743	4	4163.950	4163.949	0.4	Specific	266.0	175.7	175.7	3.93	>sp|P07585|PGS2_HUMAN Decorin OS=Homo sapiens GN=DCN PE=1 SV=1
R.IADTN[+1257.449]ITSIPQGLPPSLTELHLDGNK.I	HexNAc(3)Hex(4)	N[+1257]	1001.231	4	4001.902	4001.896	1.5	Specific	296.2	218.3	218.3	4.49	>sp|P07585|PGS2_HUMAN Decorin OS=Homo sapiens GN=DCN PE=1 SV=1
R.IADTN[+1622.582]ITSIPQGLPPSLTELHLDGNK.I	HexNAc(4)Hex(5)	N[+1623]	1092.514	4	4367.032	4367.028	1.0	Specific	396.0	253.5	253.5	5.55	>sp|P07585|PGS2_HUMAN Decorin OS=Homo sapiens GN=DCN PE=1 SV=1
K.LGLSFNSISAVDN[+2059.735]GSLANTPHLR.E	HexNAc(4)Hex(5)Fuc(1)NeuAc(1)	N[+2060]	1111.501	4	4442.983	4442.971	2.6	Specific	292.4	167.0	107.7	4.18	>sp|P07585|PGS2_HUMAN Decorin OS=Homo sapiens GN=DCN PE=1 SV=1
K.LGLSFNSISAVDN[+1768.640]GSLANTPHLR.E	HexNAc(4)Hex(5)Fuc(1)	N[+1769]	1038.726	4	4151.884	4151.876	1.8	Specific	493.3	305.5	177.0	6.54	>sp|P07585|PGS2_HUMAN Decorin OS=Homo sapiens GN=DCN PE=1 SV=1

K.LGLSFN[+0.984]SISAVDN[+1768.640]GSLANTPHLR.E	HexNAc(4)Hex(5)Fuc(1)	N[+1][+1769]	1038.972	4	4152.866	4152.860	1.4	Specific	517.4	337.9	177.2	6.88	>sp|P07585|PGS2_HUMAN Decorin OS=Homo sapiens GN=DCN PE=1 SV=1
K.VVN[+1864.634]STTGPGEHLR.N	HexNAc(2)Hex(9)	N[+1865]	1077.784	3	3231.338	3231.342	-1.1	Specific	229.5	229.5	229.5	0.78	>sp|P07996|TSP1_HUMAN Thrombospondin-1 OS=Homo sapiens GN=THBS1 PE=1 SV=2
K.VVN[+1622.582]STTGPGEHLR.N	HexNAc(4)Hex(5)	N[+1623]	997.102	3	2989.293	2989.289	1.2	Specific	242.2	113.0	113.0	0.92	>sp|P07996|TSP1_HUMAN Thrombospondin-1 OS=Homo sapiens GN=THBS1 PE=1 SV=2
K.VVN[+1913.677]STTGPGEHLR.N	HexNAc(4)Hex(5)Fuc(0)NeuAc(1)	N[+1914]	1094.137	3	3280.397	3280.384	3.8	Specific	127.3	127.3	127.3	2.26	>sp|P07996|TSP1_HUMAN Thrombospondin-1 OS=Homo sapiens GN=THBS1 PE=1 SV=2
K.SC[+57.021]HTGIN[+2204.772]R.T	HexNAc(4)Hex(5)Fuc(0)NeuAc(2)	C[+57], N[+2205]	1050.417	3	3149.236	3149.209	8.4	Specific	178.0	132.4	132.4	0.60	>sp|P08582|TRFM_HUMAN Melanotransferrin OS=Homo sapiens GN=MFI2 PE=1 SV=2
R.GLN[+1622.582]VTLSSTGR.N	HexNAc(4)Hex(5)	N[+1623]	909.734	3	2727.188	2727.182	1.9	Specific	160.2	159.5	159.5	2.29	>sp|P0C0L5|CO4B_HUMAN Complement C4-B OS=Homo sapiens GN=C4B PE=1 SV=2
R.LAN[+1622.582]LTQGEDQYYLR.V	HexNAc(4)Hex(5)	N[+1623]	1102.806	3	3306.404	3306.415	-3.4	Specific	225.5	182.5	182.5	2.59	>sp|P10909-3|CLUS_HUMAN Isoform 3 of Clusterin OS=Homo sapiens GN=CLU
R.RVN[+0.984]DN[+1540.529]KTAAEEALR.K	HexNAc(2)Hex(7)	N[+1][+1541]	782.841	4	3128.343	3128.337	2.0	Specific	200.0	160.9	160.9	0.72	>sp|P11047|LAMC1_HUMAN Laminin subunit gamma-1 OS=Homo sapiens GN=LAMC1 PE=1 SV=3
K.TAN[+1540.529]DTSTEAYNLLLR.T	HexNAc(2)Hex(7)	N[+1541]	1074.797	3	3222.377	3222.368	3.0	Specific	249.2	156.4	156.4	2.99	>sp|P11047|LAMC1_HUMAN Laminin subunit gamma-1 OS=Homo sapiens GN=LAMC1 PE=1 SV=3
K.TAN[+1216.423]DTSTEAYNLLLR.T	HexNAc(2)Hex(5)	N[+1216]	966.761	3	2898.269	2898.262	2.4	Specific	743.2	529.5	529.5	10.34	>sp|P11047|LAMC1_HUMAN Laminin subunit gamma-1 OS=Homo sapiens GN=LAMC1 PE=1 SV=3
K.LLNN[+1216.423]LTSIK.I	HexNAc(2)Hex(5)	N[+1216]	1116.524	2	2232.040	2232.038	1.3	Specific	98.8	98.8	98.8	0.38	>sp|P11047|LAMC1_HUMAN Laminin subunit gamma-1 OS=Homo sapiens GN=LAMC1 PE=1 SV=3
K.LLNN[+1378.476]LTSIK.I	HexNAc(2)Hex(6)	N[+1378]	1197.552	2	2394.096	2394.090	2.3	Specific	84.4	84.4	84.4	0.23	>sp|P11047|LAMC1_HUMAN Laminin subunit gamma-1 OS=Homo sapiens GN=LAMC1 PE=1 SV=3
K.LLNN[+1216.423]LTSIK.I	HexNAc(2)Hex(5)	N[+1216]	1116.524	2	2232.041	2232.038	1.4	Specific	110.9	110.9	110.9	0.61	>sp|P11047|LAMC1_HUMAN Laminin subunit gamma-1 OS=Homo sapiens GN=LAMC1 PE=1 SV=3
R.GHTLTLN[+1378.476]FTR.N	HexNAc(2)Hex(6)	N[+1378]	846.706	3	2538.104	2538.098	2.6	Specific	252.7	27.5	27.5	2.97	>sp|P11279|LAMP1_HUMAN Lysosome-associated membrane glycoprotein 1 OS=Homo sapiens GN=LAMP1 PE=1 SV=3

R.GHTLTLN[+1378.476]FTR.N	HexNAc(2)Hex(6)	N[+1378]	846.706	3	2538.104	2538.098	2.5	Specific	213.4	47.0	47.0	2.41	>sp|P11279|LAMP1_HUMAN Lysosome-associated membrane glycoprotein 1 OS=Homo sapiens GN=LAMP1 PE=1 SV=3
R.GTFTDC[+57.021]ALAN[+1622.582]MTEQIR.Q	HexNAc(4)Hex(5)	C[+57], N[+1623]	1150.813	3	3450.426	3450.418	2.2	Specific	191.3	162.6	162.6	3.58	>sp|P12110|CO6A2_HUMAN Collagen alpha-2(VI) chain OS=Homo sapiens GN=COL6A2 PE=1 SV=4
R.N[+1768.640]FTAADWGQSR.D	HexNAc(4)Hex(5)Fuc(1)	N[+1769]	1007.742	3	3021.212	3021.210	0.8	Specific	316.8	305.5	305.5	5.80	>sp|P12109|CO6A1_HUMAN Collagen alpha-1(VI) chain OS=Homo sapiens GN=COL6A1 PE=1 SV=3
R.N[+1768.640]M[+15.995]TLFSDLVAEK.F	HexNAc(4)Hex(5)Fuc(1)	M[+16], N[+1769]	1051.448	3	3152.328	3152.322	1.9	Specific	102.2	38.4	38.4	2.14	>sp|P12110|CO6A2_HUMAN Collagen alpha-2(VI) chain OS=Homo sapiens GN=COL6A2 PE=1 SV=4
R.N[+1768.640]MTLFSDLVAEK.F	HexNAc(4)Hex(5)Fuc(1)	N[+1769]	1046.116	3	3136.334	3136.327	2.4	Specific	225.4	132.3	132.3	2.87	>sp|P12110|CO6A2_HUMAN Collagen alpha-2(VI) chain OS=Homo sapiens GN=COL6A2 PE=1 SV=4
R.N[+2059.735]FTAADWGQSR.D	HexNAc(4)Hex(5)Fuc(1)NeuAc(1)	N[+2060]	1104.773	3	3312.306	3312.305	0.1	Specific	157.1	152.8	152.8	2.35	>sp|P12109|CO6A1_HUMAN Collagen alpha-1(VI) chain OS=Homo sapiens GN=COL6A1 PE=1 SV=3
R.N[+2059.735]M[+15.995]TLFSDLVAEK.F	HexNAc(4)Hex(5)Fuc(1)NeuAc(1)	M[+16], N[+2060]	1148.478	3	3443.419	3443.417	0.4	Specific	122.5	106.3	106.3	2.88	>sp|P12110|CO6A2_HUMAN Collagen alpha-2(VI) chain OS=Homo sapiens GN=COL6A2 PE=1 SV=4
R.N[+2059.735]MTLFSDLVAEK.F	HexNAc(4)Hex(5)Fuc(1)NeuAc(1)	N[+2060]	1143.148	3	3427.430	3427.422	2.3	Specific	154.5	104.7	104.7	2.84	>sp|P12110|CO6A2_HUMAN Collagen alpha-2(VI) chain OS=Homo sapiens GN=COL6A2 PE=1 SV=4
R.N[+2350.830]FTAADWGQSR.D	HexNAc(4)Hex(5)Fuc(1)NeuAc(2)	N[+2351]	1201.806	3	3603.404	3603.401	0.9	Specific	109.0	109.0	109.0	2.31	>sp|P12109|CO6A1_HUMAN Collagen alpha-1(VI) chain OS=Homo sapiens GN=COL6A1 PE=1 SV=3
R.N[+2350.830]M[+15.995]TLFSDLVAEK.F	HexNAc(4)Hex(5)Fuc(1)NeuAc(2)	M[+16], N[+2351]	1245.512	3	3734.521	3734.513	2.1	Specific	128.6	69.0	59.2	2.98	>sp|P12110|CO6A2_HUMAN Collagen alpha-2(VI) chain OS=Homo sapiens GN=COL6A2 PE=1 SV=4
R.RN[+892.317]FTAADWGQSR.D	HexNAc(2)Hex(3)	N[+892]	767.669	3	2300.993	2300.989	1.7	Specific	30.0	0.3	0.3	0.07	>sp|P12109|CO6A1_HUMAN Collagen alpha-1(VI) chain OS=Homo sapiens GN=COL6A1 PE=1 SV=3
R.QLINALQIN[+1768.640]NTAVGHALVLPAGR.D	HexNAc(4)Hex(5)Fuc(1)	N[+1769]	1038.756	4	4152.002	4151.996	1.3	Specific	590.4	474.1	159.6	9.51	>sp|P12111-2|CO6A3_HUMAN Isoform 2 of Collagen alpha-3(VI) chain OS=Homo sapiens GN=COL6A3
R.QLINALQIN[+1768.640]NTAVGHALVLPAGR.D	HexNAc(4)Hex(5)Fuc(1)	N[+1769]	1384.674	3	4152.007	4151.996	2.5	Specific	204.3	178.1	131.9	4.39	>sp|P12111|CO6A3_HUMAN Collagen alpha-3(VI) chain OS=Homo sapiens GN=COL6A3 PE=1 SV=5
R.QLINALQIN[+2059.735]NTAVGHALVLPAGR.D	HexNAc(4)Hex(5)Fuc(1)NeuAc(1	N[+2060]	1111.528	4	4443.091	4443.092	-0.2	Specific	312.9	312.9	67.9	6.57	>sp|P12111-2|CO6A3_HUMAN Isoform 2 of

	)												Collagen alpha-3(VI) chain OS=Homo sapiens GN=COL6A3
R.QLINALQIN[+2350.830]NTAVGHALVLPAGR.D	HexNAc(4)Hex(5)Fuc(1)NeuAc(2)	N[+2351]	1184.303	4	4734.190	4734.187	0.6	Specific	249.2	204.1	52.4	5.30	>sp|P12111-2|CO6A3_HUMAN Isoform 2 of Collagen alpha-3(VI) chain OS=Homo sapiens GN=COL6A3
R.QLINALQIN[+2372.812]N[+0.984]TAVGHALVLPAGR.D	HexNAc(4)Hex(5)Fuc(1)NeuAc(2)NeuGc(0)Na(1)	N[+1][+2373]	952.437	5	4758.155	4757.153	-0.2	Specific	110.6	110.6	38.7	3.15	>sp|P12111-2|CO6A3_HUMAN Isoform 2 of Collagen alpha-3(VI) chain OS=Homo sapiens GN=COL6A3
R.QLINALQIN[+349.137]NTAVGHALVLPAGR.D	HexNAc(1)Fuc(1)	N[+349]	911.504	3	2732.499	2732.494	1.7	Specific	400.8	400.8	400.8	9.27	>sp|P12111-2|CO6A3_HUMAN Isoform 2 of Collagen alpha-3(VI) chain OS=Homo sapiens GN=COL6A3
R.RN[+1216.423]N[+0.984]TFLSLR.D	HexNAc(2)Hex(5)	N[+1][+1216]	780.015	3	2338.029	2338.029	0.0	Specific	236.6	31.6	31.6	0.73	>sp|P13726|TF_HUMAN Tissue factor OS=Homo sapiens GN=F3 PE=1 SV=1
R.RN[+1378.476]N[+0.984]TFLSLR.D	HexNAc(2)Hex(6)	N[+1][+1378]	834.033	3	2500.086	2500.082	1.5	Specific	244.2	233.3	233.3	1.26	>sp|P13726|TF_HUMAN Tissue factor OS=Homo sapiens GN=F3 PE=1 SV=1
K.HN[+0.984]N[+1216.423]DTQHIWESDSNEFSVIADPR.G	HexNAc(2)Hex(5)	N[+1][+1216]	982.919	4	3928.654	3928.615	9.8	Specific	78.0	78.0	12.6	1.42	>sp|P14625|ENPL_HUMAN Endoplasmin OS=Homo sapiens GN=HSP90B1 PE=1 SV=1
K.HN[+0.984]N[+1378.476]DTQHIWESDSNEFSVIADPR.G	HexNAc(2)Hex(6)	N[+1][+1378]	1023.678	4	4091.690	4090.668	4.6	Specific	187.1	187.1	0.0	2.79	>sp|P14625|ENPL_HUMAN Endoplasmin OS=Homo sapiens GN=HSP90B1 PE=1 SV=1
K.HNN[+1378.476]DTQHIWESDSNEFSVIADPR.G	HexNAc(2)Hex(6)	N[+1378]	1023.178	4	4089.691	4089.684	1.9	Specific	340.4	268.6	111.8	6.41	>sp|P14625|ENPL_HUMAN Endoplasmin OS=Homo sapiens GN=HSP90B1 PE=1 SV=1
K.HNN[+1702.581]DTQHIWESDSNEFSVIADPR.G	HexNAc(2)Hex(8)	N[+1703]	1104.458	4	4414.810	4413.789	4.0	Specific	353.4	179.7	179.7	4.19	>sp|P14625|ENPL_HUMAN Endoplasmin OS=Homo sapiens GN=HSP90B1 PE=1 SV=1
R.GASN[+1378.476]LTWR.S	HexNAc(2)Hex(6)	N[+1378]	1141.975	2	2282.943	2282.939	1.5	Specific	143.6	108.8	108.8	1.18	>sp|P15586|GNS_HUMAN N-acetylglucosamine-6-sulfatase OS=Homo sapiens GN=GNS PE=1 SV=3
R.GPGIKPN[+1378.476]QTSK.M	HexNAc(2)Hex(6)	N[+1378]	835.706	3	2505.104	2505.097	2.6	Specific	220.3	220.3	220.3	2.32	>sp|P15586|GNS_HUMAN N-acetylglucosamine-6-sulfatase OS=Homo sapiens GN=GNS PE=1 SV=3
R.GPGIKPN[+1540.529]QTSK.M	HexNAc(2)Hex(7)	N[+1541]	889.724	3	2667.157	2667.150	2.7	Specific	315.4	315.4	315.4	3.41	>sp|P15586|GNS_HUMAN N-acetylglucosamine-6-sulfatase OS=Homo sapiens GN=GNS PE=1 SV=3
K.AFN[+2350.830]STLPTMAQMEK.A	HexNAc(4)Hex(5)Fuc(1)NeuAc(2)	N[+2351]	1307.201	3	3919.588	3919.575	3.2	Specific	261.2	110.4	110.4	1.39	>sp|P16070|CD44_HUMAN CD44 antigen OS=Homo sapiens GN=CD44 PE=1 SV=3
R.PLQALLDGRGLC[+57.021]VN[+2059.735]ASAVSRLR.A	HexNAc(4)Hex(5)Fuc(1)NeuAc(1)	C[+57], N[+2060]	1107.273	4	4426.069	4426.043	5.8	Specific	111.0	35.1	35.1	0.05	>sp|P17936|IBP3_HUMAN Insulin-like growth factor-binding protein 3 OS=Homo sapiens

													GN=IGFBP3 PE=1 SV=2
R.M[+15.995]IEN[+1768.640]GSLSFLPTLR.E	HexNAc(4)Hex(5)Fuc(1)	M[+16], N[+1769]	1121.495	3	3362.471	3362.470	0.2	Specific	142.0	81.8	81.8	2.12	>sp|P21810|PGS1_HUMAN Biglycan OS=Homo sapiens GN=BGN PE=1 SV=2
K.LLQVVYLHSNN[+1768.640]ITK.V	HexNAc(4)Hex(5)Fuc(1)	N[+1769]	1137.539	3	3410.603	3410.572	9.2	Specific	209.1	44.6	44.6	2.30	>sp|P21810|PGS1_HUMAN Biglycan OS=Homo sapiens GN=BGN PE=1 SV=2
R.AN[+1378.476]HSGAVVLLK.R	HexNAc(2)Hex(6)	N[+1378]	829.716	3	2487.132	2487.123	3.7	Specific	284.8	284.8	284.8	4.28	>sp|P23229-5|ITA6_HUMAN Isoform Alpha-6X2B of Integrin alpha-6 OS=Homo sapiens GN=ITGA6
R.AN[+1540.529]HSGAVVLLKR.D	HexNAc(2)Hex(7)	N[+1541]	935.765	3	2805.279	2805.277	0.7	Specific	178.3	178.3	178.3	0.13	>sp|P23229|ITA6_HUMAN Integrin alpha-6 OS=Homo sapiens GN=ITGA6 PE=1 SV=5
R.AN[+1378.476]HSGAVVLLKR.D	HexNAc(2)Hex(6)	N[+1378]	881.747	3	2643.227	2643.224	0.9	Specific	210.5	210.5	210.5	0.13	>sp|P23229|ITA6_HUMAN Integrin alpha-6 OS=Homo sapiens GN=ITGA6 PE=1 SV=5
R.QTGLAPGQEYEISLHIVKN[+1038.375]NTR.G	HexNAc(2)Hex(3)Fuc(1)	N[+1038]	877.416	4	3506.644	3506.664	-5.9	Specific	76.2	76.2	76.2	0.15	>sp|P24821-4|TENA_HUMAN Isoform 4 of Tenascin OS=Homo sapiens GN=TNC
R.N[+1768.640]TTSYVLR.G	HexNAc(4)Hex(5)Fuc(1)	N[+1769]	908.057	3	2722.156	2722.145	4.2	Specific	96.6	96.6	96.6	0.18	>sp|P24821-4|TENA_HUMAN Isoform 4 of Tenascin OS=Homo sapiens GN=TNC
R.N[+1768.640]LTVPGSLR.A	HexNAc(4)Hex(5)Fuc(1)	N[+1769]	909.070	3	2725.195	2725.192	1.2	Specific	175.4	175.4	175.4	3.20	>sp|P24821-4|TENA_HUMAN Isoform 4 of Tenascin OS=Homo sapiens GN=TNC
R.LN[+1216.423]YSLPTGQWVGVQLPR.N	HexNAc(2)Hex(5)	N[+1216]	1048.826	3	3144.463	3144.462	0.4	Specific	577.0	577.0	577.0	9.71	>sp|P24821|TENA_HUMAN Tenascin OS=Homo sapiens GN=TNC PE=1 SV=3
K.IFLEN[+0.984]GEN[+2204.772]ITTPK.F	HexNAc(4)Hex(5)Fuc(0)NeuAc(2)	N[+1][+2205]	1227.853	3	3681.545	3681.531	4.0	Specific	160.8	160.8	160.8	1.26	>sp|P25092|GUC2C_HUMAN Heat-stable enterotoxin receptor OS=Homo sapiens GN=GUCY2C PE=1 SV=2
R.GYYN[+1864.634]QSEAGSHTLQR.M	HexNAc(2)Hex(9)	N[+1865]	1192.480	3	3575.425	3575.417	2.3	Specific	216.1	107.7	107.7	0.75	>sp|P30505|1C08_HUMAN HLA class I histocompatibility antigen, Cw-8 alpha chain OS=Homo sapiens GN=HLA-C PE=1 SV=1
R.C[+57.021]DSGFALDSEERN[+1216.423]C[+57.021]TDIDEC[+57.021]R.I	HexNAc(2)Hex(5)	C[+57]*3, N[+1216]	1255.816	3	3765.433	3765.420	3.5	Specific	101.3	101.3	101.3	2.33	>sp|P35555|FBN1_HUMAN Fibrillin-1 OS=Homo sapiens GN=FBN1 PE=1 SV=3
R.YPQDYQFYIQN[+1702.581]FTALPLNTVVPPQR.Q	HexNAc(2)Hex(8)	N[+1703]	1179.538	4	4715.131	4715.107	5.0	Specific	309.3	309.3	222.5	3.85	>sp|P43307|SSRA_HUMAN Translocon-associated protein subunit alpha OS=Homo sapiens GN=SSR1 PE=1 SV=3

R.YPQDYQFYIQN[+1864.634]FTALPLNTVVPPQR.Q	HexNAc(2)Hex(9)	N[+1865]	1220.050	4	4877.179	4877.160	4.0	Specific	479.0	479.0	303.9	6.66	>sp|P43307|SSRA_HUMAN Translocon-associated protein subunit alpha OS=Homo sapiens GN=SSR1 PE=1 SV=3
R.TILVDNNTWN[+1864.634]NTHISR.V	HexNAc(2)Hex(9)	N[+1865]	1254.869	3	3762.591	3762.586	1.4	Specific	375.7	240.5	9.4	4.74	>sp|P46977-2|STT3A_HUMAN Isoform 2 of Dolichyl-diphosphooligosaccharide--protein glycosyltransferase subunit STT3A OS=Homo sapiens GN=STT3A
K.VMSWWDYGYQITAMAN[+1864.634]R.T	HexNAc(2)Hex(9)	N[+1865]	1319.532	3	3956.581	3956.576	1.3	Specific	382.6	382.6	382.6	4.02	>sp|P46977|STT3A_HUMAN Dolichyl-diphosphooligosaccharide--protein glycosyltransferase subunit STT3A OS=Homo sapiens GN=STT3A PE=1 SV=2
R.TILVDNNTWN[+1864.634]NTHISR.V	HexNAc(2)Hex(9)	N[+1865]	1254.873	3	3762.604	3762.586	4.8	Specific	228.2	117.5	9.4	1.32	>sp|P46977|STT3A_HUMAN Dolichyl-diphosphooligosaccharide--protein glycosyltransferase subunit STT3A OS=Homo sapiens GN=STT3A PE=1 SV=2
K.DEN[+1241.454]SSKSTFSFSMTK.P	HexNAc(3)Hex(3)Fuc(1)	N[+1241]	735.063	4	2937.228	2937.208	7.1	Specific	132.8	20.8	20.8	1.16	>sp|P49790|NU153_HUMAN Nuclear pore complex protein Nup153 OS=Homo sapiens GN=NUP153 PE=1 SV=2
K.DEN[+1460.529]SSKSTFSFSMTK.P	HexNAc(4)Hex(4)	N[+1461]	789.831	4	3156.301	3156.282	6.0	Specific	168.0	168.0	168.0	1.97	>sp|P49790|NU153_HUMAN Nuclear pore complex protein Nup153 OS=Homo sapiens GN=NUP153 PE=1 SV=2
K.DEN[+2059.735]SSKSTFSFSMTK.P	HexNAc(4)Hex(5)Fuc(1)NeuAc(1)	N[+2060]	939.632	4	3755.506	3755.488	4.7	Specific	217.1	217.1	217.1	1.30	>sp|P49790|NU153_HUMAN Nuclear pore complex protein Nup153 OS=Homo sapiens GN=NUP153 PE=1 SV=2
K.DEN[+1444.534]SSKSTFSFSMTK.P	HexNAc(4)Hex(3)Fuc(1)	N[+1445]	785.832	4	3140.305	3140.287	5.9	Specific	221.9	221.9	221.9	2.26	>sp|P49790|NU153_HUMAN Nuclear pore complex protein Nup153 OS=Homo sapiens GN=NUP153 PE=1 SV=2
K.DEN[+1606.587]SSKSTFSFSMTK.P	HexNAc(4)Hex(4)Fuc(1)	N[+1607]	826.345	4	3302.357	3302.340	5.1	Specific	242.1	242.1	242.1	2.61	>sp|P49790|NU153_HUMAN Nuclear pore complex protein Nup153 OS=Homo sapiens GN=NUP153 PE=1 SV=2
K.DEN[+1768.640]SSKSTFSFSMTK.P	HexNAc(4)Hex(5)Fuc(1)	N[+1769]	866.859	4	3464.413	3464.393	5.9	Specific	243.8	243.8	243.8	2.86	>sp|P49790|NU153_HUMAN Nuclear pore complex protein Nup153 OS=Homo sapiens GN=NUP153 PE=1 SV=2
R.N[+1378.476]VTWK.L	HexNAc(2)Hex(6)	N[+1378]	1013.420	2	2025.832	2025.827	2.8	Specific	267.7	267.7	267.7	4.17	>sp|P50454|SERPH_HUMAN Serpin H1 OS=Homo sapiens GN=SERPINH1 PE=1 SV=2
R.N[+1444.534]VTWK.L	HexNAc(4)Hex(3)Fuc(1)	N[+1445]	1046.447	2	2091.887	2091.885	1.0	Specific	163.0	77.7	77.7	0.81	>sp|P50454|SERPH_HUMAN Serpin H1 OS=Homo sapiens GN=SERPINH1 PE=1 SV=2

R.N[+1540.529]VTWK.L	HexNAc(2)Hex(7)	N[+1541]	1094.447	2	2187.886	2187.880	2.8	Specific	244.1	244.1	244.1	4.03	>sp|P50454|SERPH_HUMAN Serpin H1 OS=Homo sapiens GN=SERPINH1 PE=1 SV=2
R.N[+1702.581]VTWK.L	HexNAc(2)Hex(8)	N[+1703]	1175.471	2	2349.936	2349.933	1.3	Specific	272.5	272.5	272.5	2.78	>sp|P50454|SERPH_HUMAN Serpin H1 OS=Homo sapiens GN=SERPINH1 PE=1 SV=2
R.N[+1864.634]VTWK.L	HexNAc(2)Hex(9)	N[+1865]	1256.498	2	2511.989	2511.985	1.6	Specific	216.2	216.2	216.2	2.06	>sp|P50454|SERPH_HUMAN Serpin H1 OS=Homo sapiens GN=SERPINH1 PE=1 SV=2
R.SLSN[+1241.454]STAR.N	HexNAc(3)Hex(3)Fuc(1)	N[+1241]	1038.946	2	2076.886	2076.881	2.0	Specific	125.8	8.3	8.3	1.13	>sp|P50454|SERPH_HUMAN Serpin H1 OS=Homo sapiens GN=SERPINH1 PE=1 SV=2
R.SLSN[+1378.476]STAR.N	HexNAc(2)Hex(6)	N[+1378]	1107.458	2	2213.909	2213.903	3.0	Specific	228.1	60.1	60.1	1.31	>sp|P50454|SERPH_HUMAN Serpin H1 OS=Homo sapiens GN=SERPINH1 PE=1 SV=2
R.GIN[+1540.529]ESYKK.N	HexNAc(2)Hex(7)	N[+1541]	827.013	3	2479.023	2479.023	0.2	Specific	233.5	233.5	233.5	2.29	>sp|P50897|PPT1_HUMAN Palmitoyl-protein thioesterase 1 OS=Homo sapiens GN=PPT1 PE=1 SV=1
R.GIN[+1378.476]ESYKK.N	HexNAc(2)Hex(6)	N[+1378]	772.996	3	2316.972	2316.970	0.9	Specific	273.9	273.9	273.9	2.73	>sp|P50897|PPT1_HUMAN Palmitoyl-protein thioesterase 1 OS=Homo sapiens GN=PPT1 PE=1 SV=1
K.LHINHNN[+1768.640]LTESVGPLPK.S	HexNAc(4)Hex(5)Fuc(1)	N[+1769]	913.672	4	3651.667	3651.653	4.0	Specific	347.7	347.7	347.7	5.79	>sp|P51884|LUM_HUMAN Lumican OS=Homo sapiens GN=LUM PE=1 SV=2
R.GAFFPLTERN[+1768.640]WSLPNR.A	HexNAc(4)Hex(5)Fuc(1)	N[+1769]	919.161	4	3673.621	3673.616	1.2	Specific	116.5	116.5	116.5	0.01	>sp|P55058-4|PLTP_HUMAN Isoform 4 of Phospholipid transfer protein OS=Homo sapiens GN=PLTP
R.FN[+1216.423]GSVSFFR.G	HexNAc(2)Hex(5)	N[+1216]	1138.977	2	2276.946	2276.944	0.9	Specific	243.7	169.2	169.2	4.01	>sp|P55083|MFAP4_HUMAN Microfibril-associated glycoprotein 4 OS=Homo sapiens GN=MFAP4 PE=1 SV=2
R.FN[+1378.476]GSVSFFR.G	HexNAc(2)Hex(6)	N[+1378]	1220.004	2	2439.000	2438.997	1.4	Specific	308.4	198.9	198.9	4.06	>sp|P55083|MFAP4_HUMAN Microfibril-associated glycoprotein 4 OS=Homo sapiens GN=MFAP4 PE=1 SV=2
R.FN[+1378.476]GSVSFFR.G	HexNAc(2)Hex(6)	N[+1378]	1220.005	2	2439.003	2438.997	2.5	Specific	178.0	155.1	155.1	2.46	>sp|P55083|MFAP4_HUMAN Microfibril-associated glycoprotein 4 OS=Homo sapiens GN=MFAP4 PE=1 SV=2
R.FN[+1540.529]GSVSFFR.G	HexNAc(2)Hex(7)	N[+1541]	1301.031	2	2601.055	2601.050	2.1	Specific	100.0	87.6	87.6	0.91	>sp|P55083|MFAP4_HUMAN Microfibril-associated glycoprotein 4 OS=Homo sapiens GN=MFAP4 PE=1 SV=2
R.VDLEDFEN[+1768.640]NTAYAK.Y	HexNAc(4)Hex(5)Fuc(1)	N[+1769]	1133.136	3	3397.392	3397.383	2.5	Specific	53.2	53.2	53.2	2.32	>sp|P55083|MFAP4_HUMAN Microfibril-associated glycoprotein 4 OS=Homo sapiens GN=MFAP4 PE=1 SV=2
R.VDLEDFEN[+1768.640]NTAYAK.Y	HexNAc(4)Hex(5)Fuc(1)	N[+1769]	1133.133	3	3397.384	3397.383	0.0	Specific	132.2	132.2	132.2	2.19	>sp|P55083|MFAP4_HUMAN Microfibril-associated glycoprotein 4 OS=Homo sapiens

													GN=MFAP4 PE=1 SV=2
R.VDLEDFEN[+1768.640]NTAYAK.Y	HexNAc(4)Hex(5)Fuc(1)	N[+1769]	1133.133	3	3397.385	3397.383	0.4	Specific	327.8	325.7	325.7	6.69	>sp|P55083|MFAP4_HUMAN Microfibril-associated glycoprotein 4 OS=Homo sapiens GN=MFAP4 PE=1 SV=2
R.VDLEDFEN[+2059.735]NTAYAK.Y	HexNAc(4)Hex(5)Fuc(1)NeuAc(1)	N[+2060]	1230.166	3	3688.483	3688.479	1.2	Specific	159.8	159.8	159.8	3.25	>sp|P55083|MFAP4_HUMAN Microfibril-associated glycoprotein 4 OS=Homo sapiens GN=MFAP4 PE=1 SV=2
K.VVVVDGKN[+2350.830]ESEYTVK.W	HexNAc(4)Hex(5)Fuc(1)NeuAc(2)	N[+2351]	1004.939	4	4016.732	4016.700	8.1	Specific	111.1	1.6	1.6	0.02	>sp|P78536|ADA17_HUMAN Disintegrin and metalloproteinase domain-containing protein 17 OS=Homo sapiens GN=ADAM17 PE=1 SV=1
R.SLTQGSLIVGDLAPVN[+2059.735]GTSQGK.F	HexNAc(4)Hex(5)Fuc(1)NeuAc(1)	N[+2060]	1051.228	4	4201.888	4201.875	3.2	Specific	146.6	141.8	141.8	2.59	>sp|P98160|PGBM_HUMAN Basement membrane-specific heparan sulfate proteoglycan core protein OS=Homo sapiens GN=HSPG2 PE=1 SV=4
R.IQGEEIVFHDLN[+1768.640]LTAHGISHC[+57.021]PTC[+57.021]R.D	HexNAc(4)Hex(5)Fuc(1)	C[+57]*2, N[+1769]	935.415	5	4673.048	4673.027	4.3	Specific	121.0	69.6	69.6	2.29	>sp|P98160|PGBM_HUMAN Basement membrane-specific heparan sulfate proteoglycan core protein OS=Homo sapiens GN=HSPG2 PE=1 SV=4
R.ALVN[+1622.582]FTR.S	HexNAc(4)Hex(5)	N[+1623]	1222.033	2	2443.058	2443.049	3.5	Specific	101.0	101.0	101.0	0.23	>sp|P98160|PGBM_HUMAN Basement membrane-specific heparan sulfate proteoglycan core protein OS=Homo sapiens GN=HSPG2 PE=1 SV=4
R.LQILN[+349.137]ASSDVLR.I	HexNAc(1)Fuc(1)	N[+349]	839.450	2	1677.894	1677.891	1.8	Specific	673.7	673.7	673.7	10.72	>sp|Q02388-2|CO7A1_HUMAN Isoform 2 of Collagen alpha-1(VII) chain OS=Homo sapiens GN=COL7A1
R.LQILN[+349.137]ASSDVLR.I	HexNAc(1)Fuc(1)	N[+349]	839.450	2	1677.893	1677.891	1.3	Specific	583.6	583.6	583.6	9.25	>sp|Q02388|CO7A1_HUMAN Collagen alpha-1(VII) chain OS=Homo sapiens GN=COL7A1 PE=1 SV=2
K.TGEIN[+1216.423]ITSIVDR.E	HexNAc(2)Hex(5)	N[+1216]	1267.568	2	2534.129	2534.124	2.2	Specific	171.1	100.9	100.9	2.48	>sp|Q02413|DSG1_HUMAN Desmoglein-1 OS=Homo sapiens GN=DSG1 PE=1 SV=2
K.YIHQN[+1864.634]YTK.A	HexNAc(2)Hex(9)	N[+1865]	977.728	3	2931.170	2931.166	1.6	Specific	232.3	232.3	232.3	2.27	>sp|Q02809|PLOD1_HUMAN Procollagen-lysine,2-oxoglutarate 5-dioxygenase 1 OS=Homo sapiens GN=PLOD1 PE=1 SV=2
K.YIHQN[+1702.581]YTK.A	HexNAc(2)Hex(8)	N[+1703]	923.711	3	2769.119	2769.113	2.1	Specific	257.0	257.0	257.0	2.80	>sp|Q02809|PLOD1_HUMAN Procollagen-

													lysine,2-oxoglutarate 5-dioxygenase 1 OS=Homo sapiens GN=PLOD1 PE=1 SV=2
R.GN[+1768.640]GSRPSSPEEVK.F	HexNAc(4)Hex(5)Fuc(1)	N[+1769]	1038.106	3	3112.302	3112.295	2.4	Specific	135.5	22.2	22.2	0.94	>sp|Q05707|COEA1_HUMAN Collagen alpha-1(XIV) chain OS=Homo sapiens GN=COL14A1 PE=1 SV=3
K.INQFN[+0.984]LMASEMIALN[+406.159]R.S	HexNAc(2)	N[+1][+406]	1136.548	2	2272.088	2272.084	1.8	Specific	105.9	25.4	7.0	0.05	>sp|Q10472|GALT1_HUMAN Polypeptide N-acetylgalactosaminyltransferase 1 OS=Homo sapiens GN=GALNT1 PE=1 SV=1
R.NQMLISEDSRFEVFKN[+2059.735]GTLR.I	HexNAc(4)Hex(5)Fuc(1)NeuAc(1)	N[+2060]	1111.745	4	4443.960	4443.938	5.0	Specific	153.0	17.0	17.0	0.04	>sp|Q13308-2|PTK7_HUMAN Isoform 2 of Inactive tyrosine-protein kinase 7 OS=Homo sapiens GN=PTK7
R.NQMLISEDSRFEVFKN[+2350.830]GTLR.I	HexNAc(4)Hex(5)Fuc(1)NeuAc(2)	N[+2351]	1184.519	4	4735.055	4735.033	4.6	Specific	159.2	19.7	19.7	0.02	>sp|Q13308-2|PTK7_HUMAN Isoform 2 of Inactive tyrosine-protein kinase 7 OS=Homo sapiens GN=PTK7
R.TMVFPVMYLN[+1864.634]ESVHIDK.E	HexNAc(2)Hex(9)	N[+1865]	1296.553	3	3887.644	3887.637	1.9	Specific	177.1	177.1	177.1	3.11	>sp|Q14108-2|SCRB2_HUMAN Isoform 2 of Lysosome membrane protein 2 OS=Homo sapiens GN=SCARB2
K.VPAEILAN[+0.984]TSDNAGFC[+57.021]IPEGNC[+57.021]LGSGVLN[+552.217]VSIC[+57.021]K.N	HexNAc(2)Fuc(1)	C[+57]*3, N[+1][+552]	1376.978	3	4128.919	4129.914	2.0	Specific	30.0	11.4	11.4	0.02	>sp|Q14108-2|SCRB2_HUMAN Isoform 2 of Lysosome membrane protein 2 OS=Homo sapiens GN=SCARB2
R.TMVFPVMYLN[+1864.634]ESVHIDKETASR.L	HexNAc(2)Hex(9)	N[+1865]	1108.730	4	4431.900	4431.897	0.5	Specific	474.8	396.6	396.6	4.97	>sp|Q14108|SCRB2_HUMAN Lysosome membrane protein 2 OS=Homo sapiens GN=SCARB2 PE=1 SV=2
K.AFITN[+2204.772]FSMIIDGMTYPGIIK.E	HexNAc(4)Hex(5)Fuc(0)NeuAc(2)	N[+2205]	1479.650	3	4436.936	4436.917	4.3	Specific	114.5	114.5	114.5	1.18	>sp|Q14624|ITIH4_HUMAN Inter-alpha-trypsin inhibitor heavy chain H4 OS=Homo sapiens GN=ITIH4 PE=1 SV=4
K.AFITN[+2059.735]FSMIIDGMTYPGIIK.E	HexNAc(4)Hex(5)Fuc(1)NeuAc(1)	N[+2060]	1073.732	4	4291.904	4291.879	5.8	Specific	125.9	125.9	125.9	0.26	>sp|Q14624|ITIH4_HUMAN Inter-alpha-trypsin inhibitor heavy chain H4 OS=Homo sapiens GN=ITIH4 PE=1 SV=4
R.GHN[+1540.529]ESK.A	HexNAc(2)Hex(7)	N[+1541]	737.951	3	2211.838	2211.839	-0.7	Specific	162.3	162.3	162.3	0.26	>sp|Q15262-2|PTPRK_HUMAN Isoform 2 of Receptor-type tyrosine-protein phosphatase kappa OS=Homo sapiens GN=PTPRK
R.GHN[+1702.581]ESK.A	HexNAc(2)Hex(8)	N[+1703]	791.969	3	2373.892	2373.892	0.1	Specific	175.6	175.6	175.6	0.81	>sp|Q15262-2|PTPRK_HUMAN Isoform 2 of Receptor-type tyrosine-protein phosphatase kappa OS=Homo sapiens GN=PTPRK

K.N[+1913.677]LSK.P	HexNAc(4)Hex(5)Fuc(0)NeuAc(1)	N[+1914]	792.325	3	2374.959	2374.949	4.2	Specific	115.1	27.5	27.5	0.20	>sp|Q16363|LAMA4_HUMAN Laminin subunit alpha-4 OS=Homo sapiens GN=LAMA4 PE=1 SV=4
R.SQTFLEKHLN[+1702.581]SSHPR.S	HexNAc(2)Hex(8)	N[+1703]	697.504	5	3483.492	3483.490	0.4	Specific	356.2	356.2	356.2	4.19	>sp|Q5JTV8|TOIP1_HUMAN Torsin-1A-interacting protein 1 OS=Homo sapiens GN=TOR1AIP1 PE=1 SV=2
R.SQTFLEKHLN[+1913.677]SSHPR.S	HexNAc(4)Hex(5)Fuc(0)NeuAc(1)	N[+1914]	1232.204	3	3694.599	3694.586	3.4	Specific	205.4	205.4	205.4	0.07	>sp|Q5JTV8|TOIP1_HUMAN Torsin-1A-interacting protein 1 OS=Homo sapiens GN=TOR1AIP1 PE=1 SV=2
R.SQTFLEKHLN[+2204.772]SSHPR.S	HexNAc(4)Hex(5)Fuc(0)NeuAc(2)	N[+2205]	997.179	4	3985.696	3985.681	3.5	Specific	185.1	185.1	185.1	0.40	>sp|Q5JTV8|TOIP1_HUMAN Torsin-1A-interacting protein 1 OS=Homo sapiens GN=TOR1AIP1 PE=1 SV=2
R.QYRQAN[+1864.634]GSAK.K	HexNAc(2)Hex(9)	N[+1865]	996.410	3	2987.214	2987.199	5.0	Specific	171.5	54.5	54.5	0.64	>sp|Q5T5P2|SKT_HUMAN Sickle tail protein homolog OS=Homo sapiens GN=KIAA1217 PE=1 SV=2
K.QVLAN[+1913.677]KSHLWVEEEVWR.M	HexNAc(4)Hex(5)Fuc(0)NeuAc(1)	N[+1914]	1009.951	4	4036.780	4036.780	0.0	Specific	196.8	29.3	29.3	0.64	>sp|Q658P3|STEA3_HUMAN Metalloreductase STEAP3 OS=Homo sapiens GN=STEAP3 PE=1 SV=2
K.QVLAN[+2204.772]KSHLWVEEEVWR.M	HexNAc(4)Hex(5)Fuc(0)NeuAc(2)	N[+2205]	866.384	5	4327.891	4327.876	3.6	Specific	139.2	36.7	36.7	0.66	>sp|Q658P3|STEA3_HUMAN Metalloreductase STEAP3 OS=Homo sapiens GN=STEAP3 PE=1 SV=2
R.FVKN[+2350.830]ITSWK.E	HexNAc(4)Hex(5)Fuc(1)NeuAc(2)	N[+2351]	1158.493	3	3473.463	3473.461	0.6	Specific	133.0	133.0	133.0	0.44	>sp|Q7LGA3-2|HS2ST_HUMAN Isoform 2 of Heparan sulfate 2-O-sulfotransferase 1 OS=Homo sapiens GN=HS2ST1
R.NIRPNIILVLTDDQDVELGSM[+15.995]QVMN[+1913.677]K.T	HexNAc(4)Hex(5)Fuc(0)NeuAc(1)	M[+16], N[+1914]	1222.063	4	4885.229	4885.200	6.0	Specific	138.0	10.8	0.0	0.12	>sp|Q8IWU5-2|SULF2_HUMAN Isoform 2 of Extracellular sulfatase Sulf-2 OS=Homo sapiens GN=SULF2
K.EVLSSN[+2204.772]VSWRYEEQQLEIQNSSR.F	HexNAc(4)Hex(5)Fuc(0)NeuAc(2)	N[+2205]	1247.295	4	4986.159	4986.116	8.5	Specific	168.1	20.2	13.4	0.15	>sp|Q8IZF2|GP116_HUMAN Probable G-protein coupled receptor 116 OS=Homo sapiens GN=GPR116 PE=1 SV=3
R.FYN[+0.984]HPLHFVFN[+2204.772]DTK.L	HexNAc(4)Hex(5)Fuc(0)NeuAc(2)	N[+1][+2205]	996.912	4	3984.628	3984.621	1.6	Specific	120.6	120.6	120.6	0.62	>sp|Q8NE01-2|CNNM3_HUMAN Isoform 2 of Metal transporter CNNM3 OS=Homo sapiens

													GN=CNNM3
R.TTLVDNNTWN[+1864.634]NSHIALVGK.A	HexNAc(2)Hex(9)	N[+1865]	1321.245	3	3961.722	3961.707	3.8	Specific	361.6	320.2	5.9	5.69	>sp|Q8TCJ2|STT3B_HUMAN Dolichyl-diphosphooligosaccharide--protein glycosyltransferase subunit STT3B OS=Homo sapiens GN=STT3B PE=1 SV=1
R.TTLVDNNTWN[+1864.634]N[+0.984]SHIALVGK.A	HexNAc(2)Hex(9)	N[+1][+1865]	1321.576	3	3962.714	3962.691	6.0	Specific	215.0	215.0	2.1	2.46	>sp|Q8TCJ2|STT3B_HUMAN Dolichyl-diphosphooligosaccharide--protein glycosyltransferase subunit STT3B OS=Homo sapiens GN=STT3B PE=1 SV=1
R.YHYN[+1378.476]C[+57.021]SLLDGTQLFTSHDYGAPQEATLGANK.V	HexNAc(2)Hex(6)	C[+57], N[+1378]	1213.525	4	4851.079	4850.067	1.8	Specific	324.9	193.3	193.3	3.93	>sp|Q96AY3|FKB10_HUMAN Peptidyl-prolyl cis-trans isomerase FKBP10 OS=Homo sapiens GN=FKBP10 PE=1 SV=1
R.YHYN[+1378.476]GTFEDGKK.F	HexNAc(2)Hex(6)	N[+1378]	710.041	4	2837.141	2837.141	0.1	Specific	346.6	346.6	346.6	3.64	>sp|Q96AY3|FKB10_HUMAN Peptidyl-prolyl cis-trans isomerase FKBP10 OS=Homo sapiens GN=FKBP10 PE=1 SV=1
R.YHYN[+1702.581]GSLMDGTLFDSSYSR.N	HexNAc(2)Hex(8)	N[+1703]	1305.853	3	3915.543	3915.542	0.3	Specific	365.9	365.9	365.9	4.55	>sp|Q96AY3|FKB10_HUMAN Peptidyl-prolyl cis-trans isomerase FKBP10 OS=Homo sapiens GN=FKBP10 PE=1 SV=1
R.YHYN[+1702.581]GTLLDGTSFDTSYSK.G	HexNAc(2)Hex(8)	N[+1703]	1291.193	3	3871.566	3871.558	1.8	Specific	396.5	396.5	396.5	7.04	>sp|Q96AY3|FKB10_HUMAN Peptidyl-prolyl cis-trans isomerase FKBP10 OS=Homo sapiens GN=FKBP10 PE=1 SV=1
R.YHYN[+1864.634]GSLMDGTLFDSSYSR.N	HexNAc(2)Hex(9)	N[+1865]	1359.874	3	4077.607	4077.595	3.1	Specific	355.7	253.1	253.1	5.03	>sp|Q96AY3|FKB10_HUMAN Peptidyl-prolyl cis-trans isomerase FKBP10 OS=Homo sapiens GN=FKBP10 PE=1 SV=1
R.YHYN[+1864.634]GTLLDGTSFDTSYSK.G	HexNAc(2)Hex(9)	N[+1865]	1345.549	3	4034.632	4033.611	4.2	Specific	291.9	291.9	291.9	4.97	>sp|Q96AY3|FKB10_HUMAN Peptidyl-prolyl cis-trans isomerase FKBP10 OS=Homo sapiens GN=FKBP10 PE=1 SV=1
K.WGHN[+1378.476]ITEFQQR.F	HexNAc(2)Hex(6)	N[+1378]	932.061	3	2794.167	2794.157	3.6	Specific	194.1	194.1	194.1	1.39	>sp|Q96HE7|ERO1A_HUMAN ERO1-like protein alpha OS=Homo sapiens GN=ERO1L PE=1 SV=2
K.AMYVN[+1768.640]LTLTGEPIR.H	HexNAc(4)Hex(5)Fuc(1)	N[+1769]	1116.168	3	3346.489	3346.475	4.2	Specific	209.8	69.1	69.1	1.08	>sp|Q96PQ0|SORC2_HUMAN VPS10 domain-containing receptor SorCS2 OS=Homo sapiens GN=SORCS2 PE=1 SV=3
K.AMYVN[+2059.735]LTLTGEPIR.H	HexNAc(4)Hex(5)Fuc(1)NeuAc(1)	N[+2060]	910.152	4	3637.586	3637.571	4.2	Specific	189.5	29.3	29.3	0.25	>sp|Q96PQ0|SORC2_HUMAN VPS10 domain-containing receptor SorCS2 OS=Homo sapiens GN=SORCS2 PE=1 SV=3
K.AMYVN[+2350.830]LTLTGEPIR.H	HexNAc(4)Hex(5)Fuc(1)NeuAc(2	N[+2351]	982.923	4	3928.669	3928.666	0.7	Specific	223.2	32.3	32.3	0.41	>sp|Q96PQ0|SORC2_HUMAN VPS10 domain-

	)												containing receptor SorCS2 OS=Homo sapiens GN=SORCS2 PE=1 SV=3
K.N[+1378.476]MSFVNDLTVTQDGR.K	HexNAc(2)Hex(6)	N[+1378]	1025.767	3	3075.286	3075.272	4.6	Specific	129.0	129.0	124.1	0.36	>sp|Q9HDC9|APMAP_HUMAN Adipocyte plasma membrane-associated protein OS=Homo sapiens GN=APMAP PE=1 SV=2
K.FHLAN[+1768.640]R.T	HexNAc(4)Hex(5)Fuc(1)	N[+1769]	842.691	3	2526.060	2526.050	3.8	Specific	163.7	145.6	145.6	1.56	>sp|Q9NRN5|OLFL3_HUMAN Olfactomedin-like protein 3 OS=Homo sapiens GN=OLFML3 PE=2 SV=1

References:

Hennig, R., Cajic, S., Borowiak, M., Hoffmann, M., Kottler, R., Reichl, U., and Rapp, E. (2016). Towards personalized diagnostics via longitudinal study of the human plasma N-glycome. *Biochim Biophys Acta* 1860**,** 1728-1738.

Hennig, R., Rapp, E., Kottler, R., Cajic, S., Borowiak, M., and Reichl, U. (2015). N-Glycosylation Fingerprinting of Viral Glycoproteins by xCGE-LIF. *Methods Mol Biol* 1331**,** 123-143.
