# Supplementary material for: Alterations of the Human Skin N- and O-Glycome in Basal Cell Carcinoma and Squamous Cell Carcinoma
Source: Front Oncol. 2018 Mar 21;8:70. doi: 10.3389/fonc.2018.00070 (PMC5871710; doi:10.3389/fonc.2018.00070)

No	Glycan ID	m/z	z	M-H	theor. [M-H]-	delta[Da]	Core	Hex	HexNAc	Fuc	NeuAc	Structure	Category
1	**2**	**749.38**	1	749.38	749.27	0.11	1	-1					**paucimannosidic**
2	**3**	**840.80**	2	1682.6	1682.61	-0.01	1	1	3			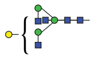	**bisecting**
3	**4**	**921.80**	2	1844.6	1844.67	-0.07	1	2	3			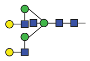	**bisecting**
4	**5**	**911.44**	1	911.44	911.32	0.12	1					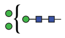	**paucimannosidic**
5	**6**	**911.44**	1	911.44	911.32	0.12	1					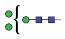	**paucimannosidic**
6	**7**	**832.83**	2	1666.66	1666.62	0.04	1		3	1		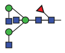	**bisecting**
7	**124**	**779.30**	2	1559.6	1559.53	0.07	1	4				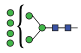	**Oligomannose**
8	**8**	**779.30**	2	1559.6	1559.53	0.07	1	4				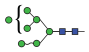	**Oligomannose**
9	**9**	**779.30**	2	1559.6	1559.53	0.07	1	4				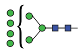	**Oligomannose**
10	**119**	**860.33**	2	1721.66	1721.59	0.07	1	5				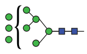	**Oligomannose**
11	**10**	**860.33**	2	1721.66	1721.59	0.07	1	5				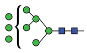	**Oligomannose**
12	**11**	**860.33**	2	1721.66	1721.59	0.07	1	5				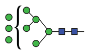	**Oligomannose**
13	**120**	**941.31**	2	1883.62	1883.64	-0.02	1	6				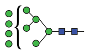	**Oligomannose**
14	**12**	**941.31**	2	1883.62	1883.64	-0.02	1	6				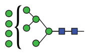	**Oligomannose**
15	**13**	**941.31**	2	1883.62	1883.64	-0.02	1	6				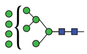	**Oligomannose**
16	**121**	**698.27**	2	1397.54	1397.48	0.06	1	3				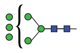	**Oligomannose**
17	**14**	**698.27**	2	1397.54	1397.48	0.06	1	3				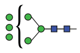	**Oligomannose**
18	**15**	**698.27**	2	1397.54	1397.48	0.06	1	3				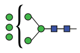	**Oligomannose**
19	**16**	**913.85**	2	1828.7	1828.67	0.03	1	1	3	1		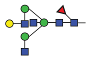	**Complex neutral, bisecting**
20	**107**	**913.85**	2	1828.7	1828.67	0.03	1	1	3	1		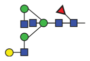	**Complex neutral, bisecting**
21	**17**	**1067.36**	2	2135.72	2135.76	-0.04	1	2	3		1	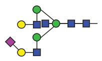	**Biantennary, singly sialylated, bisected**
22	**18**	**994.86**	2	1990.72	1990.72	0.00	1	2	3	1		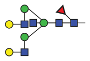	**Complex neutral**
23	**19**	**739.30**	2	1479.6	1479.53	0.07	1	1	2			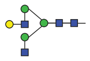	**Complex neutral**
24	**20**	**739.30**	2	1479.6	1479.53	0.07	1	1	2			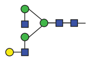	**Complex neutral**
25	**122**	**1073.36**	1	1073.36	1073.38	-0.02	1	1				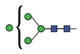	**Oligomannose**

No	Glycan ID	m/z	z	M-H	theor. [M-H]-	delta[Da]	Core	Hex	HexNAc	Fuc	NeuAc	Structure	Category
26	**21**	**1073.36**	1	1073.36	1073.38	-0.02	1	1				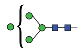	**Oligomannose**
27	**22**	**1022.30**	2	2045.6	2045.69	-0.09	1	7				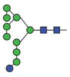	**Oligomannose**
28	**109**	**702.27**	2	1405.54	1405.50	0.04	1		1		1	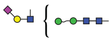	**Biantennary, singly sialylated**
29	**23**	**702.27**	2	1405.54	1405.50	0.04	1		1		1	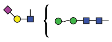	**Biantennary, singly sialylated**
30	**24**	**799.80**	2	1600.6	1600.56	0.04	1	3	1			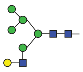	**hybrid**
31	**25**	**864.35**	2	1729.7	1729.60	0.10	1	2	1		1	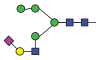	**Complex neutral**
32	**26**	**864.35**	2	1729.7	1729.60	0.10	1	2	1		1	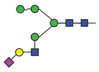	**Hybrid**
33	**27**	**820.32**	2	1641.64	1641.59	0.05	1	2	2			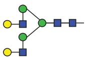	**Complex neutral**
34	**108**	**1140.40**	2	2281.8	2281.82	-0.02	1	2	3	1	1	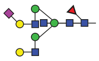	**Triantennary, singly sialylated**
35	**28**	**1140.40**	2	2281.8	2281.82	-0.02	1	2	3	1	1	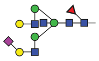	**Triantennary, singly sialylated**
36	**29**	**783.31**	2	1567.62	1567.55	0.07	1	1	1		1	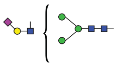	**Biantennary, singly sialylated**
37	**30**	**783.30**	2	1567.6	1567.55	0.05	1	1	1		1	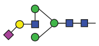	**Biantennary, singly sialylated**
38	**31**	**783.30**	2	1567.6	1567.55	0.05	1	1	1		1	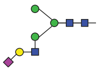	**Biantennary, singly sialylated**
39	**32**	**884.80**	2	1770.6	1770.63	-0.03	1	1	2		1	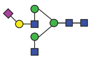	**Biantennary, singly sialylated**
40	**33**	**884.80**	2	1770.6	1770.63	-0.03	1	1	2		1	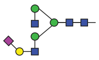	**Biantennary, singly sialylated**
41	**34**	**791.82**	2	1584.64	1584.57	0.07	1	2	1	1		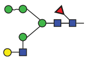	**Hybrid**
42	**35**	**731.28**	2	1463.56	1463.54	0.02	1		2	1		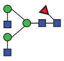	**Complex neutral**
43	**36**	**895.33**	1	895.33	895.33	0.00	1	-1		1		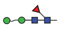	**paucimannosidic**
44	**37**	**945.33**	2	1891.66	1891.66	0.00	1	3	1		1	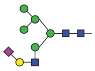	**Hybrid, Sialylated**
45	**38**	**945.33**	2	1891.66	1891.66	0.00	1	3	1		1	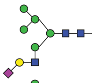	**Hybrid, Sialylated**
46	**39**	**965.82**	2	1932.64	1932.68	-0.04	1	2	2		1	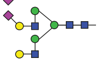	**Biantennary, singly sialylated**
47	**40**	**965.82**	2	1932.64	1932.68	-0.04	1	2	2		1	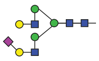	**Biantennary, singly sialylated**
48	**41**	**965.81**	2	1932.62	1932.68	-0.06	1	2	2		1	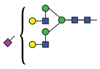	**Biantennary, singly sialylated**
49	**123**	**1235.48**	1	1235.48	1235.43	0.05	1	2				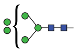	**Oligomannose**
50	**42**	**1235.48**	1	1235.48	1235.43	0.05	1	2				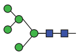	**Oligomannose**

No	Glycan ID	m/z	z	M-H	theor. [M-H]-	delta[Da]	Core	Hex	HexNAc	Fuc	NeuAc	Structure	Category
51	**43**	**812.33**	2	1625.66	1625.59	0.07	1	1	2	1		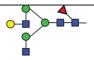	**Complex neutral**
52	**44**	**812.33**	2	1625.66	1625.59	0.07	1	1	2	1		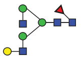	**Complex neutral**
53	**45**	**937.33**	2	1875.66	1875.66	0.00	1	2	1	1	1	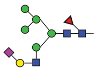	**Hybrid, Sialylated**
54	**46**	**937.33**	2	1875.66	1875.66	0.00	1	2	1	1	1	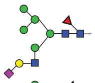	**Hybrid, Sialylated**
55	**47**	**1285.95**	2	2572.9	2572.92	-0.02	1	2	3	1	2	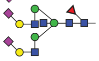	**bisecting, doubly sialylated**
56	**48**	**1285.95**	2	2572.9	2572.92	-0.02	1	2	3	1	2	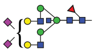	**bisecting, doubly sialylated**
57	**49**	**1285.95**	2	2572.9	2572.92	-0.02	1	2	3	1	2	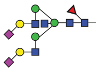	**bisecting, doubly sialylated**
58	**50**	**1148.38**	2	2297.76	2297.81	-0.05	1	3	3		1	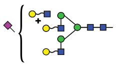	**Triantennary, singly sialylated**
59	**51**	**1148.38**	2	2297.76	2297.81	-0.05	1	3	3		1	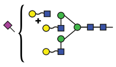	**Triantennary, singly sialylated**
60	**125**	**1148.38**	2	2297.76	2297.81	-0.05	1	3	3		1	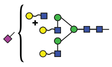	**Triantennary, singly sialylated**
61	**52**	**893.33**	2	1787.66	1787.65	0.01	1	2	2	1		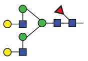	**Biantennary, singly sialylated**
62	**53**	**856.33**	2	1713.66	1713.61	0.05	1	1	1	1	1	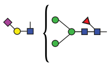	**singly sialylated**
63	**54**	**856.33**	2	1713.66	1713.61	0.05	1	1	1	1	1	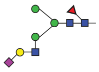	**singly sialylated**
64	**55**	**856.33**	2	1713.66	1713.61	0.05	1	1	1	1	1	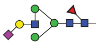	**singly sialylated**
65	**103**	**1111.45**	2	2223.9	2223.78	0.12	1	2	2		2	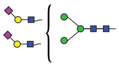	**Biantennary, doubly sialylated**
66	**56**	**1111.45**	2	2223.9	2223.78	0.12	1	2	2		2	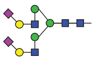	**Biantennary, doubly sialylated**
67	**61**	**1111.45**	2	2223.9	2223.78	0.12	1	2	2		2	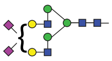	**Biantennary, doubly sialylated**
68	**63**	**1111.45**	2	2223.9	2223.78	0.12	1	2	2		2	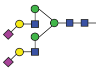	**Biantennary, doubly sialylated**
69	**102**	**1057.40**	1	1057.4	1057.38	0.02	1			1		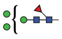	**Paucimannosidic**
70	**65**	**1057.40**	1	1057.4	1057.38	0.02	1			1		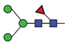	**Paucimannosidic**
71	**66**	**1038.85**	2	2078.7	2078.74	-0.04	1	2	2	1	1	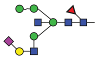	**singly sialylated**
72	**67**	**1038.85**	2	2078.7	2078.74	-0.04	1	2	2	1	1	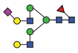	**singly sialylated**
73	**67**	**1038.85**	2	2078.7	2078.74	-0.04	1	2	2	1	1	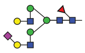	**singly sialylated**
74	**68**	**1038.85**	2	2078.7	2078.74	-0.04	1	2	2	1	1	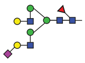	**singly sialylated**
75	**104**	**1221.43**	2	2443.86	2443.87	-0.01	1	3	3	1	1	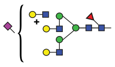	**triantenary singly sialylated**

No	Glycan ID	m/z	z	M-H	theor. [M-H]-	delta[Da]	Core	Hex	HexNAc	Fuc	NeuAc	Structure	Category
76	**69**	**1221.43**	2	2443.86	2443.87	-0.01	1	3	3	1	1	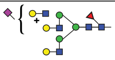	**Triantenary singly sialylated**
77	**72**	**1184.41**	2	2369.82	2369.84	-0.02	1	2	2	1	2	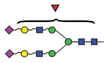	**Biantennary, doubly sialylated**
78	**73**	**1184.41**	2	2369.82	2369.84	-0.02	1	2	2	1	2	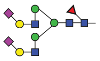	**Biantennary, doubly sialylated**
79	**74**	**1184.41**	2	2369.82	2369.84	-0.02	1	2	2	1	2	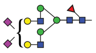	**Biantennary, doubly sialylated**
80	**76**	**1184.48**	2	2369.96	2369.84	0.12	1	2	2	1	2	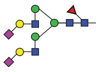	**Biantennary, doubly sialylated**
81	**79**	**1293.95**	2	2588.9	2588.91	-0.01	1	3	3		2	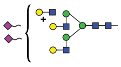	**triantenary singly sialylated**
82	**80**	**1293.95**	2	2588.9	2588.91	-0.01	1	3	3		2	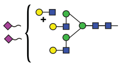	**triantenary singly sialylated**
83	**81**	**1293.95**	2	2588.9	2588.91	-0.01	1	3	3		2	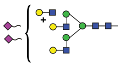	**triantenary singly sialylated**
84	**101**	**1293.95**	2	2588.9	2588.91	-0.01	1	3	3		2	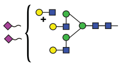	**triantenary singly sialylated**
85	**110**	**1293.95**	2	2588.9	2588.91	-0.01	1	3	3		2	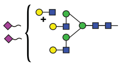	**triantenary singly sialylated**
86	**111**	**1293.95**	2	2588.9	2588.91	-0.01	1	3	3		2	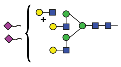	**triantenary singly sialylated**
87	**112**	**1293.95**	2	2588.9	2588.91	-0.01	1	3	3		2	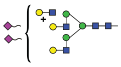	**triantenary singly sialylated**
88	**118**	**1293.95**	2	2588.9	2588.91	-0.01	1	3	3		2	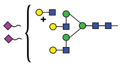	**triantenary singly sialylated**
89	**82**	**1221.43**	2	2443.86	2443.87	-0.01	1	3	3	1	1	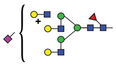	**triantenary singly sialylated**
90	**117**	**1221.43**	2	2443.86	2443.87	-0.01	1	3	3	1	1	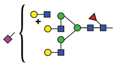	**triantenary singly sialylated**
91	**83**	**1439.48**	2	2879.96	2880.01	-0.05	1	3	3		3	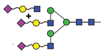	**triantenary triply sialylated**
92	**84**	**1439.48**	2	2879.96	2880.01	-0.05	1	3	3		3	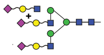	**triantenary triply sialylated**
93	**85**	**1439.48**	2	2879.96	2880.01	-0.05	1	3	3		3	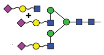	**triantenary triply sialylated**
94	**86**	**1366.97**	2	2734.94	2734.97	-0.03	1	3	3	1	2	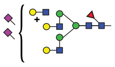	**triantenary doubly sialylated**
95	**87**	**1366.97**	2	2734.94	2734.97	-0.03	1	3	3	1	2	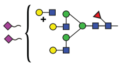	**triantenary doubly sialylated**
96	**88**	**1366.97**	2	2734.94	2734.97	-0.03	1	3	3	1	2	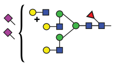	**triantenary doubly sialylated**
97	**89**	**1366.97**	2	2734.94	2734.97	-0.03	1	3	3	1	2	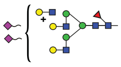	**triantenary doubly sialylated**
98	**90**	**1366.97**	2	2734.94	2734.97	-0.03	1	3	3	1	2	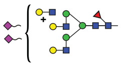	**triantenary doubly sialylated**
99	**106**	**1366.97**	2	2734.94	2734.97	-0.03	1	3	3	1	2	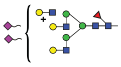	**triantenary doubly sialylated**
100	**113**	**1366.97**	2	2734.94	2734.97	-0.03	1	3	3	1	2	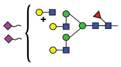	**triantenary doubly sialylated**

No	Glycan ID	m/z	z	M-H	theor. [M-H]-	delta[Da]	Core	Hex	HexNAc	Fuc	NeuAc	Structure	Category
101	**114**	**1366.97**	2	2734.94	2734.97	-0.03	1	3	3	1	2	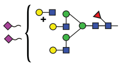	**triantenary doubly sialylated**
102	**115**	**1366.97**	2	2734.94	2734.97	-0.03	1	3	3	1	2	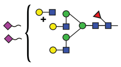	**triantenary doubly sialylated**
103	**116**	**1366.97**	2	2734.94	2734.97	-0.03	1	3	3	1	2	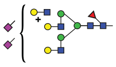	**triantenary doubly sialylated**
104	**91**	**1512.48**	2	3025.96	3026.06	-0.10	1	3	3	1	3	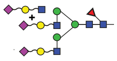	**triantenary triply sialylated**
105	**92**	**1512.48**	2	3025.96	3026.06	-0.10	1	3	3	1	3	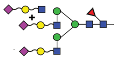	**triantenary triply sialylated**
106	**93**	**1512.48**	2	3025.96	3026.06	-0.10	1	3	3	1	3	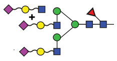	**triantenary triply sialylated**
107	**94**	**1512.48**	2	3025.96	3026.06	-0.10	1	3	3	1	3	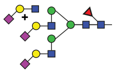	**triantenary triply sialylated**

Legend

- Mannose
- Galactose
- Glucose
- N-Acetylglucosamine
- N-Acetylgalactosamine
- N-Acetylneuraminic acid
- Fucose

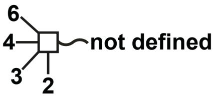

Supplement: Supplementary file 7 [file Table_1.PDF]
