# Supplementary material for: Alterations of the Human Skin N- and O-Glycome in Basal Cell Carcinoma and Squamous Cell Carcinoma
Source: Front Oncol. 2018 Mar 21;8:70. doi: 10.3389/fonc.2018.00070 (PMC5871710; doi:10.3389/fonc.2018.00070)

ID	m/z	z	M-H	theor. [M-H]-	delta	RT	red. GLcNAc	Hex	HexNAc	Fuc	NeuAc	NeuGc	HexA	Sulfate	Structure	Category
**1**	**755.3**	**1**	755.3	755.19	0.11	11.60	1	1			1			1	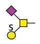	**core 1**
2	**675.30**	1	675.3	675.23	0.07	12.60	1	1			1				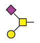	**core 1**
**3**	**675.30**	1	675.3	675.23	0.07	13.60	1	1			1				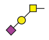	**core 1**
4	**749.37**	1	749.37	749.27	0.10	13.60	1	2	1						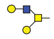	**core 2**
**5**	**966.38**	1	966.38	966.33	0.05	14.20	1	1			2				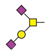	**core 1**
6	**1257.52**	1	1257.52	1257.42	0.10	15.00	1	1			3				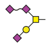	**core 1**
**7**	**895.40**	1	895.4	895.33	0.07	24.30	1	2	1	1					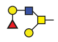	**core 2**
8	**1120.40**	1	1120.4	1120.32	0.08	21.40	1	2	1		1			1	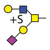	**core 2**
**9**	**1120.40**	1	1120.4	1120.32	0.08	27.70	1	2	1		1			1	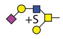	**core 2**
10	**1040.40**	1	1040.4	1040.36	0.04	21.40	1	2	1		1				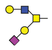	**core 2**
**11**	**1040.40**	1	1040.4	1040.36	0.04	27.70	1	2	1		1				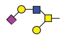	**core 2**
12	**1331.53**	1	1331.53	1331.46	0.07	32.90	1	2	1		2				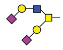	**core 2**
**13**	**705.26**	2	1411.52	1411.42	0.10	32.90	1	2	1		2			1	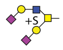	**core 2**

○ Galactose □ N-Acetylglucosamine ◆ N-Acetylneuraminic acid
■ N-Acetylglucosamine ▲ Fucose

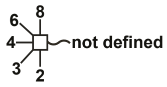

Supplement: Supplementary file 8 [file Table_2.PDF]
